# Resolving species boundaries in Mediterranean *Xiphinema* (Nematoda: Longidoridae) through multivariate morphometrics and multilocus profiling: four new species and two cryptic complexes

**DOI:** 10.1186/s40851-026-00269-4

**Published:** 2026-09-22

**Authors:** Rosana Salazar-García, Juan E. Palomares-Rius, Carolina Cantalapiedra-Navarrete, El Amine Ajal, Víctor Lechuga, Inmaculada Criado-Navarro, Pablo Castillo, Antonio Archidona-Yuste

**Affiliations:** 1 Department of Crop Protection, Institute for Sustainable Agriculture (IAS), Spanish National Research Council (CSIC), Avenida Menéndez Pidal s/n, Córdoba, 14004 Spain; 2 Programa de Doctorado de Ingeniería Agraria, Alimentaria, Forestal y del Desarrollo Rural Sostenible, Universidad de Córdoba, Córdoba, Spain; 3 UPR of Pharmacognosy, Faculty of Medicine and Pharmacy of Rabat, Mohammed V University, BP 6203, Rabat, 1000 Morocco

**Keywords:** COI mtDNA, D2-D3 of 28S rRNA, Partial 18S rRNA, ITS1 rRNA, Soil nematodes, Morphometrics analyses, Species delimitation

## Abstract

As a major biodiversity hotspot, the Mediterranean Basin provides an ideal setting for advancing our understanding of the diversity patterns of plant-parasitic nematodes. The genus *Xiphinema* is notably diverse in Mediterranean agroecosystems, yet species delimitation remains challenging due to the presence of cryptic lineages. Here, we address this issue by integrating morphology, morphometrics, and molecular data to characterise 50 *Xiphinema* populations from 264 sampling sites across agricultural landscapes and natural habitats in the main olive-growing regions of the Mediterranean Basin. Our findings yielded four major findings: (i) identification of four new species, *X. andalusomarocense* sp. nov. *X. africana* sp. nov. *X. alentejanum* sp. nov. and *X. cryptoduriense* sp. nov.; (ii) discovery of two well-supported cryptic complexes by combining multivariate morphometric analyses, multilocus molecular profiling, and phylogenetic inference (*X. baetica-* and *X. duriense*-complexes); (iii) demonstration of the diagnostic value of morphological variation in size and shape for delimiting species within cryptic complexes; and iv) the generation of new ribosomal and mitochondrial sequences for 18 *Xiphinema* species, expanding the molecular resources available for this group. Together, these results refine the systematics of *Xiphinema*, broaden our understanding of nematode diversity in the Mediterranean Basin, and provide a robust framework for future evolutionary research on *Xiphinema*.

**Supplementary Information:**

The online version contains supplementary material available at https://doi.org/10.1186/s40851-026-00269-4.

## Background

Ectoparasitic migratory nematodes of the genus *Xiphinema* Cobb, 1913, are distinguished by their moderate to long bodies and conspicuous odontostyle. More than 315 valid species are currently recognised worldwide, with diversity unevenly distributed between two major species complexes. The *X.* non‑*americanum*-group exhibits the greatest morphological and biogeographical breadth comprising more than 250 distinguishable species [1], whereas the *X. americanum*-group remains morphologically conservative and taxonomically problematic, encompassing approximately 65 nominal species [2]. Together, these assemblages represent one of the most challenging taxonomic lineages within Longidoridae, owing to their morphological conservatism, extensive cryptic diversity, and persistence of species complexes that defy straightforward delimitation [3]. These difficulties are further compounded by the ecological and agricultural importance of *Xiphinema* species, which act as ectoparasites and vectors of nepoviruses in economically significant crops [4–8]. Despite repeated taxonomic revisions, species boundaries within both the *X.* non-*americanum* and *X. americanum*-groups remain unclear, largely due to the scarcity of reliable morphological characters and the prevalence of cryptic or near-cryptic species [7–9].

Over the past two decades, substantial progress in *Xiphinema* systematics has been achieved through integrative taxonomic approaches. The combined use of classical morphology, multivariate morphometrics, and multilocus sequencing, particularly of ribosomal and mitochondrial markers, has revealed numerous previously unrecognised lineages and demonstrated that many traditional morphospecies comprise multiple, independently evolving entities. For example, studies on the *Xiphinema hispanum* Lamberti et al. 1992 [10] complex have shown that morphostatic speciation is widespread in southern European lineages, necessitating the integration of morphological and molecular evidence for robust species delimitation [3, 7, 8]. Comparable patterns have been documented within the *X. americanum*‑group, where integrative analyses have clarified relationships within the *X. pachtaicum* [11] Kirjanova, 1951 [12] subgroup and uncovered substantial cryptic diversity [8]. More recent studies have further highlighted the value of multilocus datasets, including the D2–D3 expansion segments of 28S rRNA, ITS1 rRNA, partial 18S rRNA, and cytochrome c oxidase subunit 1 (COI), in refining species hypotheses and documenting overlooked diversity across both monodelphic and pseudomonodelphic lineages [13].

The taxonomy of *Xiphinema* remains far from resolved because of the great diversity found in this genus, which includes numerous species of agronomic importance. These nematodes are particularly problematic in perennial cropping systems, where they cause direct root damage and/or act as vectors of nepoviruses, posing a persistent threat to crop productivity [14]. Olive (*Olea europaea* L.) cultivation, a defining feature of agricultural landscapes throughout the Mediterranean Basin, provides a patchwork of managed groves interspersed with semi‑natural and natural habitats that harbour rich nematode assemblages [15, 16]. This ecological mosaic may facilitate the coexistence of both widespread and endemic *Xiphinema* species. Previous regional surveys have shown that olive agroecosystems can support a diverse array of *Xiphinema* species, including both widespread taxa and narrowly distributed endemics. However, many populations remain insufficiently characterised or entirely undescribed. As a global biodiversity hotspot [17], the Mediterranean Basin is therefore of particular importance for advancing our understanding of *Xiphinema* diversity, biogeography, and evolutionary history.

In this study, we conducted an extensive survey of *Xiphinema* populations across the Mediterranean olive-growing area, including both olive groves and adjacent natural habitats in Greece, Morocco, Portugal, and Spain. Our objectives were: (i) to undertake detailed morphological and morphometric characterisation of the collected *Xiphinema* populations and compare them with known congeners; (ii) to characterise these populations molecularly using D2–D3 segments of 28S rRNA, ITS1 rRNA, partial 18S rRNA, and COI gene sequences; and (iii) to infer the phylogenetic relationships within the context of currently available *Xiphinema* molecular data. Through this integrative approach, we described four new species, three of which belong to the *X.* non-*americanum* group (i.e. *X. andalusomarocense* sp. nov., *X. africana* sp. nov. and *X. alentejanum* sp. nov.) and one to the *X. americanum* group (i.e. *X. cryptoduriense* sp. nov.); and provided new insights into species boundaries and evolutionary patterns within these taxonomically complex nematode groups.

## Materials

### Sampling nematode populations and morphological studies

Nematode surveys were conducted during the olive flowering period in spring 2023, encompassing 264 sampling sites distributed across 44 olive groves and adjacent patches of natural vegetation. These sites were located within key olive-producing regions of the Mediterranean Basin, including Greece, Morocco, Portugal, and Spain. At each site, soil samples were collected from both the cultivated olive grove and the nearest patch of natural vegetation, ensuring identical environmental conditions across paired samples (Table 1). Soil samples were collected from the rhizosphere of host-plants using a hoe, targeting the upper 50 cm of soil beneath randomly selected plants at each sampling site. From each soil sample, nematodes were extracted from 500 cm^3^ of soil by centrifugal flotation and modification of Cobb’s methods [18, 19]. Following a preliminary morphological examination at the genus level, we focused on the populations of dagger nematodes of the genus *Xiphinema*. As a result, we identified a total of 50 nematode populations belonging to this genus (Table 1). Nematodes were identified at the species level by applying an integrative approach integrating morphological analyses and molecular analyses to achieve an efficient and accurate identification [3, 7]. For morphological study, specimens were killed by gentle heat, fixed in a solution of 4% formaldehyde + 1% propionic acid, and processed to pure glycerine using Seinhorst’s method [20]. Light micrographs from each nematode species were taken using a Leica DM6 compound microscope with a Leica DFC7000 T digital camera (Wetzlar, Germany) using mounted nematodes in glycerine.

**Table 1:** Localities of the analysed populations of *Xiphinema* Cobb 1913 species from the rhizosphere of olive trees in the Mediterranean Basin

Species	Sample code	Location, province (country)	Host Plant	NCBI Accessions			
				D2–D3	ITS1	18S	COI
***Xiphinema *non*-americanum*-group species**							
*Xiphinema andalusomarocense* sp. nov.	SO36	Ait Taleb, Azilal (Morocco)	*Olea europaea* ssp. *europaea* L. (cultivated olive)	PZ458835–PZ458840	PZ458570–PZ458572	PZ458890	PZ464576–PZ464578
*Xiphinema andalusomarocense* sp. nov.	AR31	Tarifa, Cádiz (Spain)	*Olea europaea* ssp. *sylvestris* Brot. (wild olive)	PZ458841–PZ458843	PZ458573	PZ458891	PZ464579
*Xiphinema andalusomarocense* sp. nov.	RET2	Chiclana de Segura, Jaén (Spain)	*Retama sphaerocarpa* (L.) Boiss. (yellow broom)	PZ458844–PZ458847	PZ458574–PZ458575	PZ458892	PZ464580–PZ464581
*Xiphinema africana* sp. nov.	ZN13	Demnate, Azilal (Morocco)	*Chamaerops humilis* L. (Mediterranean fan palm)	PZ458848–PZ458850	PZ458576–PZ458580	PZ458893–PZ458894	PZ464582–PZ464584
*Xiphinema africana* sp. nov.	SO38	Al Manzah, Meknes (Morocco)	*Olea europaea* ssp. *europaea* L. (cultivated olive)	PZ458851–PZ458856	PZ458581	-	PZ464585–PZ464587
*Xiphinema alentejanum* sp. nov.	SO22	Ferreira do Alentejo, Beja (Portugal)	*Quercus ilex* L. (holly oak)	PZ458857–PZ458861	PZ458582–PZ458585	PZ458895–PZ458897	PZ464588–PZ464592
*Xiphinema adenohystherum*	SO21	Ferreira do Alentejo, Beja (Portugal)	*Retama sphaerocarpa* (L.) Boiss. (yellow broom)	PZ458862	-	-	PZ464593–PZ464594
*Xiphinema adenohystherum*	SO22	Ferreira do Alentejo, Beja (Portugal)	*Quercus ilex* L. (holly oak)	-	-	-	-
*Xiphinema adenohystherum*	RET2	Chiclana de Segura, Jaén (Spain)	*Retama sphaerocarpa* (L.) Boiss. (yellow broom)	-	-	-	-
*Xiphinema ingens*	AN3a	Skala Sykamineas Lesbos (Greece)	Turkish pine (*Pinus brutia* Ten.)	PZ458863	-	-	-
*Xiphinema israeliae*	SO30	Tympaki, Crete (Greece)	*Olea europaea* ssp. *europaea* L. (cultivated olive)	-	-	-	PZ464595
*Xiphinema italiae*	SO04	Antequera, Malaga (Spain)	*Olea europaea* ssp. *europaea* L. (cultivated olive)	-	-	-	-
*Xiphinema italiae*	ZN42	Meknes, Fez-Mequines (Morocco)	*Opuntia ficus indica* (L.) Mill. (Indian fig opuntia)	PZ458864	PZ458586	-	-
*Xiphinema italiae*	ZN43	Meknes, Fez-Mequines (Morocco)	*Olea europaea* ssp. *sylvestris* Brot. (wild olive)	-	-	-	-
*Xiphinema nuragicum*	SO07	Villanueva del Arzobispo, Jaén (Spain)	*Olea europaea* ssp. *europaea* L. (cultivated olive)	-	-	-	-
*Xiphinema nuragicum*	SO08	Villacarrillo, Jaén (Spain)	*Olea europaea* ssp. *europaea* L. (cultivated olive)	-	-	-	-
*Xiphinema nuragicum*	SO09	Santisteban del Pto, Jaén (Spain) (Spain)	*Olea europaea* ssp. *europaea* L. (cultivated olive)	-	-	-	-
*Xiphinema nuragicum*	SO12	Carcabuey, Córdoba (Spain)	*Olea europaea* ssp. *europaea* L. (cultivated olive)	-	-	-	-
*Xiphinema nuragicum*	SO14	Monturque, Córdoba (Spain)	*Olea europaea* ssp. *europaea* L. (cultivated olive)	-	-	-	-
*Xiphinema nuragicum*	SO20	Braganza, Braganza (Portugal)	*Olea europaea* ssp. *europaea* L. (cultivated olive)	PZ458865	-	-	PZ464596
*Xiphinema nuragicum*	SO20	Braganza, Braganza (Portugal)	*Quercus ilex* L. (holly oak)		-	-	-
*Xiphinema sphaerocephalum*	SO18	Mirandela, Braganza (Portugal)	*Olea europaea* ssp. *europaea* L. (cultivated olive)	PZ458866	-	-	-
*Xiphinema turcicum*	SO05	Chiclana de Segura, Jaén (Spain)	*Olea europaea* ssp. *europaea* L. (cultivated olive)	PZ458867	-	-	-
*Xiphinema turcicum*	SO06	Cañete de las Torres, Córdoba (Spain)	*Olea europaea* ssp. *europaea* L. (cultivated olive)	-	-	-	-
*Xiphinema zagrosense*	AN2b	Plomari, Lesbos (Greece)	Turkish pine (*Pinus brutia* Ten.)	PZ458868	PZ458587–PZ458588	-	-
*Xiphinema* sp. SO20	SO20	Braganza, Braganza (Portugal)	*Olea europaea* ssp. *europaea* L. (cultivated olive)	PZ458869, PZ458871	PZ458589	PZ458898–PZ458899	PZ464597–PZ464598
*Xiphinema* sp. SO20	SO20	Braganza, Braganza (Portugal)	*Quercus ilex* L. (holly oak)	PZ458870	PZ458590	-	PZ464599–PZ464600
***Xiphinema americanum*-group species**							
*Xiphinema cryptoduriense* sp. nov.	SO18	Mirandela, Braganza (Portugal)	*Olea europaea* ssp. *europaea* L. (cultivated olive)	PZ458872–PZ458877	PZ458591–PZ458594	PZ458900–PZ458901	PZ464601–PZ464602
*Xiphinema incertum*	SO04	Antequera, Malaga (Spain)	*Olea europaea* ssp. *europaea* L. (cultivated olive)	-	-	-	-
*Xiphinema incertum*	SO06	Cañete de las Torres, Córdoba (Spain)	*Olea europaea* ssp. *europaea* L. (cultivated olive)	-	-	-	-
*Xiphinema incertum*	SO36	Ait Taleb, Azilal (Morocco)	*Olea europaea* ssp. *europaea* L. (cultivated olive)	PZ458879	PZ487679, PZ487680	-	-
*Xiphinema incertum*	ZN13	Demnate, Azilal (Morocco)	*Pinus nigra* J.F.Arnold (black pine)	PZ458878	-		
*Xiphinema luci*	SO37	Ait Ichou, Azilal (Morocco)	*Olea europaea* ssp. *europaea* L. (cultivated olive)	PZ458880	-	-	PZ464604
*Xiphinema luci*	SO39	Ouarjdan, Fkih Ben Salah (Morocco)	*Olea europaea* ssp. *europaea* L. (cultivated olive)	PZ458881–PZ458882	-	-	PZ464605–PZ464606
*Xiphinema pachtaicum*	SO01	Sabiote, Jaén (Spain)	*Olea europaea* ssp. *europaea* L. (cultivated olive)	PZ458883	-	-	-
*Xiphinema pachtaicum*	SO02	Cañadas de Pareja, Málaga (Spain)	*Olea europaea* ssp. *europaea* L. (cultivated olive)	-	-	-	-
*Xiphinema pachtaicum*	SO07	Villanueva del Arzobispo (Spain)	*Olea europaea* ssp. *europaea* (cultivated olive)	-	-	-	-
*Xiphinema pachtaicum*	SO09	Santisteban del Pto, Jaén (Spain)	*Olea europaea* ssp. *europaea* L. (cultivated olive)	-	-	-	-
*Xiphinema pachtaicum*	SO18	Mirandela, Braganza (Portugal)	*Olea europaea* ssp. *europaea* L. (cultivated olive)	PZ458884	-	-	-
*Xiphinema pachtaicum*	SO20	Braganza, Braganza (Portugal)	*Olea europaea* ssp. *europaea* L. (cultivated olive)	-	-	-	-
*Xiphinema pachtaicum*	SO22	Ferreira do Alentejo (Portugal)	*Quercus ilex* L. (holly oak)	-	-	-	-
*Xiphinema pachtaicum*	SO27	Aristomensis, Messenia (Greece)	*Olea europaea* ssp. *europaea* L. (cultivated olive)	PZ458885	-	-	-
*Xiphinema pachtaicum*	SO31	Moires, Crete (Greece)	*Olea europaea* ssp. *europaea* L. (cultivated olive)	-	-	-	-
*Xiphinema pachtaicum*	SO34	Meknes, Fez-Mequines (Morocco)	*Olea europaea* ssp. *europaea* L. (cultivated olive)	-	-	-	-
*Xiphinema pachtaicum*	SO36	Ait Taleb, Azilal (Morocco)	*Olea europaea* ssp. *europaea* L. (cultivated olive)	PZ458886	-	-	-
*Xiphinema pachtaicum*	SO40	Paleokipos, Lesbos (Greece)	*Olea europaea* ssp. *europaea* L. (cultivated olive)	-	-	-	-
*Xiphinema pachtaicum*	ZN13	Demnate, Azilal (Morocco)	*Pinus nigra* J.F.Arnold (black pine)	-	-	-	-
*Xiphinema plesiopachtaicum*	SO04	Antequera, Malaga (Spain)	*Olea europaea* ssp. *europaea* L. (cultivated olive)	PZ458887	-	-	-
*Xiphinema plesiopachtaicum*	SO18	Mirandela, Braganza (Portugal)	*Olea europaea* ssp. *europaea* L. (cultivated olive)	PZ458888	PZ487681	-	-
*Xiphinema* sp. *americanum*-group SO20	SO20	Braganza, Braganza (Portugal)	*Quercus ilex* L. (holly oak)	PZ458889	-	-	PZ464603

^a^ = not sequenced or not submitted to GenBank

### DNA extraction, PCR and sequencing

All 50 *Xiphinema* populations detected in this study were subjected to molecular characterisation. In each case, DNA extraction was performed from individual specimens, ensuring that all molecular markers analysed originated from the same single DNA-extracted specimen per PCR tube, thereby eliminating potential errors. To prevent misidentification in cases where multiple *Xiphinema* populations co-occurred within the same soil sample (Table 1), single nematodes were temporarily placed in a drop of 1 M NaCl containing glass beads, preventing physical damage while confirming specimen identity within the unidentified population.

Nematode DNA extraction followed the protocol described by Subbotin et al. [21]. Briefly, individual nematodes were cut with a scalpel in a 20 µL drop of PCR buffer (ThermoPol®, Biolabs, New England, USA), to which 2 μL of proteinase K (600 μg/mL) was added. The tubes were frozen at − 80 °C for 15 minutes, followed by consecutive incubations at 65 °C for 1 hour and 95 °C for 10 minutes. Samples were then centrifuged at 16,000× g for 1 minute and stored at − 20 °C until use in PCR.

The D2–D3 expansion segments of the 28S rRNA gene were amplified using primers D2A (5’-ACAAGTACCGTGAGGGAAAGTTG-3’) and D3B (5’-TCGGAAGGAACCAGCTACTA-3’) [22]. The ITS1 region was amplified using the forward primer 18S (5′-TTGATTACGTCCCTGCCCTTT-3′) and the reverse primer Rdna1 (5′-ACGAGCCGAGTGATCCACCG-3′) [23]. The partial 18S rRNA gene was amplified using primers 988F (5′-CTCAAAGATTAAGCCATGC-3′), 1912 R (5′-TTTACGGTCAGAACTAGGG-3′), 1813F (5′-CTGCGTGAGAGGTGAAAT-3′), and 2646 R (5′-GCTACCTTGTTACGACTTTT-3′) [24]. The mitochondrial COI gene fragment was amplified using primers COIF (5′-GATTTTTTGGKCATCCWGARG-3′) and XIPHR2 (5′-GTACATAATGAAAATGTGCCAC-3′) following the protocol of Lazarova et al. [25].

All PCR assays were performed under the conditions described by Archidona-Yuste et al. [7]. Amplified PCR products were subsequently purified using ExoSAP-IT (Affymetrix, USB Products) and sequenced on a Applied Biosystems 3130xl Genetic Analyzer (Applied Biosystems, Foster City, CA, USA) using the BigDye Terminator Sequencing Kit v.3.1 (Applied Biosystems). Sequencing was conducted at the StabVida facility (Costa da Caparica, Portugal).

Newly obtained sequences were submitted to the National Center for Biotechnology Information (NCBI) under accession numbers listed in Table 1 and associated phylogenetic trees. Sequence chromatograms for all four markers (D2–D3 expansion segments of 28S, ITS1, 18S rRNA, and COI mtDNA) were assembled and edited using DNASTAR LASERGENE SeqMan v.7.1.0. Species identity of the DNA sequences was confirmed using BLAST [26].

### Species delimitation within *Xiphinema* non-*americanum*-group and *Xiphinema americanum*-group populations

From the 50 *Xiphinema* populations identified in this study, a small number exhibited a notable morphological similarity with already described taxa, but they were found to be genetically distinct from these species by molecular markers. One population belonging to the *Xiphinema* non-*americanum* group showed a strong morphological resemblance to *Xiphinema baetica* Gutiérrez-Gutiérrez et al. 2013 [6], sharing most of its diagnostic features, although preliminary molecular data suggested that it represents a different taxon. Likewise, molecular screening of a second population from the *Xiphinema americanum*-group revealed marked genetic divergence from *X. duriense* Lamberti et al. 1983 [27–29], despite the absence of clear morphological differences. These findings prompted us to establish a framework for delimiting species among the newly identified *Xiphinema* populations that could be putative new species for science (here named as *Xiphinema alentejanum* sp. nov. and *Xiphinema cryptoduriense* sp. nov.), and to evaluate their relationships with taxa to which they were morphologically most similar (*X. baetica* and *X. duriense*, respectively). For *X. baetica* species complex, we included *X. alentejanum* sp. nov., *X. baetica* Gutiérrez-Gutiérrez et al. 2013 [6], *X. andalusiense* Archidona-Yuste et al. 2016 [7], and *X. macrodora* Archidona-Yuste et al. 2016 [30]. And for the *X. duriense*-complex, we included *X. cryptoduriense* sp. nov., *X. duriense* and *X. opisthohysterum* Siddiqi, 1961 [31]. Species boundaries within these two apparent cryptic complexes were assessed using complementary morphometric and molecular approaches. To ensure direct comparability between methods and with the phylogenetic inferences, only populations for which both morphological and molecular datasets were available were included in the analyses.

#### Morphometric species delimitation

Two independent morphometric analyses were carried out on each cryptic complex within the *X.* non-*americanum*-group and *X. americanum*-group using Principal Component Analysis (PCA) [32]. In addition to the populations mentioned above, further populations of closely related species were included to enhance the consistency and reliability of the morphometric assessments. For the *X. baetica* species complex, we considered additional populations of closely related species belonging to the *X.* non-*americanum* group 5 [33]. In total, the dataset comprised one population each of *X. alentejanum* sp. nov., *X. andalusiense*, *X. baetica*, *X. cadavalense* Bravo & Roca, 1995 [34], *X. coxi europaeum* Sturhan, 1984 [35], *X. macrodora*, and *X. pseudocoxi* [6, 7, 30]. Similarly, we included some additional populations of closely related species in the case of *X. duriense*-complex. In this case, we have included a total of: one population of *X. cryptoduriense* sp. nov., *X. duriense*, *X. madeirense*, *X. opisthohysterum*, *X. pachtaicum*, *X. plesiopacthaicum* [7, 8]. Exceptionally, several uncharacterised populations at the molecular level of *X. duriense* were included for comparison with the sequenced populations used in our study [27]. As far as the data allowed, the morphometrics database consisted of a roughly equal number of individuals for each species included in the analysis.

In both cryptic complexes, multivariate analyses were performed using the main morphometric characters used in the taxonomic keys within the genus *Xiphinema* [4, 33]. These characters included: body length (L), the ratios a, c′ and V [(distance from anterior end to vulva/body length) × 100], odontostyle length, oral aperture-guiding ring distance, and lip region width. Prior to analysis, morphometric variables were standardised using z-score transformation. We then tested to collinearity among variables using Variance Inflation Factor (VIF) values [36]. PCA was then conducted using the package FactoMineR [37]. Finally, to evaluate interspecific differences while accounting for intraspecific variability within each complex, we applied linear mixed-effect model (LMM) following Peraza-Padilla et al. [38]. Principal components PC1 and PC2 were used as response variables, species was included as a fixed explanatory factor, and population was incorporated as random intercept effect. Models were fitted using the lme4 package [39]. All analyses were conducted in R version 4.4.2 (R Core Team 2024; https://www.R-project.org).

#### Molecular species delimitation

Molecular delimitation of the *X. alentejanum* sp. nov., *X. baetica*, and *X. cryptoduriense* sp. nov., *X. duriense* complexes were conducted using ribosomal and mitochondrial markers, including the D2–D3 expansion segments of the 28S rRNA, the ITS1 rRNA region, and the COI mtDNA barcoding locus. Analyses were performed with the species-delimitation plugin [40] implemented in Geneious Prime v2026.0.2. (Geneious, Auckland, New Zealand), which estimates intra- and interspecific divergence using the PID (liberal) and Rosenberg’s P_AB_ statistics. Intraspecific and interspecific variation were quantified by calculating the ratio between the mean pairwise genetic distance among individuals within a putative species and the mean distance between those individuals and their closest related species. Ratios < 0.10 indicate a high likelihood of correct species identification [40]. The PID (Liberal) statistic [41] estimates the probability of accurate species assignment based on best sequence match, minimum genetic distance, or phylogenetic placement; values ≥ 0.93 were interpreted as strong support for species boundaries [42]. Rosenberg’s P_AB_ evaluates whether observed monophyly is unlikely to have arisen by chance, with significant support at *p* < 0.01 [43].

### Phylogenetic analysis

D2–D3 expansion domains of the 28S, ITS1, 18S rRNA, and COI mtDNA sequences of the unidentified *Xiphinema* species recovered in this study were generated and analysed. These sequences, together with additional *Xiphinema* spp. sequences retrieved from GenBank were used for phylogenetic reconstruction. Outgroup taxa for each dataset were selected following previously published studies [3, 7, 13, 38]. Multiple sequence alignments for each gene were performed using the FFT-NS-2 algorithm implemented in MAFFT V.7.450 [44]. Alignment was visualised in BioEdit program V.7.2.5 [45] and manually edited, with poorly aligned regions trimmed using a light-filtering strategy (up to 20% of alignment positions). This approach has minimal impact on phylogenetic accuracy while reducing computational burden, as recommended by Tan et al. [46], who demonstrated that automated filtering methods often degrade single-gene phylogenetic inference.

Phylogenetic analyses were conducted using Bayesian inference (BI) in MrBayes 3.1.2 [47]. The best-fit model of nucleotide substitution for each dataset was determined using JModelTest V.2.1.7 [48] under the Akaike Information Criterion (AIC). The selected substitution model, base frequencies, proportion of invariable sites, gamma-shape parameter, and rate matrices were subsequently implemented in MrBayes. The following models were applied: general time-reversible model with invariable sites and a gamma-shaped distribution (GTR + I + G) for the D2–D3 expansion segments of 28S rRNA (*X.* non-*americanum*-group species dataset); general time-reversible model and a gamma-shaped distribution (GTR + G) for the ITS1 rRNA region of the *X. alentejanum* sp. nov. species dataset; the Tamura and Nei model with invariable sites and a gamma-shaped distribution (TRN + I + G) for the ITS1 rRNA region of the *X. andalusomarocense* sp. nov. and *X. africana* sp. nov. species dataset; the symmetrical model with invariable sites and a gamma-shaped distribution (SYM + I + G) for the partial 18S rRNA gene (*X.* non-*americanum*-group and *X. americanum*-group species dataset); the transversion model with invariable sites and a gamma shaped distribution (TVM + I + G) for the partial COI gene (*X.* non-*americanum*-group species dataset); the general time-reversible model with a gamma-shaped distribution (GTR + G) for the D2–D3 expansion segments of 28S rRNA (*X. americanum*-group species dataset); and the three parameter model with invariable sites and a gamma shaped distribution (TPM1uf + I + G) for the partial COI gene (*X. americanum*-group species dataset). Each analysis was run with four Markov chains for 10 × 10^6^ generations, sampling every 100 generations. Two independent runs were performed for each dataset. After discarding the first 30% of samples as burn-in and confirming convergence, the remaining trees were used to construct 50% majority-rule consensus topologies. Posterior probabilities (PP) were calculated for all supported clades. A combined analysis of the three ribosomal genes was not undertaken because complete sequence data were not available for all species. Phylogenetic trees were visualised using FigTree v.1.4.4 [49].

## Results

**Fig. 1:**
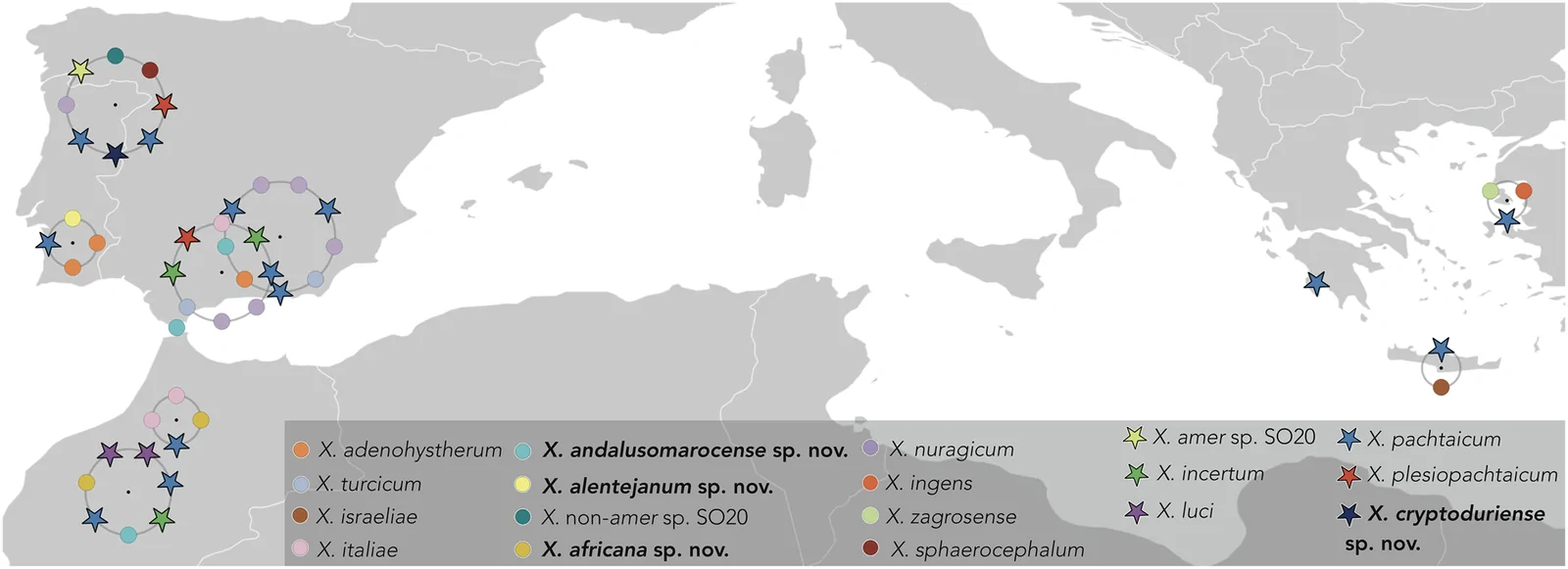
Geographic distribution of the sampling sites of olive groves in the Mediterranean Basin (black dots), and dagger nematode species of the genus *Xiphinema* detected in the present fieldwork. Dots and stars represent species belonging to *Xiphinema* non-*americanum* and *X. americanum* groups, respectively

From the 264 soil samples analysed, 50 populations of dagger nematode of the genus *Xiphinema* identified in this study occurred at low to moderately high soil densities (1–324 nematodes/500 cm^3^ of soil). All specimens were identified using an integrative taxonomic framework, and two previously unrecognised cryptic species complexes were revealed, one within the *X.* non-*americanum* group (*X. baetica* species complex) and one within the *X. americanum* group (*X. duriense* complex group). Within each complex, a new species is described herein, and two additional new species belonging to morphospecies Group 5 of the *X.* non-*americanum* group were also detected (Table 1, Fig. 1).

### Morphological variation between two previously unrecognised cryptic species complexes within the *X.* non-*americanum* group and *X. americanum* group

**Fig. 2:**
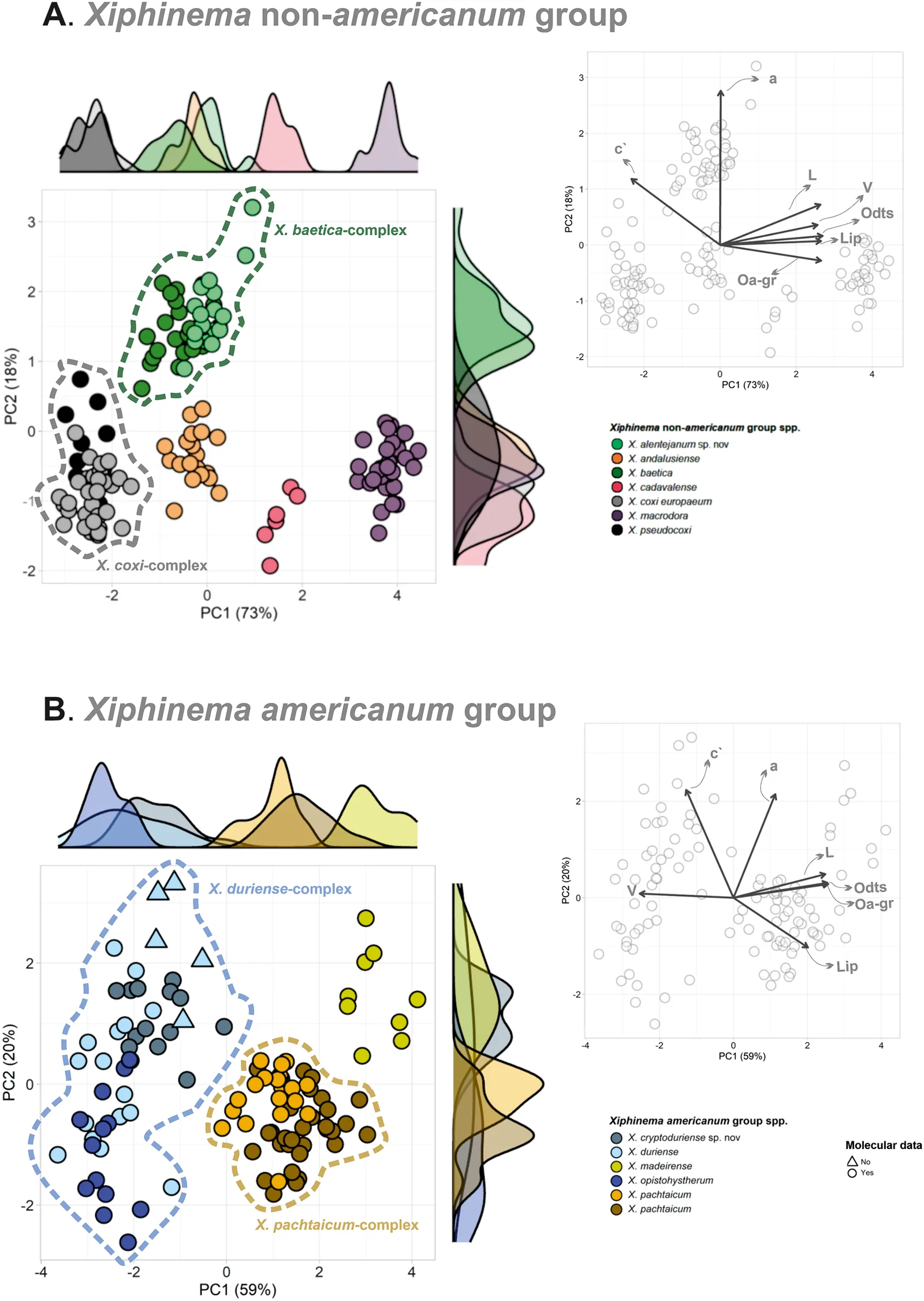
Multivariate morphometrics on the cryptic complexes in the *X.* non-*americanum* group and *X. americanum* group found in the Mediterranean olive-growing area. **A**, Results of the PCA performed on species found belonging to the *X.* non-*americanum* group. **B**, Results of the PCA performed on species found belonging to the *X. americanum* group. Abbreviations: a = Total body length/maximum body width; c’ = Tail length/body diameter at anus level; L = total body length; Lip = lip region width; Odts = odontostyle length; V = vulva position from the anterior body part; Oa-gr = distance between oral aperture to the guiding ring

The PCA provided a useful tool for characterising species limits within the *X.* non-*americanum-*group and *X. americanum-*group (Fig. 2). In both groups, we revealed two new cryptic complexes where specimens of each species were found overlapping one another, *X. baetica* species complex (i.e. the *X. alentejanum* sp. nov. and *X. baetica*), and *X. duriense*-complex (i.e. *X. cryptoduriense* sp. nov. *X. opisthohysterum*, and *X. duriense*), respectively. Specifically, for *X.* non-*americanum* group (Fig. 2A), multivariate morphometric analysis also showed the already known *X. coxi*-complex (only included sequenced populations: *X. coxi-europaeum* and *X. pseudocoxi*) [35]. However, we detected a clear separation amongst both cryptic complexes and with the other morphologically-related species included in this study (i.e. *X. andalusiense*, *X. cadavalense* and *X. macrodora*). This separation was primarily dominated by PC1, which accounted for most of the overall explained variation (73% of the 91% of variation explained; Fig. 2A). The variation of this axis was mainly dominated by total body and odontostyle length, lip region width and the distance between the oral aperture to the guiding ring (Fig. 2A). Therefore, we could refer to this as the size axis, supporting a previous study where distinctive cluster of species were identified with the size characteristics [38]. Considering the hypothesis that cryptic species would not differ in terms of size- but rather by shape-related characteristics [38], we here found that *X. baetica* species complex could be extended to include *X. andalusiense* due to the limited differences between them, which only differed in shape (note that PC2 was mainly dominated by the ratio a, but accounting for 18% of the 91% of overall variation explained; Fig. 2A). Our regression analyses supported this divergence pattern since LMM revealed significant differences between the species within *X. baetica* species complex and *X. andalusiense* along the shape variation (*p* < 0.01), while no effect on size variation (*p* = 0.81). However, we need to study this issue in further details. On the other hand, we found similar results in the multivariate morphometric analysis of the *X. americanum* group (Fig. 2B). First, we revealed a new cryptic complex within this group, *X. duriense*-complex formed by *X. cryptoduriense* sp. nov., *X. opistohystherum* and *X. duriense*. The fact that the cryptic complex already known as the *X. pachtaicum*-complex showed the same spatial distribution by overlapping of specimens from each species within supported the validity of the new cryptic complex identified in this study (Fig. 2B; note that only two species within this cryptic complex were included [8]. Second, we also obtained that PC1 was primarily dominated by size-related characteristics including the total body and odontostyle length, lip region width and the distance between the oral aperture to the guiding ring, and PC2 by shape-related characteristics such as the ratios c´and a (59 and 20% of the variation explained, respectively; Fig. 2B). Third, the spatial distribution in the graphic representation of the PCA in the *X. americanum* group and LMM results also showed that species within both cryptic complexes, *X. duriense*- and *X. pachtaicum*-complex, only significantly differed by shape- (*p* < 0.01 and *p* < 0.05), respectively but not in size-related characteristics (*p* = 0.69 and *p* = 0.06, respectively). Finally, Fig. 2B also revealed that the populations non-sequenced and belonging to *X. duriense* included in this analysis (including the type locality [27]); were also grouped within the *X. duriense*-complex.

### Species separation of *Xiphinema* non-*americanum* and *Xiphinema americanum*-group populations based on ribosomal and mitochondrial DNA

Molecular species-delineation analyses based on ribosomal and mitochondrial DNA confirmed that *Xiphinema alentejanum* sp. nov. and *Xiphinema cryptoduriense* sp. nov. are unequivocally distinct from all other nominal species within their respective complexes, *X. alentejanum* sp. nov. from *X. baetica*, *X. andalusiense*, and *X. macrodora*, and *X. cryptoduriense* sp. nov. from *X. duriense* and *X. opisthohysterum* (Table 2). The intraspecific/interspecific molecular variation for the D2–D3 expansion segments of 28S rRNA, ITS1, and COI genes of all species was < 0.10 for all species examined (Table 2), with the exception of D2–D3 of *X. baetica* (0.27), ITS1 of *X. baetica* (0.18), D2–D3 of *X. andalusiense* (0.14), ITS1 of *X. andalusiense* (0.10), and D2–D3 of *X. duriense* (0.31). These values indicate that, for most loci, the probability of correct species identification is moderately high [40]. Similarly, the PID (Liberal) values for all species and loci (ribosomal and mitochondrial) were ≥ 0.95 indicating that species were adequately delimited [41, 42], with the exception of the D2–D3 region of *X. baetica* (0.91), the COI locus of *X. andalusiense* (0.94), the D2–D3 region of *X. duriense* (0.83), and the D2–D3 region of *X. opisthohysterum* (0.94). Rosenberg probabilities were very low (*p ≤* 0.01) for both ribosomal and mitochondrial genes (thus rejecting the null hypothesis that observed monophyly results from random branching, for all pairwise species comparisons within the two complexes, except for the COI locus of *X. baetica* (0.33), *X. cryptoduriense* sp. nov. (0.33) and *X. duriense* (0.33). These results support the independent monophyly of each species [43] (Table 2).

**Table 2:** Parameters evaluating the species complexes *Xiphinema alentejanum* sp. nov. spp.-*Xiphinema baetica*, and *Xiphinema cryptoduriense* sp. nov.-*Xiphinema duriense*, with species delimitation based on two rRNA genes (D2–D3 expansion segments of the 28S rRNA, ITS) and one mtDNA barcoding locus, COI

Species	Gene	Closet species	**Intra/Inter**^**a**^	**P ID (Liberal)**^**b**^	**Rosenberg’s P**_**AB**_^**c**^
***Xiphinema *non*-americanum*-group**					
***Xiphinema alentejanum* sp. nov.**	D2–D3	*baetica*	0.08	**0.97 (0.87, 1.0)** ^**e**^	**0.004**
	ITS	*baetica*	0.03	**0.98 (0.87, 1.0)**	**0.004**
	COI	*baetica*	0.08	**0.97 (0.87, 1.0)**	**0.008**
*Xiphinema baetica*	D2–D3	*alentejanum* sp. nov.	0.27	**0.91 (0.80, 1.0)**	**0.004**
	ITS	*alentejanum* sp. nov.	0.18	0.87 (0.72, 1.0)	**0.008**
	COI	*alentejanum* sp. nov.	0.02	**0.97 (0.82, 1.0)**	0.33
*Xiphinema andalusiense*	D2–D3	*baetica*	0.14	**0.95 (0.84, 1.0)**	**0.001**
	ITS	*baetica*	0.10	**0.97 (0.87, 1.0)**	**0.007**
	COI	*alentejanum* sp. nov.	0.06	**0.94 (0.79, 1.0)**	**0.005**
*Xiphinema macrodora*	D2–D3	*andalusiense*	0.06	**0.97 (0.86, 1.0)**	**0.002**
	ITS	*baetica*	0.01	**0.98 (0.83, 1.0)**	**0.009**
	COI	-	-	-	-
***Xiphinema americanum-*group**					
***Xiphinema cryptoduriense* sp. nov.**	D2–D3	*duriense*	0.11	**0.97 (0.87, 1.0)**	**0.003**
	ITS	-	-	-	-
	COI	*duriense*	0.05	**0.95 (0.80, 1.0)**	0.33
*Xiphinema duriense*	D2–D3	***cryptoduriense *sp. nov.**	0.31	**0.83 (0.69, 1.0)**	**0.003**
	ITS	-	-	-	-
	COI	***cryptoduriense* sp. nov.**	0.00	**0.96 (0.83, 1.0)**	0.33
*Xiphinema opisthohysterum*	D2–D3	***cryptoduriense* sp. nov.**	0.08	**0.94 (0.78, 1.0)**	**0.004**
	ITS	-	-	-	-
	COI	***cryptoduriense* sp. nov.**	0.04	**0.96 (0.81, 1.0)**	**0.005**

^a^Intraspecific variation relative to interspecific variation. ^b^ The P ID (Liberal) value represents the probability (with the 95% confidence interval) for the prediction of making a correct identification of an unknown specimen of the focal species using DNA Barcoding (closest genetic distance). P ID (Liberal) values ≥ 0.93 were considered to be significant delimited [42]. Numbers in bold represent significant values. ^c^ Rosenberg’s P_AB_ value is the probability that the monophyly of a group of sequences is the result of random branching, Rosenberg’s significant values = *p* < 0.01. ^e^ Significant results are indicated in bold. (-) Not obtained or not performed because none or only a single sequence of D2–D3 or ITS for this species is available in NCBI

### Phylogenetic relationships among *xiphinema* non-*americanum* and *xiphinema americanum-*group species within *xiphinema*

Amplification of the D2–D3 expansion segments of 28S rRNA, ITS1 rRNA, partial 18S rRNA and COI yielded single fragments of approximately 800 bp, 1000 bp, 1800 bp and 400 bp, respectively, as confirmed by gel electrophoresis. Sequences from other *Xiphinema* spp. available in the National Center for Biotechnology Information database (http://www.ncbi.nlm.nih.gov/) were incorporated into the phylogenetic analyses. Novel sequences were generated for *X. andalusomarocense* sp. nov., *X. africana* sp. nov., *X. alentejanum* sp. nov., and *X. cryptoduriense* sp. nov. and *Xiphinema* non-*americanum* sp. SO20. In contrast, sequences obtained for *X. adenohysterum*,, *X. incertum, X. ingens*, *X. italiae*, *X. luci*, *X. nuragicum*, *X. pachtaicum*, *X. plesiopachtaicum*, *X. sphaerocephalum* and *X. zagrosense* matched previously deposited GenBank accessions, thereby extending the known molecular diversity and geographical ranges of these species.

**Fig. 3:**
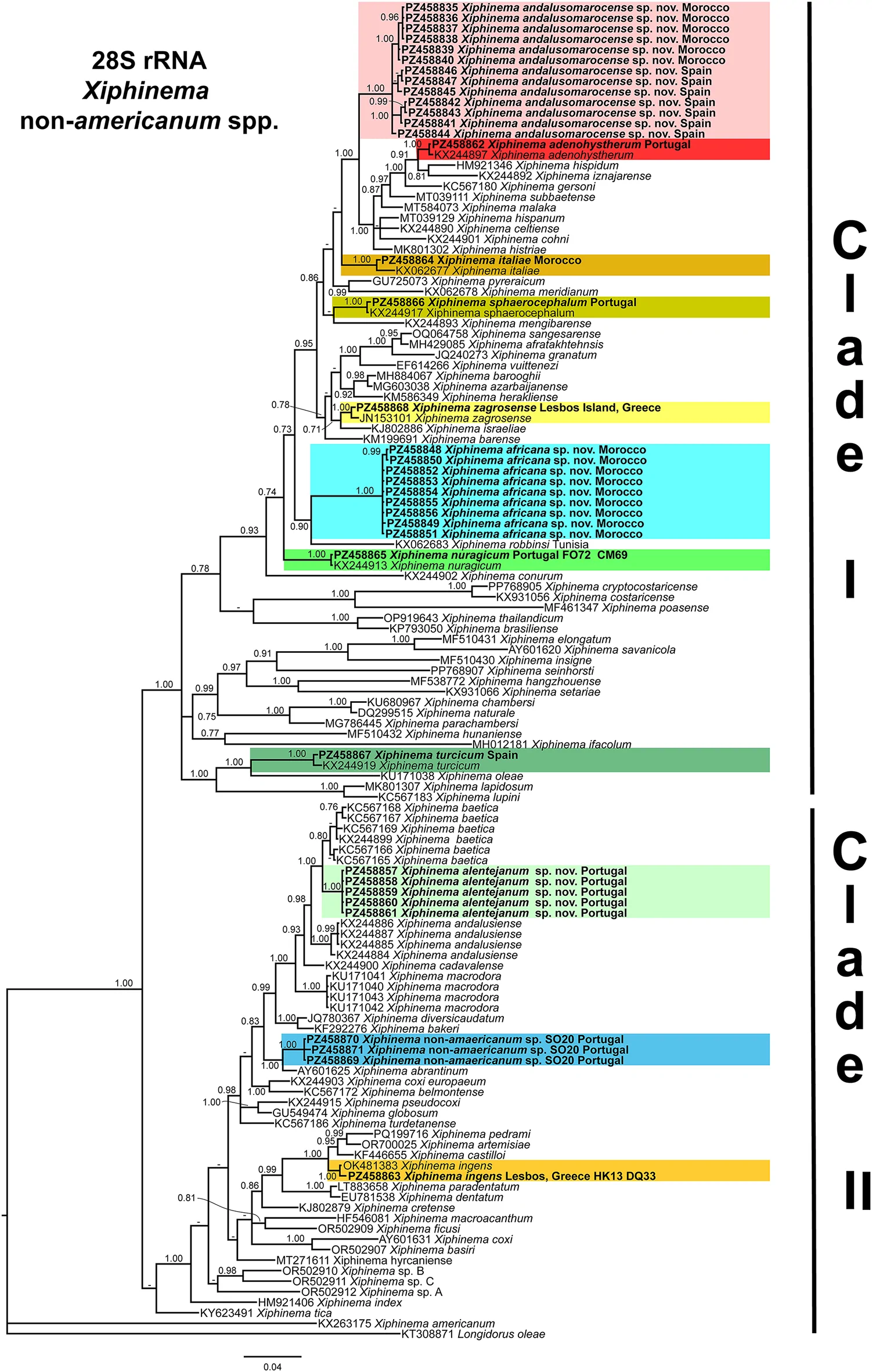
Phylogenetic relationships of *Xiphinema* non-*americanum*-group species from olive groves and adjacent natural vegetation in the Mediterranean Basin. Bayesian 50% majority-rule consensus tree inferred from D2–D3 expansion segments of 28S rRNA sequence alignment under the GTR + I+ G model (−lnl = 11695.0651; AIC = 23914.130160; freqA = 0.2315; freqC = 0.2165; freqG = 0.3123; freqT = 0.2397; R(a) = 1.2493; R(b) = 3.2587; R(c) = 3.0069; R(d) = 0.5989; R(e) = 5.1919; R(f) = 1.0000; Pinva = 0.3620; and Shape = 0.6860). Posterior probabilities ≥ 0.70 are shown at the corresponding nodes. Newly generated sequences are indicated in bold, and coloured boxes denote the clade placement of new sequenced species. Scale bar: expected subtitutions per site

**Fig. 4:**
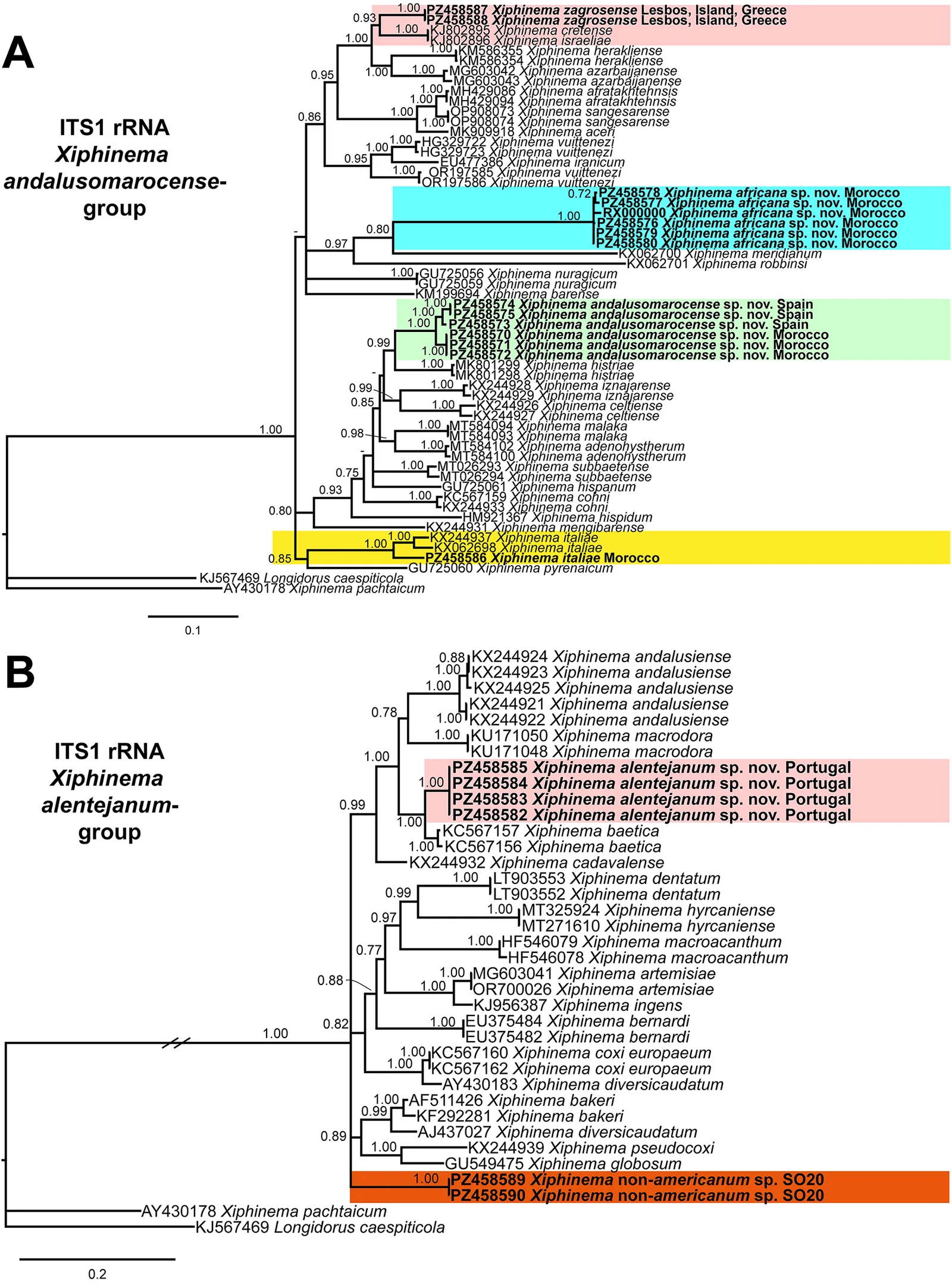
Phylogenetic relationships of *Xiphinema* non-*americanum*-group species from olive groves and adjacent natural vegetation in the Mediterranean Basin. **A**) *Xiphinema andalusomarocense* sp. nov. And *Xiphinema africana* sp. nov. group; **B**) *Xiphinema alentejanum* sp. nov. group. Bayesian 50% majority-rule consensus tree inferred from ITS rRNA sequence alignment under the models: **A**) TRN + I+ G (−lnl = 14070.0569; AIC = 28382.113720; freqA = 0.3032; freqC = 0.1796; freqG = 0.2430; freqT = 0.2742; R(a) = 1.0000; R(b) = 2.5155; R(c) = 1.0000; R(d) = 1.0000; R(e) = 4.0359; R(f) = 1.0000; Pinva = 0.0990; and Shape = 0.7650); **B**) GTR + I+ G (−lnl = 8455.7818; AIC = 17067.563660; freqA = 0.2871; freqC = 0.1881; freqG = 0.2562; freqT = 0.2686; R(a) = 0.6655; R(b) = 2.3526; R(c) = 1.5087; R(d) = 0.6812; R(e) = 3.4228; R(f) = 1.0000; Pinva = 0.0000; and Shape = 0.5620. Posterior probabilities ≥ 0.70 are shown at the corresponding nodes. Newly generated sequences are indicated in bold, and coloured boxes denote the clade placement of new sequenced species. Scale bar: expected subtitutions per site

**Fig. 5:**
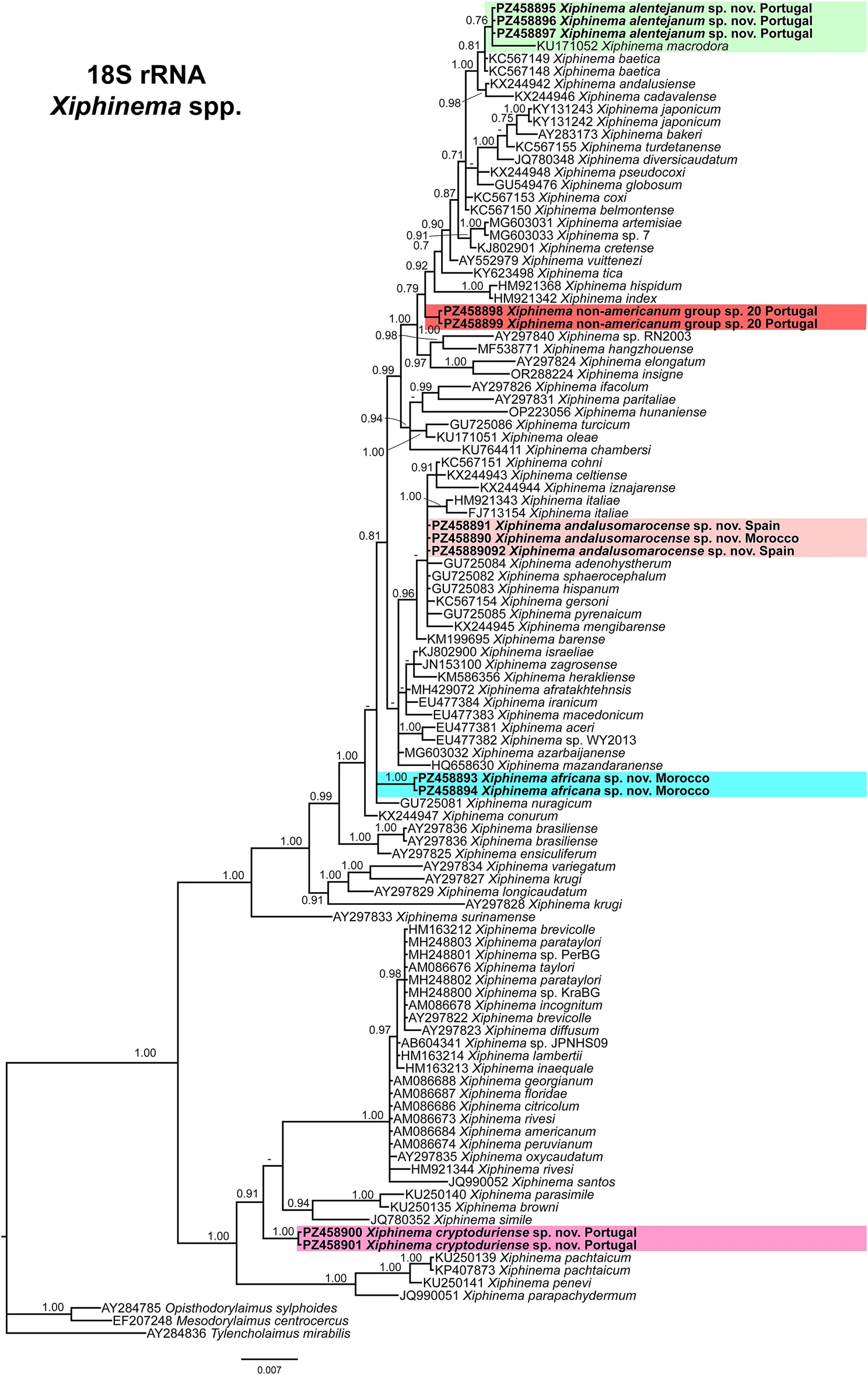
Phylogenetic relationships of *Xiphinema* non-*americanum*-group and *Xiphinema americanum*-group species from olive groves and adjacent natural vegetation in the Mediterranean Basin. Bayesian 50% majority-rule consensus tree inferred from 18S rRNA sequence alignment under the SYM + I+ G model (−lnl = 6113.4579; AIC = 12668.915740; freqA = 0.2500; freqC = 0.2500; freqG = 0.2500; freqT = 0.2500; R(a) = 0.9167; R(b) = 2.5939; R(c) = 1.2150; R(d) = 0.3231; R(e) = 4.8748; R(f) = 1.0000; Pinva = 0.7210; and Shape = 0.6850). Posterior probabilities ≥ 0.70 are shown at the corresponding nodes. Newly generated sequences are indicated in bold, and coloured boxes denote the clade placement of new sequenced species. Scale bar: expected subtitutions per site

**Fig. 6:**
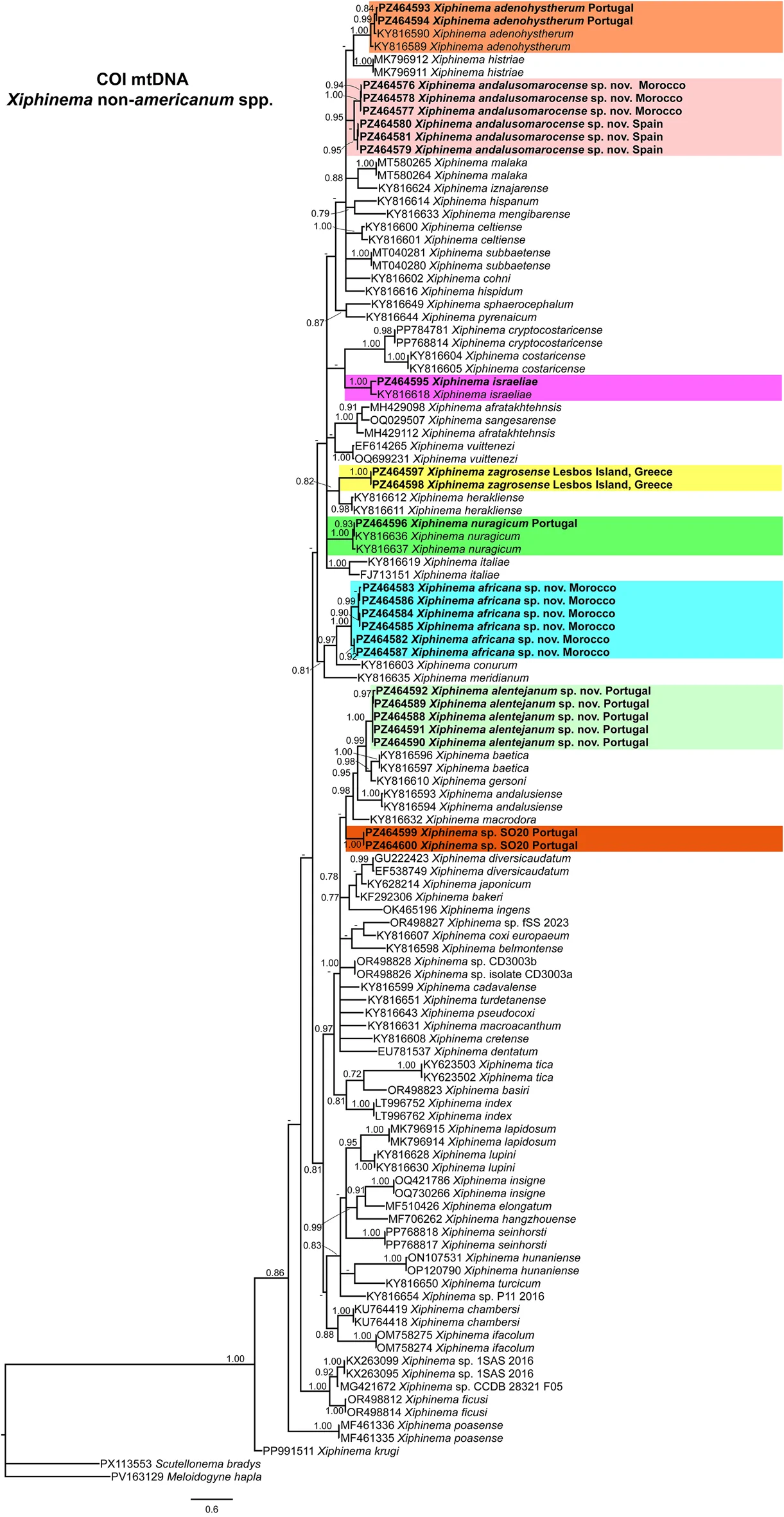
Phylogenetic relationships of *Xiphinema* non-*americanum*-group species from olive groves and adjacent natural vegetation in the Mediterranean Basin. Bayesian 50% majority-rule consensus tree inferred from cytochrome c oxidase subunit I (COI) mtDNA gene sequence alignment under the TVM + I+ G model (−lnl = 11959.4252; AIC = 24392.850380; freqA = 0.3283; freqC = 0.1505; freqG = 0.1208; freqT = 0.4005; R(a) = 1.1303; R(b) = 13.2203; R(c) = 0.5118; R(d) = 2.0420; R(e) = 13.2203; R(f) = 1.0000; Pinva = 0.3290; and Shape = 0.3950). Posterior probabilities ≥ 0.70 are shown at the corresponding nodes. Newly generated sequences are indicated in bold, and coloured boxes denote the clade placement of new sequenced species. Scale bar: expected subtitutions per site

Phylogenetic relationships among *X.* non-*americanum* group species inferred from Bayesian analyses of the D2–D3 expansion segments of 28S rRNA, ITS1, partial 18S rDNA, and COI gene sequences are presented in Figs. 3, 4, 5 and 6. Poorly supported clusters were not explicitly labelled. The 50% majority-rule consensus tree of 28S rRNA dataset, based on a manually curated alignment of 121 sequences and 769 characters, revealed two well-defined major clades (PP = 1.00) (Fig. 3). Clade I, although not strongly supported overall, comprised 50 species, including two new species described here, *X. andalusomarocense* sp. nov. and *X. africana* sp. nov., as well as all first records identified in this study. *Xiphinema andalusomarocense* sp. nov. formed a strongly supported subclade (PP = 1.00) with *X. adenohystherum* (KX244897), *X. hispidum* (HM921346), *X. iznajarense* (KX244892), *X. gersoni* (KC567180), *X. subbaetense* (MT039111), *X. malaka* (MT584073), *X. hispanum* (MT039129), *X. celtiense* (KX244890), *X. cohni* (KX244901), and *X. histriae* (MK801302). *Xiphinema africana* sp. nov. was recovered as a sister to *X. robbinsi* (KX062683) from Tunisia; although, the broader placement of this lineage remains uncertain due to weak support (PP = 0.73). First records identified in this study include *X. adenohystherum* (PZ458862), *X. nuragicum* (PZ458865) and *X. sphaerocephalum* (PZ458866) from Portugal; *X. italiae* (PZ458864) from Morocco; and *X. zagrosense* (PZ458868) from Greece. All matched well with existing GenBank accessions. Clade II, also weakly supported, comprised 27 species and included sequences of *X. alentejanum* sp. nov. (PZ458857–PZ458861), *X.* non-*americanum* sp. SO20, and the first Greek record of *X. ingens* (PZ458863). *X. alentejanum* sp. nov. (PZ458857–PZ458861) clustered with *X. baetica* (KC567165–KC567169), *X. andalusiense* (KX244884–KX244888), *X. cadavalense* (KX244900) and *X. macrodora* (KU171040–KU171044), forming a strongly supported subclade (PP = 1.00) (Fig. 4). *X.* non-*americanum* sp. SO20 (PZ458869–PZ458871) grouped with *X. abrantinum* (AY601625), while *X. ingens* (PZ458863) occupied a basal position within the clade, clustering with the Russian accession of *X. ingens* (OK481383).

Difficulties were encountered when aligning the ITS1 sequences of *X.* non-*americanum* group species due to their low similarity with available NCBI accessions. Consequently, two separate phylogenetic analyses were performed, each including only the species most closely related to our material (Fig. 4). The first tree comprised *X. africana* sp. nov., *X. andalusomarocense* sp. nov., *X. zagrosense*, and *X. italiae* (Fig. 4A), whereas the second included only *X. alentejanum* sp. nov. and *X.* non-*americanum* sp. SO20 (Fig. 4B). After removing ambiguously aligned regions, the final ITS1 alignments contained 1113 and 1036 characters derived from 37 and 58 sequences, respectively. The placement of the new species in the ITS1 phylogenies was broadly congruent with the 28S rRNA results (Fig. 3). *Xiphinema africana* sp. nov. (PZ458876–PZ458881) clustered with *X. meridianum* (KX062700) and *X. robbinsi* from Tunisia (KX062701) in a well-supported clade (PP = 0.97), although, deeper relationships within the group remained unresolved. The six ITS1 accesions of *X. andalusomarocense* sp. nov. formed a strongly supported subclade (PP = 0.99) with *X. histriae*, nested within a larger clade including *X. adenohystherum*, *X. hispidum*, *X. iznajarense*, *X. subbaetense*, *X. malaka*, *X. hispanum*, *X. celtiense* and *X. cohni*. A similar situation was observed for *X. alentejanum* sp. nov., which clustered with *X. andalusiense* (KX244923–KX244925), *X. baetica* (KC567156- KC567157), *X. macrodroa* (KU171048, KU171050) and *X. cadavalense* (KX244932) in a highly supported clade (PP = 1.00). Finally, *X.* non-*americanum* sp. SO20 (PZ458869–PZ458871) occupied a basal position in the ITS1 tree and did not cluster with any other *Xiphinema* spp. accession.

Due to the low variability and high sequence homology of the 18S rRNA region, a single phylogeny was inferred by both the *X.* non‑*americanum* group and *X. americanum* group. The 50% majority-rule BI tree, based on a 1647 bp alignment of 108 sequences, recovered two major clades (Fig. 5): one comprising accessions from *X.* non-*americanum* group and a second containing members of the *X.* a*mericanum* group. In the D2–D3, ITS1 and COI (see below) phylogenies, *X. alentejanum* sp. nov. consistently clustered with *X. andalusiense* (KX244942), *X. cadavalense* (KX244946), *X. macrodora* (KU171052) and *X. baetica* (KC567148–KC567149) in a strongly supported subclade (PP = 1.00). In contrast, the 18S rRNA phylogeny did not resolve the relationships of *X.* non-*americanum* sp. SO20 (PZ458898–PZ458899) or *X. andalusomarocense* sp. nov., reflecting the limited discriminatory power of this marker at shallow taxonomic levels. *Xiphinema africana* sp. nov. (PZ458893–PZ458894) formed a distinct, well-supported clade (PP = 1.00). The 18S rRNA analysis also placed *X. cryptoduriense* sp. nov. (PZ458898–PZ458899) with *X. pachtaicum* (KU250139–KP407873), *X. penevi* (KU250141) and *X. parapachydermum* (JQ990051), in a highly supported clade (PP = 1.00), but this species was related with many other species from the *X. americanum*-group species complex with a moderate support (0.91). Unfortunately, partial 18S rRNA sequences for *X. duriense* and *X. opisthohysterum* are not yet available in GenBank, preventing a more complete assessment of their phylogenetic placement.

Phylogenetic analyses (BI) of the *X.* non-*americanum* group based on partial COI sequences (115 sequences, 405 bp) recovered several clades with low or no support, except for a few deeper relationships within the tree (Fig. 6). Despite this limited resolution, the COI marker maintained the association of *X. alentejanum* sp. nov. (PZ464589–PZ464592) with *X. baetica* (KY816596–KY816597), *X. andalusiense* (KY816593–KY816594), and *X. macrodora* (KY816632). A similar pattern was observed for *X. africana* sp. nov. (PZ464582–PZ464587), which formed a well‑supported subclade with *X. conurum* (KY816603) and *X. meridianum* (KY816635). In contrast, *X. andalusomarocense* sp. nov. (PZ464576–PZ464581) did not cluster with any other *Xiphinema* species when COI was used. *Xiphinema* non-*americanum* group sp. SO20 (PZ464599–PZ464600) appeared within the *X. alentejanum* sp. nov. subclade, although with weak support, reflecting the limited resolving power of COI for some shallow relationships. The first records identified in this study: *X. adenohystherum* (PZ464593–PZ464594) and *X. nuragicum* (PZ464596) from Portugal; *X. zagrosense* (PZ464597–PZ464598) from Greece, matched well with existing GenBank accessions, confirming their molecular identity.

**Fig. 7:**
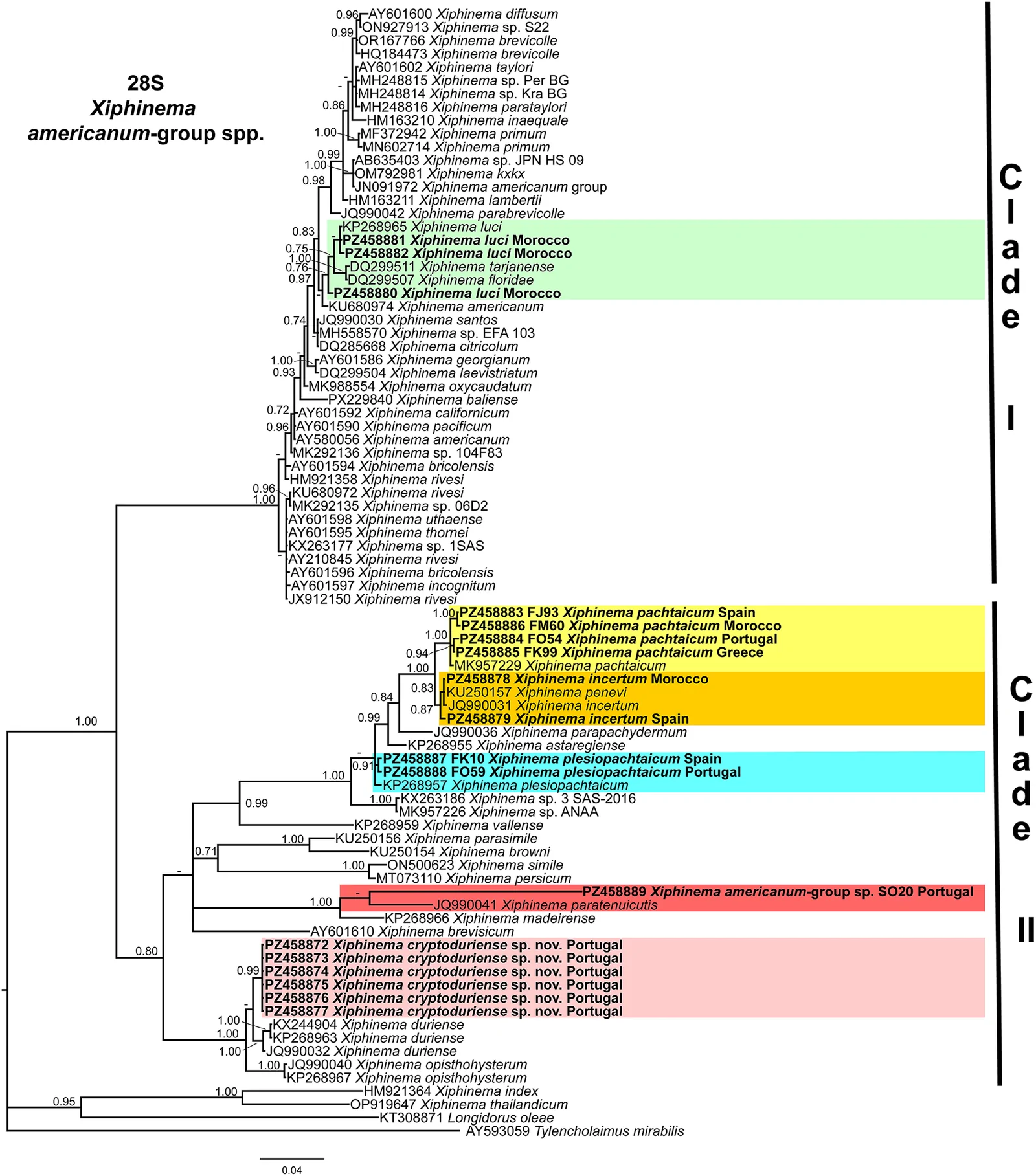
Phylogenetic relationships of *Xiphinema americanum*-group species from olive groves and adjacent natural vegetation in the Mediterranean Basin. Bayesian 50% majority-rule consensus tree inferred from D2–D3 expansion segments of 28S rRNA sequence alignment under the GTR + G model (−lnl = 6641.2438; AIC = 13636.487620; freqA = 0.2259; freqC = 0.2601; freqG = 0.3103; freqT = 0.2036; R(a) = 0.7778; R(b) = 2.8103; R(c) = 2.9887; R(d) = 0.4434; R(e) = 5.5267; R(f) = 1.0000; Pinva = 0.0000; and Shape = 0.3920). Posterior probabilities ≥ 0.70 are shown at the corresponding nodes. Newly generated sequences are indicated in bold, and coloured boxes denote the clade placement of new sequenced species. Scale bar: expected subtitutions per site

The 50% majority-rule consensus BI tree for the 28S rRNA gene of the *X. americanum* group species was generated from a manually curated alignment comprising 85 sequences and 737 characters (Fig. 7). The phylogeny revealed two well-supported and clearly differentiated clades (PP = 1.00). The first record of *X. luci* from Morocco (PZ458880–PZ458882) clustered with *X. luci* (KP268965) from Spain, together with *X. tarjanense* and *X. floridae*. In contrast, the second clade displayed markedly longer branches, forming a more heterogeneous and evolutionarily divergent assemblage. *Xiphinema cryptoduriense* sp. nov. fell within clade II, which corresponds to the *pachtaicum*-subgroup *sensu* Lamberti & Ciancio [50, 51]. *Xiphinema cryptoduriense* sp. nov. clustered with *X. duriense* (KX244904, KP268963, JQ990032) and *X. opisthohysterum* (JQ990040, KP268967) in a strongly supported subclade (PP = 1.00). *Xiphinema americanum* group sp. SO20 (PZ458889) was phylogenetically related to *X. paratenuicutis* (JQ990041), although relationships among the remaining species were poorly resolved, with several subclades receiving weak support (Fig. 7). The new accessions of *X. pachtaicum* (PZ458883–PZ458886), *X. incertum* (PZ458878–PZ458879), and *X. plesiopachtaicum* (PZ458887–PZ458888) formed a well-supported clade (PP = 1.00) together with *X. penevi* (KU250157), *X. parapachydermum* (JQ990036), *X. astaregiense* (KP268955), *Xiphinema* sp. 3 SAS (KX263186), and *Xiphinema* sp. ANNA (MK957226).

Although an ITS1 sequence was successfully generated in this study for newly obtained *X. americanum*-group populations, this marker was not included in the phylogenetic analyses. Available ITS1 sequences in GenBank for species of the *X. americanum* group show extremely low homology with our material, resulting in high divergence and preventing the construction of reliable multiple-sequence alignments. Including such heterogeneous sequences would likely produce unstable or artefactual topologies; therefore, ITS1 phylogeny of the *X. americanum* group was omitted due to insufficient comparability with existing datasets.

**Fig. 8:**
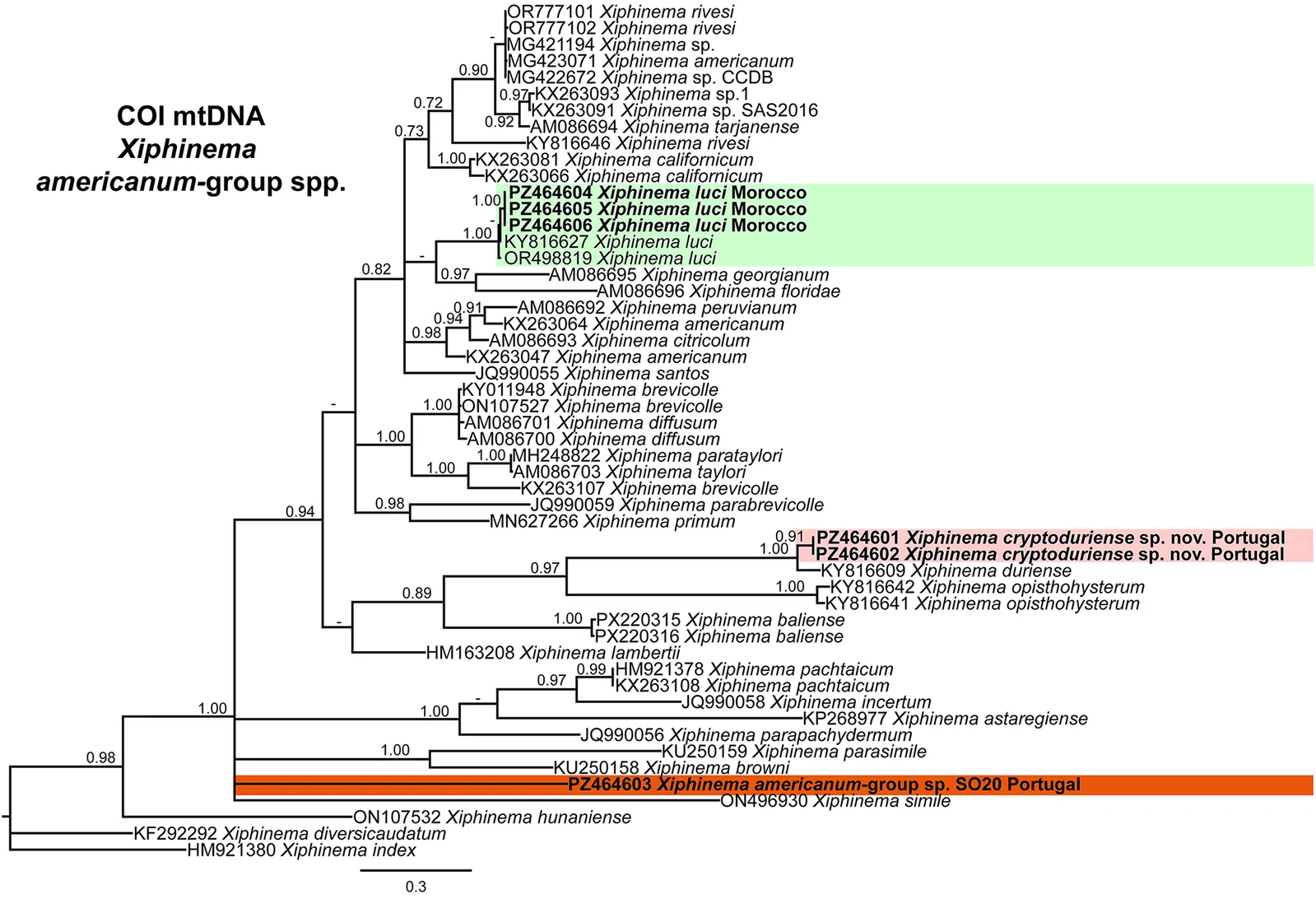
Phylogenetic relationships of *Xiphinema americanum*-group species from olive groves and adjacent natural vegetation in the Mediterranean Basin. Bayesian 50% majority-rule consensus tree inferred from cytochrome c oxidase subunit I (COI) mtDNA gene sequence alignment under the TPM1uf + I + G model (−lnl = 5900.7014; AIC = 12019.402720; freqA = 0.3202; freqC = 0.1384; freqG = 0.1457; freqT = 0.3957; R(a) = 1.0000; R(b) = 14.0611; R(c) = 0.3486; R(d) = 0.3486; R(e) = 14.0611; R(f) = 1.0000; Pinva = 0.3470; and Shape = 0.4310). Posterior probabilities ≥ 0.70 are shown at the corresponding nodes. Newly generated sequences are indicated in bold, and coloured boxes denote the clade placement of new sequenced species. Scale bar: expected subtitutions per site

The BI phylogeny based on partial COI (51 sequences, 383 positions) recovered a major clade containing most species of the *X. americanum* group (Fig. 8). Within this clade, *X. cryptoduriense* sp. nov. (PZ464601–PZ464602) clustered with *X. duriense* (KY816609) and *X. opisthohysterum* (KY816641–KY816642) in a well-supported subclade (PP = 0.97). In contrast, *X. americanum* group sp. SO20 (PZ464603) occupied an isolated position and did not cluster within any well-supported lineage.

### Morphology, morphometrics and molecular characterisation of *Xiphinema* non-americanum-group species

All *Xiphinema* spp. identified in this study, including specimens from sampling sites used for morphological and/or molecular analyses, are listed in Table 1. In addition, all positive records of *Xiphinema* spp. and their corresponding sampling sites are shown in Fig. 1. In total, 50 *Xiphinema* populations were detected across 264 sampling sites in the rhizosphere of olive trees and adjacent patches of natural vegetation. These populations represent 18 *Xiphinema* species: 12 belonging to the *X*. non-*americanum* group, three of which are described here as new species, and six belonging to the *X*. *americanum* group, one of which is described here as a new species (Table 1).

**Fig. 9:**
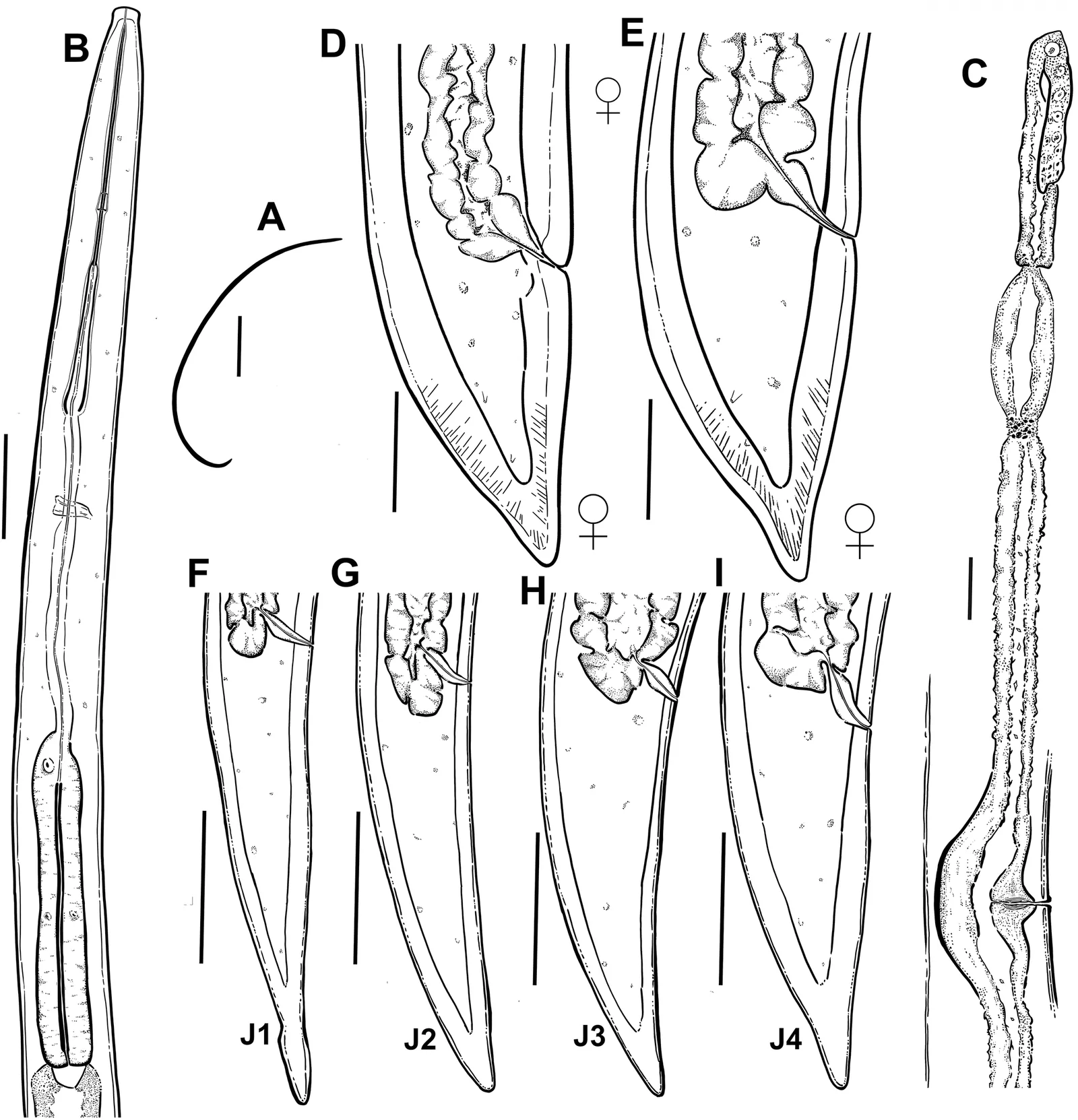
Line drawings of *Xiphinema andalusomarocense* sp. nov. **A**, entire female; **B**, female neck region; **C**, detail of anterior genital branch and vulval region; **D**, **E**, female tail region; **F**-**I**, first-, second-, third-, and fourth-stage juvenile tails (**J1**–**J4**), respectively. Scale bars: A = 500 μm; B = 50 μm; C = 20 μm; D-I = 20 μm

**Fig. 10:**
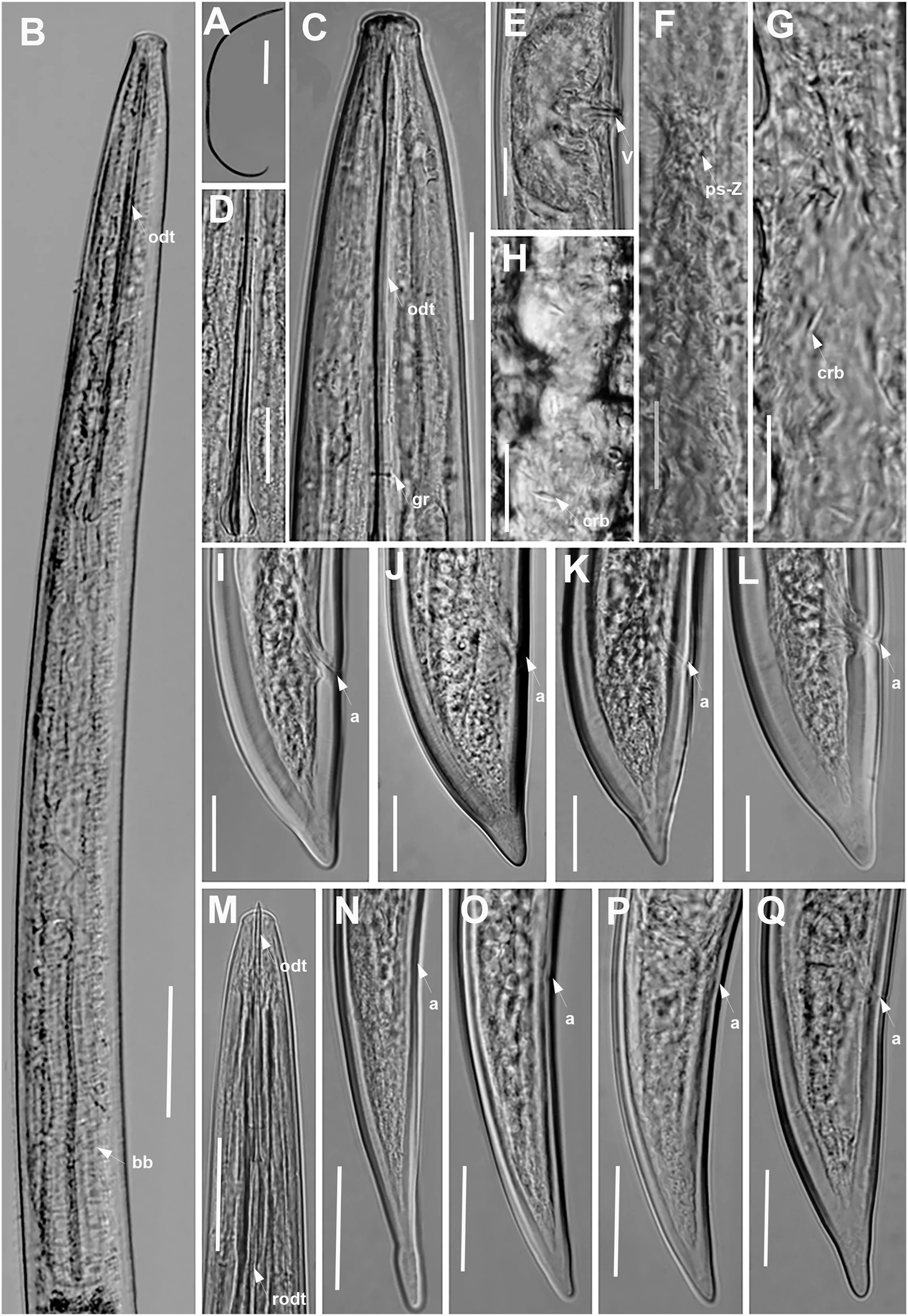
Light micrographs of *Xiphinema andalusomarocense* sp. nov. **A**, entire female; **B**, female neck region; **C**, female anterior region; **D**, detail of odontophore; **E**, vulval region; **F**–**H**, detail of pseudo-Z organ and small crystalloid bodies and spiniform structures; **I**–**L**, female tail region; **M**, first-stage juvenile anterior region; **N**–**Q**, first-, second-, third-, and fourth-stage juvenile tails (J1–J4), respectively. Abbreviations: a = anus; bb = basal bulb; crb = crystallod body; gr = guiding ring; odt = odontostyle; ps-Z = pseudo-Z organ; rodt = replacement odontostyle; V = vulva. Scale bars: A = 500 μm; B = 50 μm; C-*P* = 20 μm

**Fig. 11:**
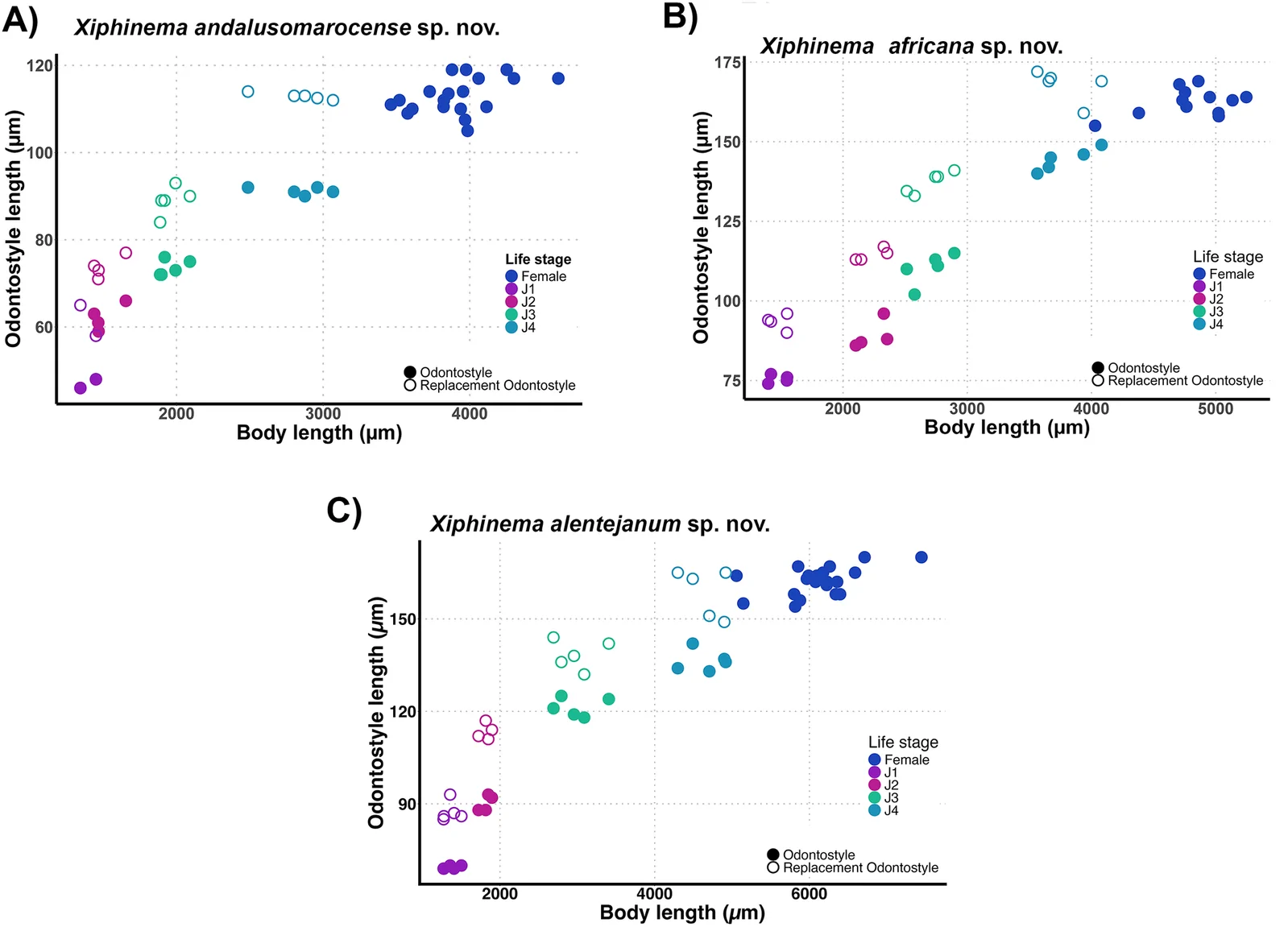
Relationship of body length to length of functional and replacement odontostyle length in all developmental stages from first-stage juveniles (J1) to mature females of: **A**, *Xiphinema andalusomarocense* sp. nov.; **B**, *Xiphinema africana* sp. nov.; **C**, *Xiphinema alentejanum* sp. nov.

***Xiphinema andalusomarocense*** **sp. nov.** (Figs. 9, 10 and 11; Table 3)

**Table 3:** Morphometrics of *Xiphinema andalusomarocense* sp. nov. holotype and paratypes from olive (*Olea europaea* subspp. *europaea*) from Ait Taleb, Azilal province, Morocco; and two localities from southern Spain. All measurements are in µm and in the form: mean ± s.d. (range)

Character/ratio^1^	Holotype	Paratypes					Chiclana de Segura, Jaén, Spain	Tarifa, Cádiz, Spain
		Females	J1	J2	J3	J4	Females	Female
n		20	2	4	5	5	8	1
L	3938	3901 ± 0.284	(1344, 1450)	1507 ± 0.99	1958 ± 85	2838 ± 220	3925 ± 351	3.683
		(3462–4604)		(1438–1654)	(1880–2091)	(2487–3067)	(3409–4427)	
a	88.5	92.6 ± 5.9	(53.8, 60.4)	56.9 ± 2.9	65.0 ± 4.9	76.0 ± 12.2	91.4 ± 11.8	90.9
		(84.9–107.4)		(53.8–59.9)	(58.4–69.9)	(59.9–93.4)	(84.9–107.4)	
b	9.4	9.7 ± 0.9	(5.9, 6.1)	5.7 ± 0.3	7.2 ± 0.7	9.0 ± 1.0	10.8 ± 0.7	8.2
		(8.1–11.4)		(5.4–6.1)	(6.4–8.0)	(7.8–10.1)	(9.6–11.4)	
c	73.5	77.8 ± 6.5	(22.0, 24.6)	24.1 ± 2.1	32.7 ± 0.9	47.9 ± 2.7	82.2 ± 6.9	87.8
		(66.5–98.0)		(22.4–26.7)	(31.6–33.7)	(43.6–50.3)	(70.3–88.5)	
c’	1.8	1.7 ± 0.1	(3.1, 3.3)	3.0 ± 0.2	3.0 ± 0.1	2.3 ± 0.2	1.7 ± 0.2	1.5
		(1.5–2.0)		(2.8–3.2)	(2.8–3.1)	(2.0–2.5)	(1.5–1.9)	
d	8.0	7.5 ± 0.5	(5.8, 6.2)	6.1 ± 0.1	7.2 ± 0.5	6.8 ± 0.5	7.4 ± 0.8	6.9
		(6.5–8.6)		(6.0–6.2)	(6.5–7.9)	(6.0–7.2)	(5.8–8.0)	
d’	2.4	2.4 ± 0.2	(2.2, 2.5)	2.3 ± 0.1	2.2 ± 0.2	2.3 ± 0.1	2.3 ± 0.2	2.1
		(2.2–2.7)		(2.1–2.3)	(2.0–2.4)	(2.2–2.5)	(2.1–2.6)	
V	48.5	48.2 ± 1.1	-	-	-	-	47.6 ± 1.4	49.5
		(46.1–49.9)					(46.0–49.4)	
G_1_	9.4	9.7 ± 0.9	-	-	-	-	9.5 ± 0.6	8.0
		(8.4–11.5)					(8.8–10.1)	
G_2_	9.3	9.1 ± 0.8	-	-	-	-	8.9 ± 0.7	7.5
		(7.8–10.7)					(8.1–9.9)	
Odontostyle	110.0	112.9 ± 4.1	(46.0, 48.0)	62.3 ± 3.0	73.6 ± 1.8	91.2 ± 0.8	104.9 ± 1.7	98.5
		(105.0–119.0)		(59.0–66.0)	(72.0–76.0)	(90.0–92.0)	(103.0–108.0)	
Odontophore	69.0	68.7 ± 3.4	(43.0, 46.5)	48.8 ± 5.2	55.8 ± 2.8	65.0 ± 4.5	57.6 ± 5.3	61.5
		(62.0–75.0)		(45.0–56.0)	(53.0–60.0)	(62.0–72.0)	(47.0–63.0)	
Total stylet	179.0	181.0 ± 16.8	(91.0, 92.5)	111.0 ± 7.7	129.4 ± 3.8	156.2 ± 4.9	162.6 ± 5.6	160
		(166.0–194.0)		(104.0–122.0)	(125.0–135.0)	(152.0–164.0)	(151.0–170.0)	
Replacement odontostyle	-	-	(58.0, 65.0)	73.8 ± 2.5	89.0 ± 3.2	112.9 ± 0.7	-	-
				(71.0–77.0)	(84.0–93.0)	(112.0–114.0)		
Lip region diam.	12.5	12.8 ± 0.4	(9.0, 10.0)	9.3 ± 0.5	9.8 ± 0.4	11.8 ± 0.4	12.5 ± 0.3	13.0
		(12.0–13.5)		(9.0–10.0)	(9.0–10.0)	(11.0–12.0)	(12.0–13.0)	
Oral aperture to guiding ring	100.0	96.4 ± 5.7	(56.0, 62.0)	56.0 ± 2.8	70.3 ± 3.2	79.8 ± 5.1	92.1 ± 7.5	90.0
		(81.0–108.0)		(54.0–60.0)	(65.0–73.0)	(72.0–85.0)	(75.0–100.0)	
Max. body diam.	44.5	42.2 ± 3.0	(24.0, 25.0)	26.0 ± 2.7	30.3 ± 2.9	37.9 ± 4.9	45.3 ± 6.5	40.5
		(37.0–46.0)		(24.0–30.0)	(27.0–34.0)	(30.0–42.0)	(37.0–53.0)	
Tail length	53.5	50.3 ± 2.9	(59.0, 61.0)	61.3 ± 2.2	59.9 ± 2.4	59.2 ± 1.9	50.9 ± 4.5	42.0
		(45.5–56.0)		(59.0–64.0)	(57.0–62.5)	(57.0–61.0)	(45.0–57.0)	
J	14.5	13.9 ± 1.6	(12.0, 14.0)	10.8 ± 1.0	11.5 ± 0.5	13.6 ± 2.0	11.1 ± 1.5	13.0
		(11.5–17.0)		(10.0–12.0)	(11.0–12.0)	(11.5–17.0)	(10.0–14.0)	

^1^Abbreviations as defined: a, body length/maximum body width; b, body length/pharyngeal length; c, body length/tail length; c’, tail length/body width at anus; V (distance from anterior end to vulva/body length) ×100; d = anterior to guiding ring/body diam. at lip region; d’ = body diam. at guiding ring/body diam. at lip region; J = hyaline tail region length

#### ZooBank

**urn:** lsid:zoobank.org:act: 8B70C0A4-B92A-4F44-B373-963656B4E6C5

#### Holotype

Female extracted from a soil sample collected from the rhizosphere of cultivated olive (*Olea europaea* ssp. *europaea* L.) Ait Taleb, Azilal, Morocco (32.147157 N latitude, −6.649693 W longitude), by A. Bajoub, mounted in pure glycerine and deposited in the Nematode collection of the Laboratorio de Fitonematología, Institute for Sustainable Agriculture, CSIC, Spain (slide number SO36_6_3).

#### Paratypes

Females and juvenile stages collected from the rhizosphere of cultivated olive (*Olea europaea* ssp. *europaea* L.) Ait Taleb, Azilal, Morocco (32.147157 N latitude, −6.649693 W longitude),by A. Bajoub, mounted in pure glycerine and deposited in the Nematode collection of the Laboratorio de Fitonematología, Institute for Sustainable Agriculture, CSIC, Spain (slide number SO36_6_5-18); and two female paratypes at the USDA Nematode Collection, Beltsville, MD, USA (collection number T-8304p).

#### Additional material examined

Two additional populations were collected also in the rhizosphere of cultivated olive at Chiclana de Segura, Jaén province, Spain (38.290469 N latitude, −3.051543 W longitude), and the rhizosphere of wild olive (*Olea europaea* ssp. *sylvestris* Brot.) at Tarifa, Cádiz province, Spain (36.064279 N latitude, −5.673619 W longitude).

#### Etymology

The species epithet refers to a compound adjective formed from *andaluso*‑, referring to Andalusia in southern Spain, and ‑*marocense*, derived from “Maroc”, the French name of Morocco, the places from which the species was collected.

#### Description

*Female*. Body cylindrical, tapering towards the anterior extremity and more abruptly at the posterior end; habitus slightly curved ventrally, forming a C- to J- shaped profile. Cuticle 2.0–2.5 µm thick along the body, becoming more thickened on the tail, where it reaches 11.5–17.0 µm in thickness. Lip region broadly rounded, 1.8–2.6 times wider than high, clearly set off from the body by a conspicuous constriction; amphidial fovea stirrup-shaped, with aperture forming a straight transverse slit, occupying approximately 60–65% of the lip region width and situated just above the corresponding constriction. Odontostyle long and slender, 8.3–9.7 times the lip-region diameter or 1.5–1.9 times the odontophore length, with well-developed flanges, 9.0–11.0 µm wide. Guiding ring double, guiding sheath 8.0–9.0 µm long. Pharynx dorylaimoid, consisting of an anterior slender portion (191–407 µm long) and a posterior expansion forming a terminal pharyngeal bulb, which occupies 27.4–37.4% of the total pharyngeal length. Pharyngeal bulb containing three gland nuclei: the larger dorsal nucleus (4.0–4.5 µm wide) situated at the beginning of the pharyngeal bulb at 12.4–14.0% of the pharyngeal bulb length, and two smaller ventro-sublateral nuclei (2.5–3.0 µm wide) located at approximately the same level, at 54.6–55.8% of the pharyngeal-bulb length. Glandularium 95–130 μm long. Pharyngo-intestinal valve conoid-rounded, 6.5–7.0 µm long. Intestine simple; prerectum 410–580 µm long, rectum 1.0–1.2 anal body diameters in length. Reproductive system didelphic-amphidelphic, with both genital branches equally developed. Ovaries reflexed, 55.0–63.0 µm long. Oviduct comprising a slender proximal section and a *pars dilatata oviductus*, the latter separated from the uterus by a sphincter. Uterus consisting of a wide and elongate *pars dilatata uteri*, a tubular portion, and a large ovejector (24.0–38.0 µm wide). Vagina perpendicular to body axis, 14.5–19.0 μm long; vulva a transverse slit. In the uterine region adjacent to the *pars dilatata uteri*, a rudimentary pseudo-Z-organ is present, composed of a variable number of small granular bodies, generally rounded. In the tubular portion of the uterus, spines, apparently attached to the internal uterine wall, and some crystalloid bodies are present. Sperm not observed in the uterus of any female. Tail conoid with almost straight or slightly ventrally curved profile, whereas the dorsal profile is regularly curved and somewhat digitate. Two caudal pores are visible on each side of the tail (Figs. 10, 11).

*Male.* Not found.

**Fig. 12:**
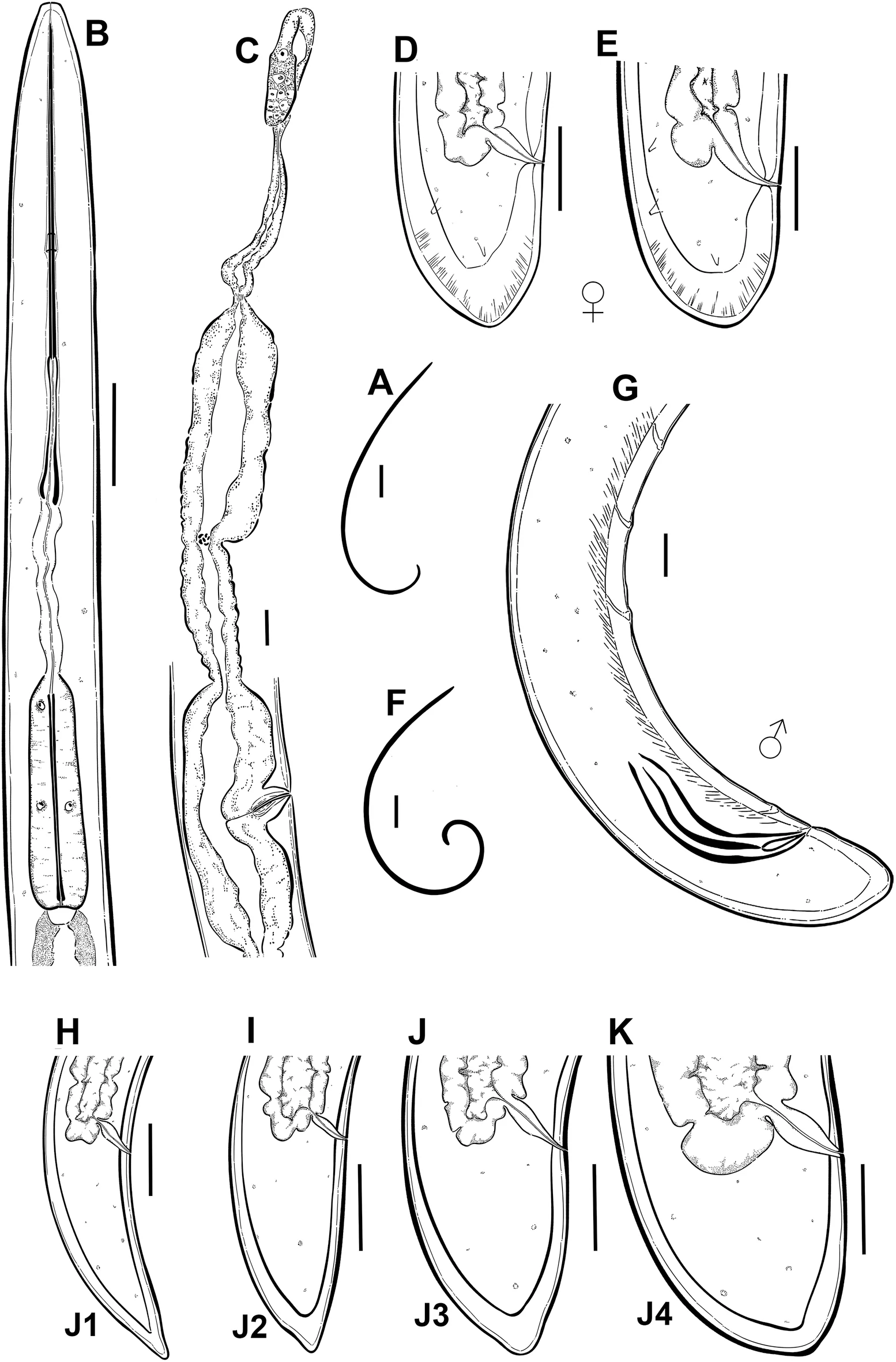
Line drawings of *Xiphinema africana* sp. nov. **A**, entire female; B, female neck region; **C**, detail of anterior genital branch and vulval region; **D**, **E**, female tail region; **F**, entire male; G, male tail region; **H**-**K**, first-, second-, third-, and fourth-stage juvenile tails (**J1**–**J4**), respectively. Scale bars: A, F = 500 μm; B = 500 μm; C-K = 20 μm

*Juveniles.* All four juvenile stages (first-, second-, third- and fourth-stage) were identified using morphological and morphometric characters, including body length and the lengths of the replacement and functional odontostyles [5] (Table 3, Fig. 12). First-stage juveniles (J1) were characterised by the position of replacement odontostyle located just posterior to the functional odontostyle. The tail is elongate-conoid, terminating in an expansion of variable development, separated from the anterior part of the tail by a more or less distinct depression, giving the tail a highly characteristic profile (Figs. 10, 11); the c’ ratio is 3.1–3.3; and the odontostyle measures approximately 48 μm. The tail morphology of second- and third- stage juveniles is elongate-conoid with rounded terminus, whereas in the fourth-stage juvenile it is conoid and subdigitate (Figs. 10, 11).

#### Diagnosis and relationships

*X. andalusomarocense* sp. nov. is a member of the *X.* non-*americanum* group and belongs to morphospecies Group 5 *sensu* Loof & Luc [33]. It appears to be parthenogenetic species characterised by a moderately long body (3.5–4.6 mm), assuming an open C-shape when heat-relaxed; a broadly rounded lip region clearly set off from the body by a conspicuous constriction (12.0–13.5 μm wide); a moderately long odontostyle (105.0–119.0 μm); a vulva situated approximately at mid-body; reproductive system didelphic-amphidelphic, with both branches similarly developed; the Z-differentiation comprises numerous small granular bodies; uterus tripartite containing a low number of small crystalloid bodies and spiniform structures, and exhibiting prominent wrinkles in the uterine wall; female tail conoid with a somewhat digitate terminus; c*´* ratio ranges from 1.5 to 2.0. The species is further distinguished by its specific D2–D3 rRNA, ITS1 rRNA, partial 18S rRNA, and COI sequences. According to the polytomous key of Loof & Luc [33], the new species corresponds to the following specific alphanumeric codes: A4–B2+3-C3–D4–E5–F4–G2–H2–I3–J3–K2–L1.

According to the morphometric data and the polytomous key by Loof & Luc [33], and based on matrix codes for A (type of female genital apparatus), B (uterine differentiation), C (tail shape), D (c´ ratio), E (vulva position), F (body length), and G (total stylet length: odontostyle + odontophore), *X*. *andalusomarocense* sp. nov. groups with *X. abrantinum* Roca & Pereira, 1991 [52], *X*. *diannae* Kruger & Heyns, 1987 [53], *X. hispidum* Roca & Bravo, 1994 [54], *X. lupini* Roca & Pereira, 1993 [55], and *X*. *meridianum* Heyns, 1971 [56]. *X. andalusomarocense* sp. nov. can be clearly differentiated from these species as follows: from *X. abrantinum* by the presence of uterine spines in the new species (absent in *X. abrantinum*), its slightly higher c’ ratio (1.5–2.0 vs 1.2–2.3) [52], and clear differences in D2–D3 rRNA sequences; from *X. diannae* by the presence of uterine Z-differentiation (absent in *X. diannae* which instead has spiniform structures and crystalloid bodies), its longer body and odontostylet (3.5–4.6 mm, 105–119 µm vs 2.7–3.2 mm, 87–94 µm), and its higher c ratio (66.5–98.0 vs 50–62) [53]; from *X. hispidum* by its shorter odontostyle (105–119 µm vs 114–131 µm), the absence of males (present in *X. hispidum*) [54], and clear differences in D2–D3 and ITS1 rRNA sequences; from *X. lupini* by its lower c’ ratio (1.5–2.0 vs 2.1–2.8), slightly lower a ratio (84.9–107.4 vs 116–143), and the absence of males (present in *X. lupini*) [55], and clear differences in D2–D3 and ITS1 rRNA sequences; and from *X. meridianum* by the presence of uterine spines (absent in *X. meridianum*), its slightly longer body (3.5–4.6 mm, 105–119 µm vs 3.1–3.8 mm, 95–102 µm) [56], and clear differences in D2–D3 and ITS1 rRNA sequences.

#### Ribosomal and mitochondrial DNA characterisation

The molecularly closest species to *X. andalusomarocense* sp. nov. were consistent across all three ribosomal regions examined. *X. hispanum*, *X. malaka*, *X. histriae,* and *X. subbaetense* showed the highest similarity values, ranging from 96 to 97% for D2–D3 region (differing by 23–33 nucleotides and one indel), 87% for ITS1 (differing by 135–148 nucleotides and 44–46 indels), and 99% for the partial 18S rRNA gene. Intraspecific variability was detected among the thirteen D2–D3 sequences obtained for *X. andalusomarocense* sp. nov. (PZ458835–PZ458847) and the six ITS1 sequences (PZ458570–PZ458575). Specifically, the D2–D3 region exhibited 28 nucleotide differences and no indels, whereas the ITS1 region 40 nucleotide differences and 11 indels. No intraspecific variation was detected in the partial 18S rRNA sequences of *X. andalusomarocense* sp. nov. (PZ458890–PZ458892). Finally, partial COI sequences of *X. andalusomarocense* sp. nov. (PZ464576–PZ464581) showed 85% similarity to *X. celtiense* (KY816600, differing by 44 nucleotides and no indels. Intraspecific variation among the six COI accessions of *X. andalusomarocense* sp. nov. consisted of 25 nucleotide differences and no indels.

**Fig. 13:**
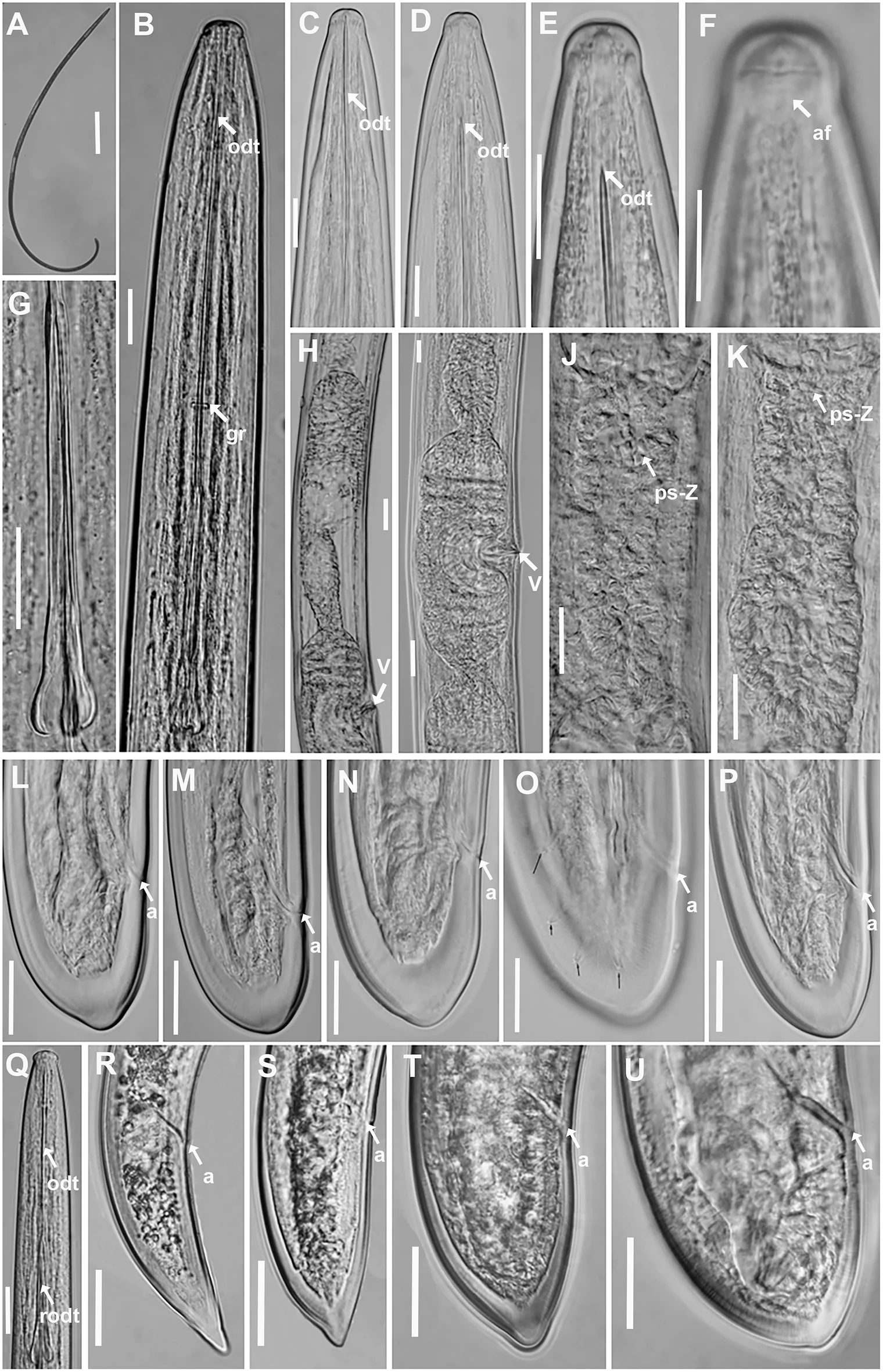
Light micrographs of *Xiphinema africana* sp. nov. **A**, entire female; **B**, female neck region; **C**–**F**, female anterior region; **G**, detail of odontophore; **H**-**I**, vulval region; **J**-**K**, detail of pseudo-Z organ with rounded granular bodies; **L**–**P**, female tail region showing caudal pores (arrowed); **Q**, first-stage juvenile anterior region; **R**–**U**, first-, second-, third-, and fourth-stage juvenile tails (**J1**–**J4**), respectively. Abbreviations: a = anus; gr = guiding ring; odt = odontostyle; ps-Z = pseudo-Z organ; rodt = replacement odontostyle; V = vulva. Scale bars: A = 500 μm; B-*U* = 20 μm

**Fig. 14:**
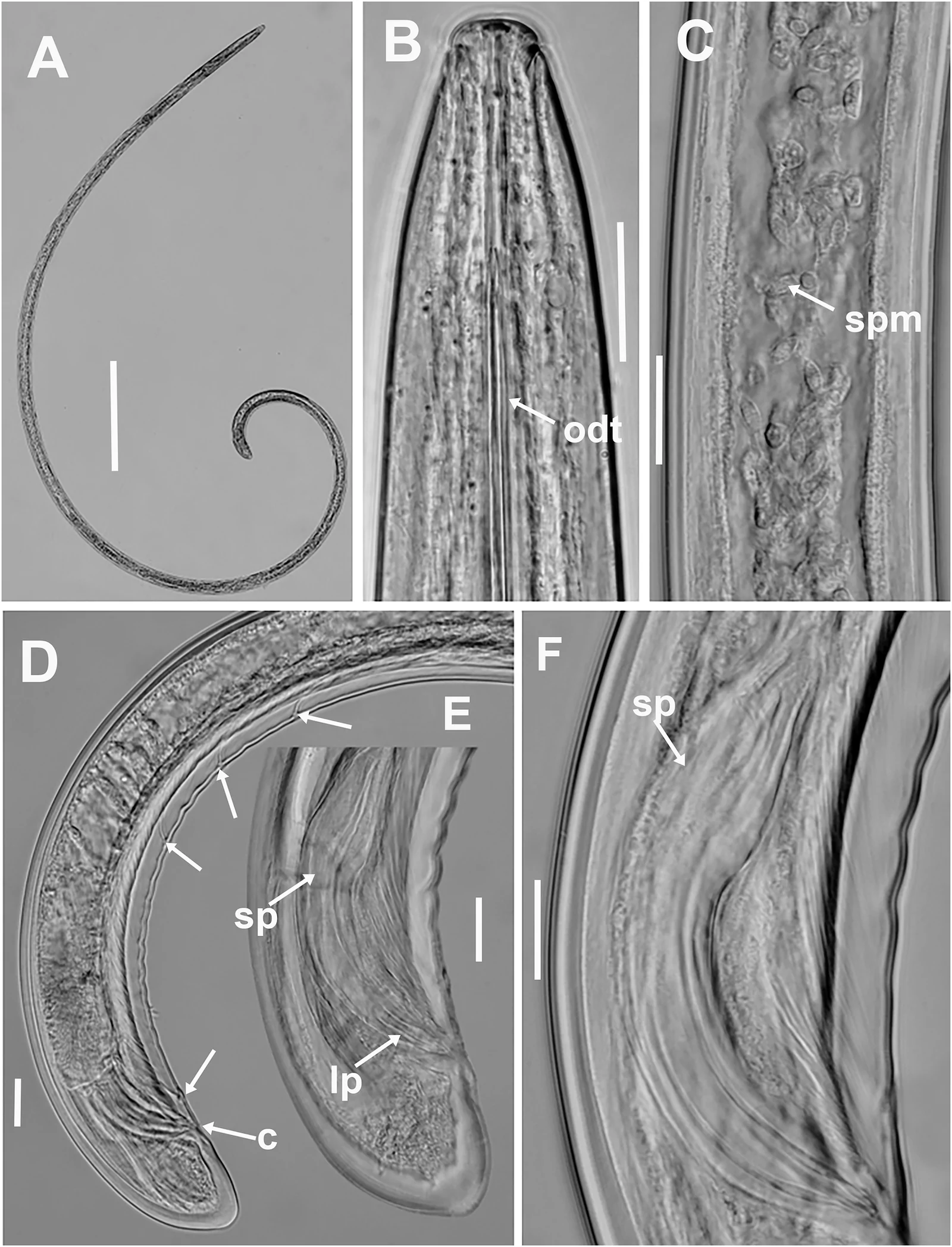
Light micrographs of *Xiphinema africana* sp. nov. **A**, entire male; **B**, male anterior region; **C**, detail of sperm cells; **D**, **E**, male tail region showing midventral supplements (arrowed); **F**, detail of spicules. Abbreviations: c = cloaca; lp = lateral piece; odt = odontostyle; sp = spicules; spm = sperm cells. Scale bars: A = 500 μm; B-F = 20 μm

**Fig. 15:**
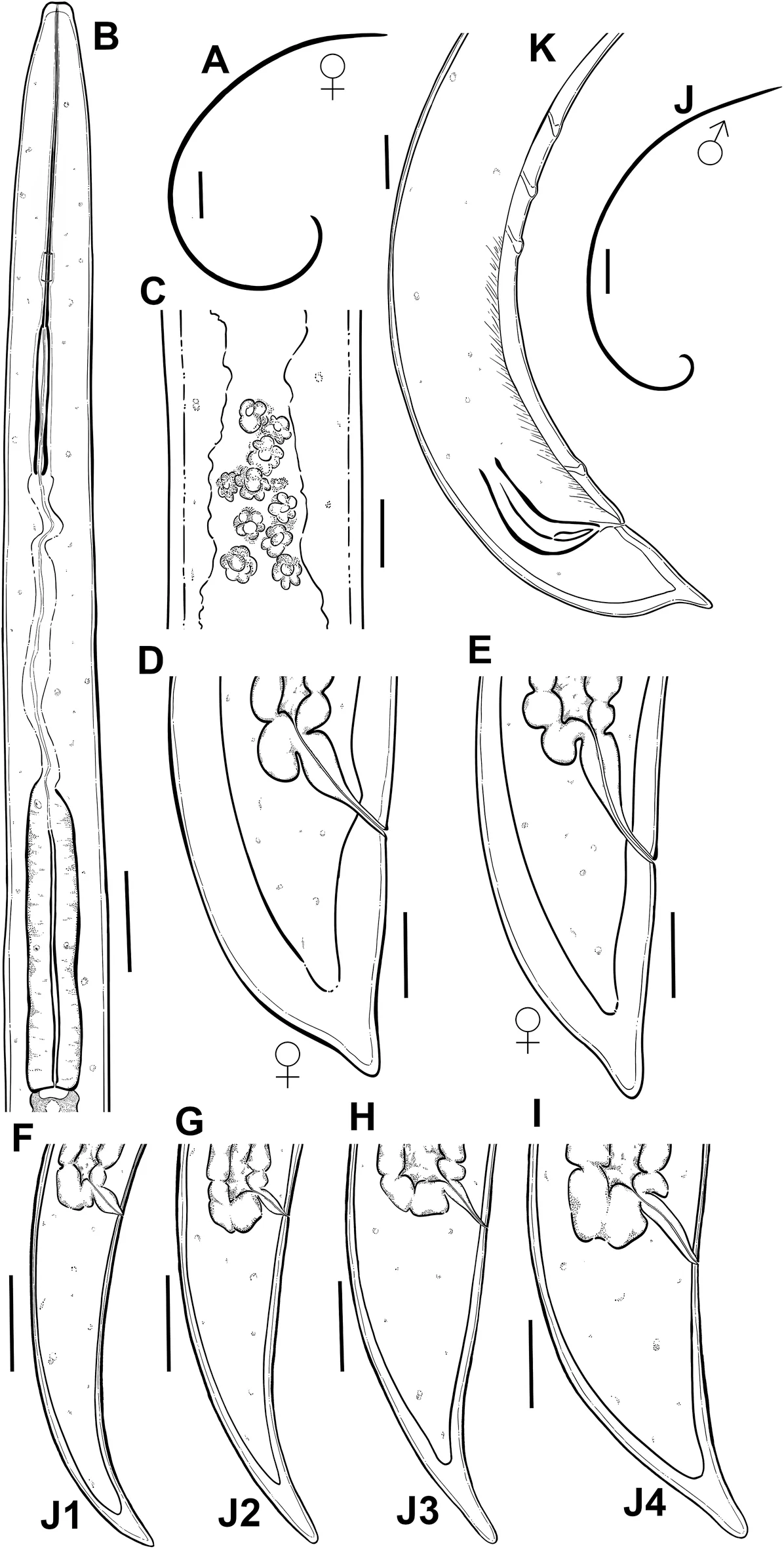
Line drawings of *Xiphinema alentejanum* sp. nov. **A**, entire female; **B**, female neck region; **C**, detail of pseudo-Z organ; **D**, **E**, female tail region; **F**, entire male; **G**, male tail region; **H**–**K**, first-, second-, third-, and fourth-stage juvenile tails (**J1**–**J4**), respectively. Scale bars: A, J = 500 μm; B = 50 μm; C-I, K = 20 μm

***Xiphinema africana*** **sp. nov.** (Figs. 12, 13, 14 and 15; Table 4)

**Table 4:** Morphometrics of *Xiphinema africana* sp. nov. holotype and paratypes from Mediterranean fan palm (*Chamaerops humilis* L.) at Demnate, Azilal, Morocco, and olive (*Olea europaea* subspp. *europaea*) from Al Manzah, Meknes province, Morocco. All measurements are in µm, except for body length in mm and in the form: mean ± s.d. (range)

Character/ratio^1^	Holotype	Paratypes						Al Manzah, Meknes, Morocco
		Females	Males	J1	J2	J3	J4	Females
n		12	4	4	4	5	5	5
L	4732	4800 ± 334	4576 ± 358	1479 ± 81	2233 ± 127	2698 ± 154	3782 ± 217	5174 ± 153
		(4029–5246)	(4246–5083)	(1398–1550)	(2103–2355)	(2513–2896)	(3564–4081)	(4915–5311)
a	67.6	69.0 ± 4.6	87.1 ± 9.4	51.1 ± 3.8	57.3 ± 2.7	64.6 ± 2.1	67.7 ± 5.1	72.2 ± 5.0
		(60.1–76.1)	(78.1–99.7)	(46.9–55.9)	(55.0–61.3)	(62.3–67.3)	(62.2–75.8)	(64.7–76.1)
b	9.2	7.6 ± 0.9	8.3 ± 0.5	5.0 ± 0.3	6.3 ± 0.6	6.5 ± 0.5	6.2 ± 0.6	8.6 ± 0.6
		(6.3–9.2)	(7.7–8.9)	(4.8–5.3)	(5.6–7.0)	(5.8–7.2)	(5.5–6.9)	(8.1–9.5)
c	112.7	118.7 ± 8.7	101.3 ± 1.7	25.3 ± 1.7	45.4 ± 7.0	56.4 ± 2.5	82.4 ± 3.8	124.7 ± 7.3
		(102.0–131.2)	(99.9–103.7)	(23.7–27.2)	(39.3–55.4)	(53.7–59.6)	(76.5–85.9)	(116.0–136.2)
c’	1.0	0.9 ± 0.1	1.0 ± 0.1	2.9 ± 0.2	1.8 ± 0.2	1.5 ± 0.1	1.0 ± 0.1	0.8 ± 0.1
		(0.8–1.0)	(0.9–1.1)	(2.7–3.1)	(1.6–2.0)	(1.4–1.6)	(0.9–1.1)	(0.8–0.9)
d	9.0	9.8 ± 0.8	10.4 ± 0.7	6.1 ± 0.9	6.6 ± 0.3	6.9 ± 0.1	8.7 ± 0.7	9.1 ± 0.9
		(8.5–11.3)	(9.6–11.1)	(5.3–7.4)	(6.3–6.9)	(6.7–7.1)	(7.9–9.7)	(7.7–9.9)
d’	2.7	2.9 ± 0.2	2.8 ± 0.1	2.3 ± 0.2	2.4 ± 0.1	2.3 ± 0.2	2.8 ± 0.2	3.3 ± 0.3
		(2.6–3.3)	(2.8–2.9)	(2.1–2.4)	(2.3–2.5)	(2.1–2.5)	(2.5–2.9)	(3.0–3.6)
V or T	48.8	50.2 ± 0.9	52.3 ± 11.5	-	-	-	-	48.2 ± 0.6
		(48.8–51.3)	(38.8–62.2)					(47.4–49.0)
G_1_	9.2	10.2 ± 1.1	-	-	-	-	-	10.1 ± 0.9
		(8.7–12.2)						(9.2–11.5)
G_2_	9.1	10.0 ± 0.7	-	-	-	-	-	9.6 ± 1.1
		(9.0–10.9)						(8.3–11.0)
Odontostyle	163.0	162.4 ± 4.2	163.5 ± 3.1	75.5 ± 1.3	89.3 ± 4.6	110.2 ± 5.0	144.4 ± 3.5	166.0 ± 4.2
		(155.0–169.0)	(159.0–166.0)	(74.0–77.0)	(86.0–96.0)	(102.0–115.0)	(140.0–149.0)	(162.0–171.0)
Odontophore	88.0	91.6 ± 3.8	90.0 ± 4.2	47.9 ± 2.2	57.5 ± 1.3	71.8 ± 2.2	83.2 ± 5.3	98.4 ± 1.9
		(87.0–97.0)	(87.0–96.0)	(45.0–50.0)	(56.0–59.0)	(69.0–74.0)	(77.0–88.0)	(96.0–101.0)
Total stylet	251.0	254.0 ± 5.9	253.5 ± 6.8	123.4 ± 1.8	146.8 ± 3.9	182.0 ± 6.0	227.6 ± 3.7	264.4 ± 3.8
		(242.0–264.0)	(246.0–262.0)	(122.0–126.0)	(143.0–152.0)	(173.0–189.0)	(223.0–233.0)	(259.0–269.0)
Replacement odontostyle	-	-	-	93.4 ± 2.5	114.5 ± 1.9	137.3 ± 3.4	167.8 ± 5.1	-
				(90.0–96.0)	(113.0–117.0)	(133.0–141.0)	(159.0–172.0)	
Lip region diam.	15.0	14.7 ± 0.7	14.3 ± 0.3	10.1 ± 0.9	11.0 ± 0.8	13.3 ± 0.4	15.1 ± 0.4	16.7 ± 0.8
		(14.0–16.0)	(14.0–14.5)	(9.0–11.0)	(10.0–12.0)	(13.0–14.0)	(14.5–15.5)	(16.0–18.0)
Oral aperture to guiding ring	135.0	143.1 ± 9.5	147.8 ± 6.9	61.5 ± 5.3	72.3 ± 3.2	91.2 ± 2.6	131.1 ± 7.2	153.8 ± 9.4
		(130.0–158.0)	(139.0–156.0)	(56.0–67.0)	(69.0–75.0)	(88.0–95.0)	(122.0–140.0)	(147.0–169.0)
Max. body diam.	70.0	69.6 ± 3.4	52.8 ± 4.3	29.1 ± 2.7	39.0 ± 2.2	41.8 ± 2.4	56.2 ± 6.1	71.8 ± 3.4
		(66.0–76.0)	(48.0–58.0)	(25.0–33.0)	(37.0–42.0)	(38.0–44.0)	(47.0–63.0)	(69.0–76.0)
Tail length	42.0	40.5 ± 1.7	45.1 ± 2.9	58.5 ± 1.3	50.0 ± 7.5	47.9 ± 3.0	45.9 ± 2.0	41.6 ± 2.7
		(38.0–43.5)	(42.0–49.0)	(57.0–60.0)	(42.0–60.0)	(44.0–51.0)	(43.0–48.0)	(38.0–45.0)
J	14.0	15.1 ± 1.0	13.0 ± 2.6	15.8 ± 2.6	12.0 ± 0.8	12.0 ± 1.6	10.8 ± 2.6	15.5 ± 0.6
		(14.0–17.0)	(10.0–16.0)	(13.0–18.0)	(11.0–13.0)	(10.0–14.0)	(8.0–14.0)	(15.0–16.0)
Spicules	-		100.3 ± 6.6	-	-	-	-	-
		-	(96.0–110.0)					
Lateral piece	-	-	22.8 ± 2.2	-	-	-	-	-
			(20.0–25.0)					

^1^Abbreviations as defined: a, body length/maximum body width; b, body length/pharyngeal length; c, body length/tail length; c’, tail length/body width at anus; V (distance from anterior end to vulva/body length) ×100; d = anterior to guiding ring/body diam. at lip region; d’ = body diam. at guiding ring/body diam. at lip region; J = hyaline tail region length

#### ZooBank

**urn:** lsid:zoobank.org:act: 9C648876-7E41-4700-A5B4-4EF8AB2E483A

#### Holotype

Female extracted from a soil sample collected from the rhizosphere of Mediterranean fan palm (*Chamaerops humilis* L.) Demnate, Azilal, Morocco (31.715000 N latitude, −6.993333 W longitude) by A. Bajoub, mounted in pure glycerine and deposited in the Nematode collection of the Laboratorio de Fitonematología, Institute for Sustainable Agriculture, CSIC, Spain (slide number ZN13_2).

#### Paratypes

Females and juvenile stages collected from the rhizosphere of Mediterranean fan palm (*Chamaerops humilis* L.) Demnate, Azilal, Morocco (31.715000 N latitude, −6.993333 W longitude) by A. Bajoub, mounted in pure glycerine and deposited in the Nematode collection of the Laboratorio de Fitonematología, Institute for Sustainable Agriculture, CSIC, Spain (slide number ZN13_6-19); and a female paratype at the USDA Nematode Collection, Beltsville, MD, USA (collection number T-8905p).

#### Additional material examined

An additional population was collected also in the rhizosphere of cultivated olive (*Olea europaea* ssp. *europaea* L.) at Al Manzah, Meknès, Morocco (33.8018341 N latitude, −5.390895 W longitude).

#### Etymology

The species epithet, used as an adjective in the feminine form to agree with the gender of the genus *Xiphinema*, refers to the African continent, from which the species was collected.

#### Description

*Female.* Body cylindrical, tapering very gradually towards the anterior end; habitus slightly ventrally curved, forming a J-shaped profile. Cuticle 3.5–4.5 µm thick along the body, becoming progressively thickened on the tail, where it reaches 14.0–17.0 µm. Lip region broadly rounded, 2.1–3.1 times wider than high, clearly set off from the body by a deep depression; lamphidial fovea stirrup-shaped, with aperture forming a straight transverse slit that occupies approximately 65–72% of the lip region width and situated just above the corresponding depression. Odontostyle long and slender, 10.1–12.1 times the lip-region diameter or 1.7–1.9 times the odontophore length, with well-developed flanges, 14.0–14.5 µm wide. Guiding ring double, guiding sheat 8.0–9.0 µm long. pharyngeal-bulb length comprising an anterior slender portion (381–634 µm long) and a posterior expansion forming a terminal pharyngeal bulb, which occupies 20.9–25.7% of the total pharyngeal length. Pharyngeal bulb contains three gland nuclei: a larger dorsal nucleus (4.0–4.5 µm wide) situated near the anterior margin of the bulb, at 11.3–18.6% of the pharyngeal bulb length, and two smaller ventro-sublateral nuclei (2.5–3.0 µm wide) positioned at approximately the same level, at 44.9–53.8% of the pharyngeal-bulb length. Glandularium 120–140 μm long. Pharyngo-intestinal valve conoid-rounded, 6.5–7.5 µm long. Intestine simple; prerectum 512–657 µm long, rectum 0.6–0.9 anal body diameters in length. Reproductive system didelphic-amphidelphic, with both genital branches equally developed. Ovaries reflexed, 55.0–98.0 µm long. Oviduct consisting of a slender proximal section and a *pars dilatata oviductus*, the latter separated from the uterus by a sphincter. Uterus composed of a wide and elongate *pars dilatata uteri*, a tubular portion (93–129 × 24–29 µm), and a very large ovejector (52.0–67.0 µm wide). Vagina perpendicular to the body axis, 20.0–28.0 μm long; vulva a transverse slit. Adjacent to the *pars dilatata uteri*, a rudimentary pseudo-Z-organ is present, consisting of a 4–6 medium-sized granular bodies, generally rounded. No spines or crystalloid bodies were detected in the tubular portion of the uterus. Sperm observed in the uterus of several females. Tail broadly convex-conoid, with a slight central bulge and a rounded terminus. Four caudal pores present on each side. (Figs. 13, 14).

*Male.* Functional but less abundant than females (ratio = 1:3). Reproductive system diorchic, with testes occupying 39–62% of body length, and containing spindle-shaped sperms. Spicules dorylaimoid, very massive, well sclerotised, 96–110 μm long, ventrally curved, with tubular lateral guiding pieces 20–25 μm long. One pair of adanal supplements situated 17–20 μm from cloacal opening, followed by a series of four (rarely three) ventromedian supplements. Tail similar to that of the female, but more strongly convex dorsally than female, ending in a rounded terminus with a short bulge (Fig. 15).

*Juveniles.* All four juvenile stages (first, second, third and fourth) were identified using morphological and morphometric characters, including body length and the lengths of the replacement and functional odontostyles [5] (Table 4, Fig. 14). J1 is characterised by the position of the replacement odontostyle, located just posterior to the functional odontostyle. The J1 tail is conoid-elongate, with a c’ ratio of approximately 3.0, and an odontostyle around 75 µm long (Figs. 13 and 14). The tail morphology of second- and third-stage juveniles is convex-conoid with a rounded, bulged terminus, whereas in the fourth-stage juvenile it resembles that of the female but with a larger bulge (Figs. 13 and 14).

#### Diagnosis and relationships

*X. africana* sp. nov. is a member of the *X.* non-*americanum* group and belongs to morphospecies Group 5 *sensu* Loof & Luc [33]. It is an amphimictic species characterised by a long body (4.0–5.2 mm), adopting a J-shaped habitus when heat-relaxed; a broadly rounded lip region distinctly set off from the body by a deep depression (14.0–16.0 μm wide); a long odontostyle (155.0–169.0 μm); and vulva located at mid-body. The reproductive system is didelphic-amphidelphic, with both branches equally developed; the Z-differentiation consists of 4–6 medium-sized, generally rounded granular bodies; uterus tripartite and lacks spines or crystalloid bodies; female tail broadly convex-conoid, with a slight central bulge and a rounded terminus; the c*´* ratio ranges from 0.8 to 1.0; and males with long dorylaimoid spicules (96–110 μm). The species is further distinguished by its unique D2–D3 rRNA, ITS1 rRNA, partial 18S rRNA, and COI sequences. According to the polytomous key of Loof & Luc [33], the new species corresponds to the following specific alphanumeric codes (exceptions in parentheses): A4–B2–C5b-D6–E5–F5(4)-G4(3)-H2–I3–J5–K2–L2.

Based on morphometric data and the polytomous key by Loof & Luc [33], and considering matrix codes for A (type of female genital apparatus), B (uterine differentiation), C (tail shape), D (c’ ratio), E (vulva position), F (body length), and G (total stylet length: odontostyle + odontophore), *X*. *africana* sp. nov. groups with *X. cadavalense*, *X. hispanum*, *X. ingens* Luc & Dalmasso, 1963 [57], and *X*. *macroacanthum* Lamberti et al. 1992 [10]. *Xiphinema africana* sp. nov. can be clearly distinguished from these species as follows: from *X. cadavalense* by the absence of uterine spines in the new species (present in *X. cadavalense*), its slightly higher a ratio (60.1–76.1 vs 45.5–68.0), the uterine Z-differentiation (4–6 medium-sized, generally rounded granular bodies in the new species vs ten to fifteen irregularly refractive, sclerotised rosette bodies in *X. cadavalense*) [34], and clear differences in D2–D3 and ITS1 rRNA sequences; from *X. hispanum* by the uterine Z-differentiation (4–6 medium-sized, generally rounded granular bodies in the new species vs numerous small, dense granular bodies in *X. hispanum*), its longer odontostyle (155–169 µm vs 131–142 µm) [10], and clear differences in D2–D3 and ITS1 rRNA sequences; from *X. ingens* by its slightly shorter body (4.0–5.2 mm vs 5.6–5.9 mm), its slightly higher c’ ratio (0.8–1.0 vs 0.6–0.7), its slightly lower c ratio (102.0–131.2 vs 138–168), the presence of males (absent in *X. ingens*) [57], and clear differences in D2–D3 and ITS1 rRNA sequences; and from *X. macroacanthum* by the uterine Z-differentiation (4–6 medium-sized, generally rounded granular bodies in the new species vs eight to ten irregularly refractive, sclerotised rosette bodies in *X. macroacanthum*), its slightly lower c ratio (102.0–131.2 vs 122.5–161.5), its slightly higher c’ ratio (0.8–1.0 vs 0.6–0.7) [10], and clear differences in D2–D3 and ITS1 rRNA sequences.

#### Ribosomal and mitochondrial DNA characterisation

The D2–D3 sequence divergence between *X. africana* sp. nov. (PZ458848–PZ458856) and other congeneric species was substantial, with the highest similarity observed with *X. sphaerocephalum* (KX244917), differing by 44 nucleotides and no indels. Intraspecific variation within the D2–D3 expansion domains of the 28S rRNA gene was low, with only a single variable position detected among the sequences of *X. africana* sp. nov. (PZ458848–PZ458856). High molecular variability was observed in the ITS1 region across *Xiphinema* spp., and only a subset of accessions showed coverage values exceeding 50%. The highest similarity detected for *X. africana* sp. nov. was with *X. meridianum* (KX062700), showing 86% similarity but only 54% coverage. Intraspecific ITS1 variation among the six individuals of *X. africana* sp. nov. (PZ458876–PZ458881) ranged from one to thirteen nucleotide differences. In contrast, high similarity values were obtained for the partial 18S rRNA gene of *X. africana* sp. nov. (PZ458893–PZ458894), which showed 99% similarity to several GenBank accessions, including *X. vuittenezi* (EF614267), *X. malaka* (MT584087), and *X. subbaetense* (MT039137), differing from 12, 15 and 14 nucleotides, respectively. Finally, the six COI sequences of *X. africana* sp. nov. (PZ458893–PZ458894) exhibited considerable divergence from available COI sequences of other *Xiphinema* spp. in GenBank. The closest match was *X. conurum* (KY816603), with 85% similarity and a difference of 42 nucleotides and no indels. Intraspecific variation within the COI region among the six individuals of *X. africana* sp. nov. consisted of 28 nucleotide differences.

**Fig. 16:**
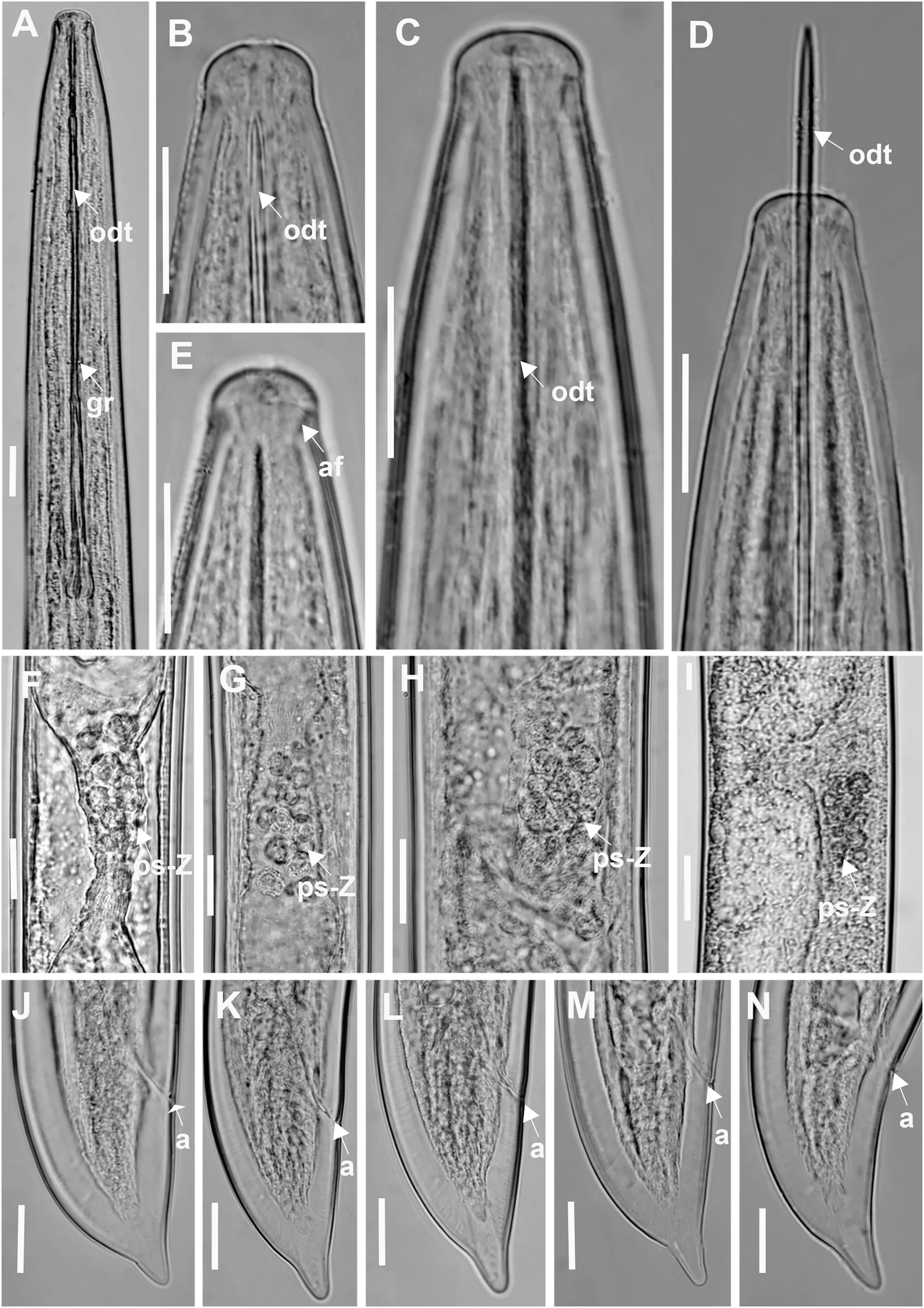
Light micrographs of *Xiphinema alentejanum* sp. nov. **A**, female anterior region; **B**–**E**, female anterior region showing amphidial fovea (arrowed); **F**-**I**, detail of pseudo-Z organ with sclerotised bodies (rossete-like); **J**–**N**, female tail region. Abbreviations: a = anus; af = amphidial fovea; gr = guiding ring; odt = odontostyle; ps-Z = pseudo-Z organ. Scale bars: A-*N* = 20 μm

**Fig. 17:**
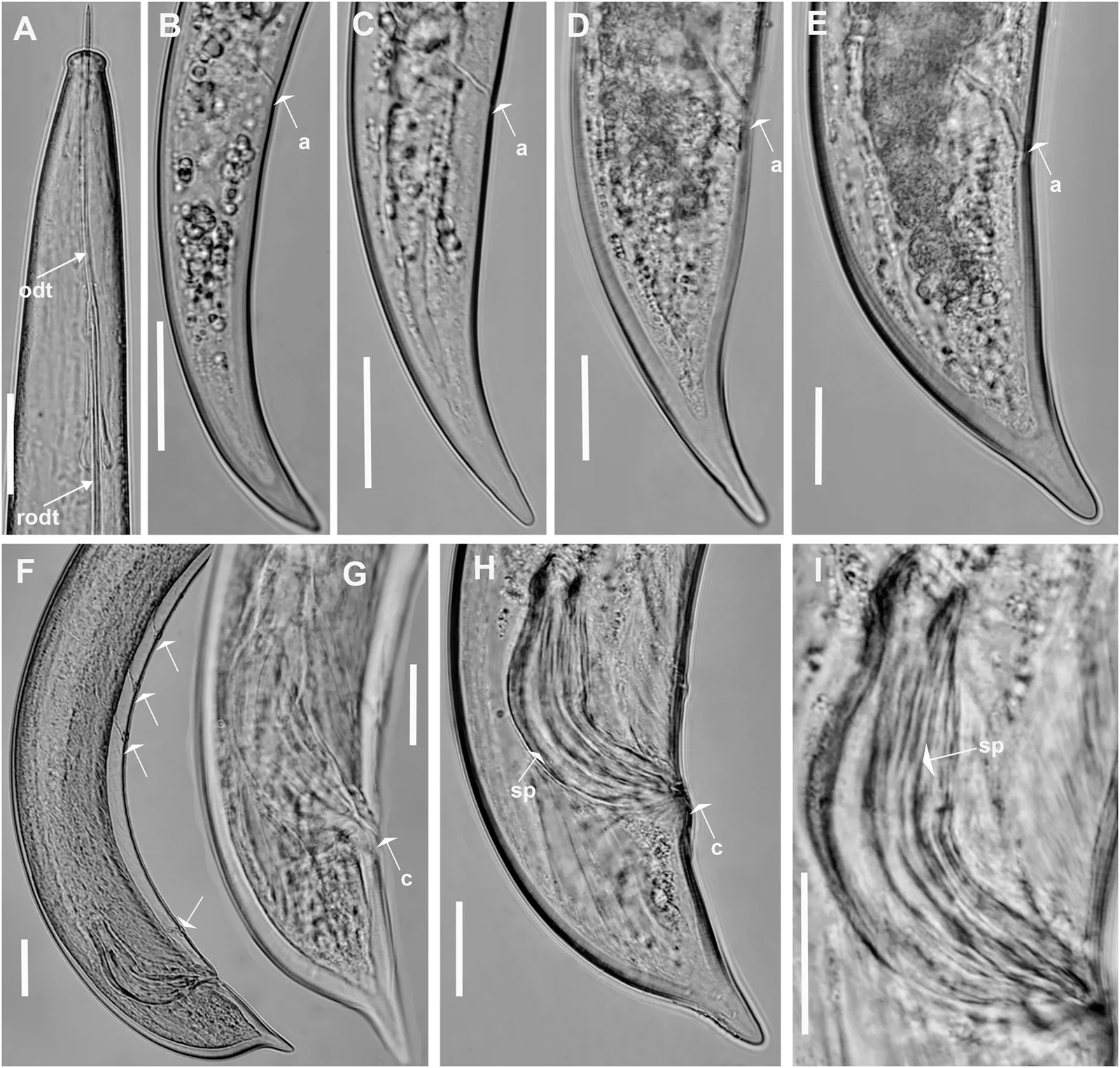
Light micrographs of *Xiphinema alentejanum* sp. nov. **A**, first-stage juvenile anterior region; **B**–**E**, first-, second-, third-, and fourth-stage juvenile tails (**J1**–**J4**), respectively; **F**–**H**, male tail region showing midventral supplements (arrowed); **I**, detail of spicules. Abbreviations: a = anus; c = cloaca; odt = odontostyle; rodt = replacement odontostyle; sp = spicules. Scale bars: A-I = 20 μm

**Fig. 18:**
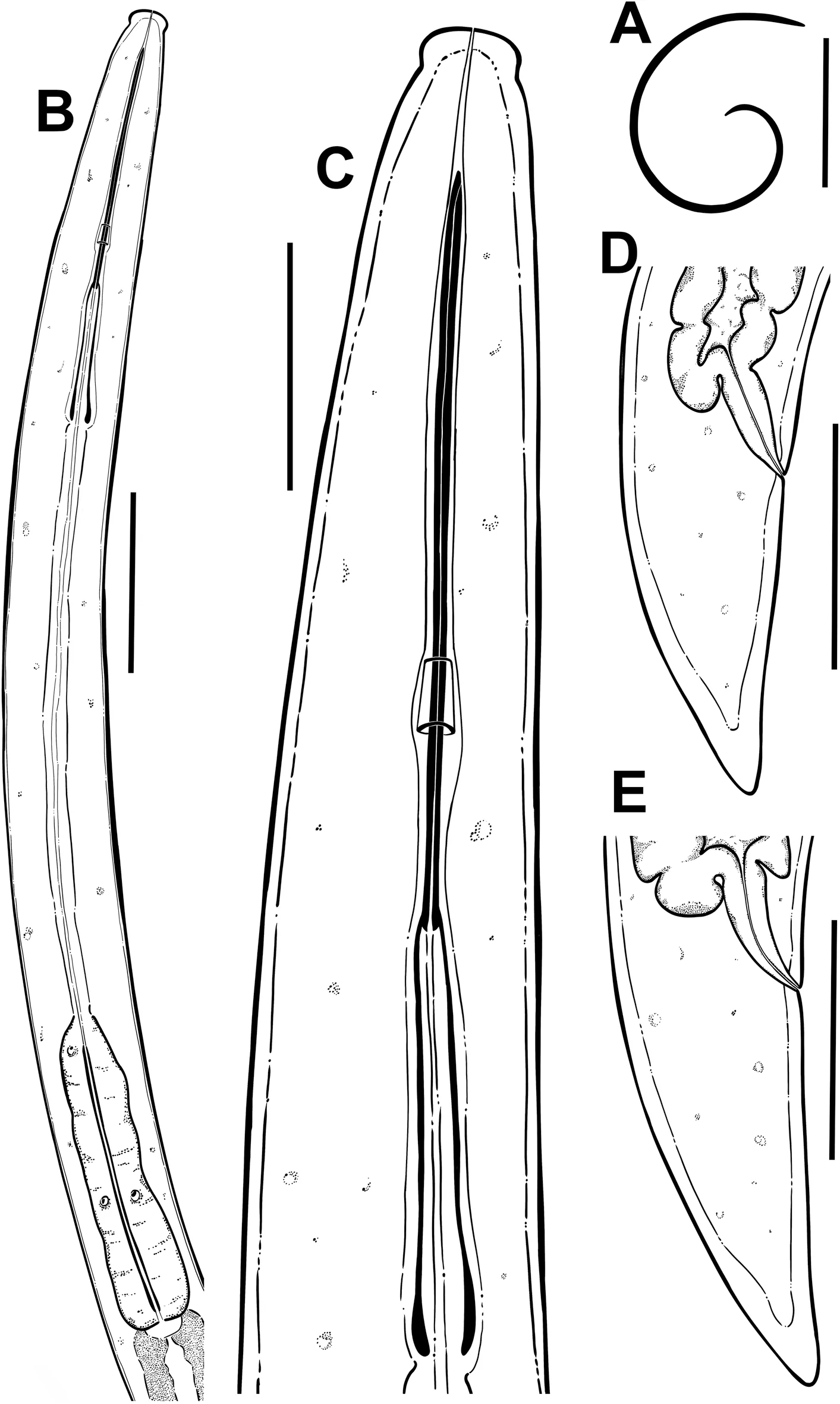
Line drawings of *Xiphinema cryptoduriense* sp. nov. **A**, entire female; **B**, female neck region; **C**, female tail region; **D**, **E**, female tail region. Scale bars: A = 500 μm; B = 50 μm; C-E = 20 μm

***Xiphinema alentejanum*** **sp. nov.** (Figs. 12, 16, 17 and 18; Table 5)

**Table 5:** Morphometrics of *Xiphinema alentejanum* sp. nov. holotype and paratypes from holly oak (*Quercus ilex* L.) at Ferreira do Alentejo, Beja, Portugal. All measurements are in µm and in the form: mean ± s.d. (range)

Character/ratio^1^	Paratypes						
	Holotype	Females	Males	J1	J2	J3	J4
n		21	8	5	4	5	5
L	5989	6120 ± 501	5732 ± 679	1362 ± 96	1821 ± 73	2988 ± 279	4.663 ± 0.267
		(5058–7449)	(4947–6591)	(1271–1501)	(1723–1896)	(2692–3407)	(4.299–4.918)
a	99.8	104.3 ± 8.2	108.8 ± 8.7	50.2 ± 5.0	55.6 ± 0.4	70.7 ± 7.8	87.1 ± 4.3
		(85.7–126.3)	(96.2–119.8)	(42.4–55.6)	(55.0–56.0)	(62.1–83.1)	(83.2–92.3)
b	13.4	11.5 ± 0.8	11.9 ± 0.9	6.5 ± 0.5	5.3 ± 0.8	7.2 ± 0.6	8.9 ± 0.7
		(9.4–13.4)	(10.9–13.5)	(5.8–7.1)	(4.5–6.3)	(6.2–7.6)	(8.0–9.6)
c	93.6	102.3 ± 8.4	96.3 ± 11.5	19.0 ± 1.6	25.9 ± 0.8	39.9 ± 3.6	64.5 ± 7.0
		(88.7–120.1)	(78.5–111.1)	(17.6–21.4)	(25.0–26.7)	(37.3–46.0)	(57.3–75.4)
c’	1.6	1.5 ± 0.1	1.5 ± 0.1	4.1 ± 0.4	3.2 ± 0.2	2.4 ± 0.1	1.8 ± 0.1
		(1.3–1.6)	(1.3–1.6)	(3.6–4.5)	(3.0–3.4)	(2.3–2.7)	(1.6–2.0)
d	8.9	9.1 ± 0.8	8.6 ± 0.6	5.7 ± 0.5	6.9 ± 0.9	8.0 ± 0.9	8.6 ± 0.4
		(6.3–10.3)	(7.6–9.3)	(5.3–6.2)	(6.0–7.7)	(7.2–9.1)	(8.1–9.2)
d’	2.6	2.7 ± 0.1	2.7 ± 0.1	2.1 ± 0.1	2.3 ± 0.1	2.6 ± 0.2	2.5 ± 0.1
		(2.4–2.9)	(2.5–2.8)	(2.1–2.2)	(2.2–2.5)	(2.5–2.9)	(2.3–2.6)
V or T	47.7	47.3 ± 1.0	47.0 ± 3.8	-	-	-	-
		(44.9–49.3)	(39.9–52.0)				
G_1_	9.3	11.3 ± 2.0	-	-	-	-	-
		(8.5–15.0)					
G_2_	9.4	11.4 ± 2.4	-	-	-	-	-
		(8.5–17.2)					
Odontostyle	164.0	162.3 ± 4.5	161.9 ± 5.3	69.4 ± 0.5	90.3 ± 2.6	121.4 ± 3.0	136.4 ± 3.5
		(154.0–170.0)	(152.0–167.0)	(69.0–70.0)	(88.0–93.0)	(118.0–125.0)	(133.0–142.0)
Odontophore	90.0	87.4 ± 3.1	89.1 ± 2.9	41.0 ± 2.3	52.8 ± 1.0	61.0 ± 1.6	78.6 ± 2.1
		(80.0–92.0)	(86.0–94.0)	(39.0–45.0)	(52.0–54.0)	(59.0–63.0)	(76.0–81.0)
Total stylet	254.0	249.7 ± 6.3	251.0 ± 6.6	110.4 ± 2.8	143.0 ± 3.6	182.4 ± 2.7	215.0 ± 4.4
		(240.0–262.0)	(242.0–261.0)	(108.0–115.0)	(140.0–147.0)	(179.0–186.0)	(210.0–222.0)
Replacement odontostyle	-	-	-	87.4 ± 3.2	113.5 ± 2.6	138.4 ± 4.8	158.6 ± 7.9
				(85.0–93.0)	(111.0–117.0)	(132.0–144.0)	(149.0–165.0)
Lip region diam.	15.0	15.0 ± 0.4	15.1 ± 0.4	9.2 ± 0.4	9.8 ± 0.3	11.8 ± 0.4	14.3 ± 0.7
		(14.5–16.0)	(14.5–16.0)	(8.5–9.5)	(9.5–10.0)	(11.0–12.0)	(13.5–15.0)
Oral aperture to guiding ring	133.0	137.0 ± 12.0	129.1 ± 6.0	52.4 ± 2.3	67.0 ± 8.8	94.4 ± 11.7	122.8 ± 1.6
		(101.0–160.0)	(122.0–140.0)	(50.0–56.0)	(59.0–76.0)	(84.0–109.0)	(121.0–125.0)
Max. body diam.	60.0	58.7 ± 2.1	52.6 ± 3.6	27.2 ± 1.6	32.8 ± 1.3	42.4 ± 2.9	53.6 ± 2.9
		(54.0–62.0)	(45.0–56.0)	(26.0–30.0)	(31.0–34.0)	(38.0–45.0)	(51.0–58.0)
Tail length	64.0	60.0 ± 4.0	59.6 ± 2.9	71.8 ± 2.9	70.3 ± 2.1	75.0 ± 4.6	72.6 ± 4.3
		(50.0–66.0)	(57.0–65.0)	(70.0–77.0)	(68.0–72.0)	(68.0–80.0)	(65.0–75.0)
J	20.0	20.7 ± 1.5	21.1 ± 2.4	9.0 ± 1.0	14.3 ± 2.1	18.1 ± 1.4	18.3 ± 2.1
		(19.0–24.0)	(18.5–26.0)	(8.0–10.0)	(12.0–16.0)	(16.5–20.0)	(16.0–20.0)
Spicules	-		70.3 ± 3.6	-	-	-	-
		-	(65.0–74.0)				
Lateral piece	-	-	21.4 ± 1.1	-	-	-	-
			(20.0–23.0)				

^1^Abbreviations as defined: a, body length/maximum body width; b, body length/pharyngeal length; c, body length/tail length; c’, tail length/body width at anus; V (distance from anterior end to vulva/body length) ×100; d = anterior to guiding ring/body diam. at lip region; d’ = body diam. at guiding ring/body diam. at lip region; J = hyaline tail region length

#### ZooBank

**urn:** lsid:zoobank.org:act: 649E6FA3-8FEB-4083-ADE7-DABE2FFE3699

#### Holotype

Female extracted from a soil sample collected from the rhizosphere of holly oak (*Quercus ilex* L.) at Ferreira do Alentejo, Beja, Portugal (38.030429 N latitude, −8.11385 W longitude) by A. Archidona-Yuste and V. Lechuga, mounted in pure glycerine and deposited in the Nematode collection of the Laboratorio de Fitonematología, Institute for Sustainable Agriculture, CSIC, Spain (slide number SO22_2).

#### Paratypes

Females and juvenile stages collected from the rhizosphere of holly oak (*Quercus ilex* L.) at Ferreira do Alentejo, Beja, Portugal (38.030429 N latitude, −8.11385 W longitude) by A. Archidona-Yuste and V. Lechuga, mounted in pure glycerine and deposited in the Nematode collection of the Laboratorio de Fitonematología, Institute for Sustainable Agriculture, CSIC, Spain (slide number SO22_3-18); and female and male paratypes at the USDA Nematode Collection, Beltsville, MD, USA (collection number T-8306p).

#### Etymology

The species epithet, is a Latinised adjective meaning from Alentejo, the region of Portugal where the species was discovered.

#### Description

*Female.* Body cylindrical, tapering gradually towards the anterior end; habitus ventrally curved, forming a C-shaped profile. Cuticle apparently smooth 3.0–4.0 µm thick along the body, becoming progressively thickened on the tail, where it reaches 19.0–24.0 µm. Lip region broadly rounded, 3.0–3.3 times wider than high, separated from the rest of the body by a weak depression; amphidial fovea stirrup-shaped, with aperture forming a straight transverse slit that occupies approximately 66–73% of the lip region width and situated just above the corresponding depression. Odontostyle very long and slender, 9.9–11.1 times the lip-region diameter or 1.8–2.1 times the odontophore length, with well developed flanges, 13.0–14.0 µm wide. Guiding ring double, guiding sheath 8.5–9.5 µm long. Pharynx dorylaimoid, comprising of an anterior slender portion (415–656 µm long) and a posterior expansion forming a terminal pharyngeal bulb, which occupies 27.2–39.8% of the total pharyngeal length. Pharyngeal bulb contains three gland nuclei: a larger dorsal nucleus (4.0–4.5 µm wide) situated near the anterior margin of the bulb, at 9.4–12.6% of the pharyngeal-bulb length, and two smaller ventro-sublateral nuclei (2.5–3.0 µm wide) positioned at approximately the same level, at 49.8–52.9% of the pharyngeal-bulb length. Glandularium 121–142 μm long. Pharyngo-intestinal valve conoid-rounded, 6.0–7.0 µm long. Intestine simple; rectum 0.7–0.9 anal body diameters in length. Reproductive system didelphic-amphidelphic, with both genital branches equally developed. Ovaries reflexed, 52.0–78.0 µm long. Oviduct consisting of a slender proximal section and a *pars dilatata oviductus*, the latter separated from the uterus by a sphincter. Uterus composed of a wide and elongate *pars dilatata uteri*, a tubular portion, and a large ovejector (28.0–48.0 µm wide). Vagina perpendicular to the body axis, 17.0–24.5 μm long; vulva a transverse slit. Adjacent to the *pars dilatata uteri*, a pseudo-Z-organ is present, consisting of irregular shape sclerotised bodies, variable in number (nine to 12) and size, and each body consisting of a central, very large, spherical and transparent portion surrounded by irregularly shaped refractive pieces (rosette-like). No spines or crystalloid bodies were detected in the tubular portion of the uterus. Sperm observed in the uterus of several females. Tail short, conoid, slightly rounded dorsally and almost straight ventrally, with distinctly digitate or subdigitate terminus (9.5–13 μm long) ventrally orientated in the caudal axis (Figs. 16, 17). Four or five caudal pores visible on each side of the tail.

*Male.* Functional but less abundant than females (ratio = 1: 2.5). General appearance similar to the female, although the posterior body region is more strongly curved. Reproductive system diorchic, with testes occupying 40–43% of body length and containing spindle-shaped sperm. Spicules arcuate and robust, 1.0–1.3 times the tail length, lateral guiding piece more or less straight or with curved proximal end. One pair of adanal supplements located at 27.6 μm (24.0–31.0 μm) from the cloacal aperture, followed by a series of three (occasionally four) mid-ventral supplements. Tail similar to that of the female, but more strongly convex dorsally (Fig. 17).

*Juveniles:* All four juvenile stages (first to fourth) were identified using morphological and morphometric characters, including body length and the lengths of the functional and replacement odontostyles [5] (Table 5, Figs. 16, 17). J1 is distinguished by the position of the replacement odontostyle, situated immediately posterior to the functional odontostyle. The J1 tail is conoid-elongate, with a c’ ratio of approximately 4.0 (3.6–4.5), and an odontostyle measuring *c*. 70 µm in length (Figs. 16, 17). The tails of second-, third-, and fourth-stage juveniles are likewise conoid-elongate, with a c’ ratios ranging from 3.2, 2.4, and 1.8, respectively (Table 5, Figs. 16, 17).

#### Diagnosis and relationships

*X. alentejanum* sp. nov. is an amphimictic species characterised by a long body (5058–7449 µm); a broadly rounded lip region set off from the body contour by a weak depression; a very long odontostyle and odontophore (154–170 and 80–92 µm, respectively); vulva located at 45–49% of body length; and a well-developed pseudo-Z-organ, composed of nine to twelve rosette-like, irregular shape sclerotised bodies. The female tail is short and conoid, slightly rounded dorsally and almost straight ventrally, with distinctly digitate or subdigitate terminus (9.5–13 μm long) directed ventrally along the caudal axis; the c ratio (body length/tail length) ranges from 88.7–120.1; spicules medium-size (65–74 µm long). The new species belongs to the *X.* non-*americanum* group and is assigned to morphospecies Group 5 *sensu* Loof & Luc [33]. It is further distinguished by its unique sequences of the D2–D3 rRNA, ITS1 rRNA, partial 18S rRNA, and COI genes. According to the polytomous key of Loof & Luc [33], the new species corresponds to the following alphanumeric codes (exceptions in parentheses): A4–B2–C4–D5(4)-E5–F5–G4(3)-H2–I3–J4–K2–L2.

Based on morphometric data and the polytomous key by Loof & Luc [33], and considering matrix codes for A (type of female genital apparatus), B (uterine differentiation), C (tail shape), D (c´ ratio), E (vulva position), F (body length), and G (total stylet length: odontostyle + odontophore), *X*. *alentejanum* sp. nov. groups with *X. baetica* Gutiérrez-Gutiérrez et al. 2013 [6], *X. andalusiense* Archidona-Yuste et al. 2016 [30], *X. gersoni* Roca & Bravo, 1993 [58], and *X*. *macrodora* Archidona-Yuste et al. 2016 [30]. *X. alentejanum* sp. nov. can be distinguished from these species as follows. It is morphologically closet to *X. baetica*, from which it differs by its slightly longer female tail (50–66 μm vs 45–59 μm), slightly longer spicules (65–74 μm vs 61–71 μm), and by J1 morphology and morphometrics, including body length, c’ ratio, odontostyle length and tail length (1270–1500 μm, 3.6–4.5, 69–70 μm, 70–77 μm vs 994–1050 μm, 3.4–3.9, 57–63 μm, 46–48.5 μm), and differences in ITS1 rRNA and COI sequences [6]; from *X. andalusiense* differs by its higher a and c’ ratios (85.7–126.3, 1.3–1.6 vs 68.7–89.3, 1.0–1.3), its longer odontostyle (154–170 μm vs 137–151 μm) [7], and clear differences in D2–D3 and ITS1 rRNA sequences; from *X. gersoni* differs by its slightly lower a, c and c’ ratios (85.7–126.3, 88.7–120.1, 1.3–1.6 vs 101.5–136.5, 89.7–12.7, 1.4–2.0) [58], and clear differences in D2–D3 and ITS1 rRNA sequences; and finally, from *X. macrodora* differs by its shorter body, odontostyle and odontophore (5058–7449 μm, 154–170 μm, 80–92 μm vs 7159–8682 μm, 190–206 μm, 105–120 μm), its higher a and c’ ratios (85.7–126.3, 1.3–1.6 vs 68–86, 0.7–0.9), its lower c ratio (88.7–120.1 vs 124.8–155.5), and clear differences in D2–D3 and ITS1 rRNA sequences [30].

#### Ribosomal and mitochondrial DNA characterisation

The D2–D3 region of *X. alentejanum* sp. nov. (PZ458857–PZ458861) showed 98% similarity to *X. baetica* (KC567167, KX244899), and 97% similarity to *X. andalusiense* (KX244885- KX244887), *X. macrodora* (KU171040, KU171042), and *X. cadavalense* (KX244900). Sequence divergence among these species ranged from 11 to 32 nucleotides and from 2 to 11 indels. The ITS1 region showed a similar pattern: the closest species to *X. alentejanum* sp. nov. (PZ458882–PZ458885) were *X. baetica* (KC567156, 94% similarity, differing by 60 nucleotides and 22 indels), *X. cadavalense* (88% similarity, 63 nucleotides and 24 indels), and *X. andalusiense* (88% similarity, 106 nucleotides and 29 indels). The partial 18S rRNA gene of *X. alentejanum* sp. nov. (PZ458895–PZ458897) exhibited high similarity values (99%) to several *Xiphinema* spp., including *X. baetica* (KC567148–KC567149), *X. andalusiense* (KX244941), *X. cadavalense* (KX244932) and *X. macrodora* (KU171050). Finally, the partial COI sequences of *X. alentejanum* sp. nov. (PZ464588–PZ464592) showed 86% similarity to *X. baetica* (KY816596, KY816597; differing by 47 nucleotides and no indels) and 83% similarity to *X. andalusiense* (KY816594, KY816595, differing by 58 nucleotides and no indels). No intraspecific variation was detected in any of the ribosomal regions analysed. In contrast, COI sequences exhibited intraspecific variation ranging from 0 to 9 nucleotides were detected, with no indels.

### Morphology, morphometrics and molecular characterisation of known *Xiphinema* non-*americanum*-group species

Nine previously described species of the *X.* non*americanum*-group species were identified in this study using at least two molecular markers (D2–D3, ITS1 rRNA, or COI) (Table 1): *X. adenohystherum* Lamberti et al. 1992 [10], *X. ingens*, *X. israeliae* Luc et al.1982 [59], *X. italiae* Meyl, 1953 [60], *X. nuragicum* Lamberti et al. 1992 [10], *X. sphaerocephalum* Lamberti et al.1992 [10], *X. turcicum* Luc & Dalmasso, 1963 [57], *X. zagrosense* Ghaemi et al. 2012 [61]; and one unidentified species (*Xiphinema* sp. non-*americanum* SO20). For species already known from the same regions (e.g. the Spanish populations of *X. adenohystherum*, *X. italiae*, *X. nuragicum* or *X. turcicum*, and the Cretan population of *X. israeliae*), only diagnostic traits were confirmed, as these taxa have been thoroughly characterised in previous studies [6, 7, 10, 62]. Several species are newly recorded, or rarely reported, in specific countries. *X. adenohystherum* and *X. nuragicum* are reported for the first time in Portugal (Table S1, Figures S1, S2); *X. ingens* and *X. zagrosense* are newly recorded from Lesbos Island, Greece (Table S1, Fig. S3); and *X. italiae*, although previously reported from Morocco [63], is documented here for the first time with molecular characterisation (Table S1, Fig. S4). For these species, morphometric data and supplementary remarks are provided, including brief comments and a morphometric and molecular comparison with previous records and paratypes.

*Xiphinema* sp. SO20 was recovered from cultivated olive and holly oak at Bragança. It is characterised by a medium to large body length (4.0–4.5 mm), a moderate odontostyle (128–134 μm), a pseudo-Z organ composed of 4–6 sclerotised rosette-like bodies, crystalloid bodies in the uterus, a convex-conoid female tail with a central peg; spicules ventraly curved 62–64 μm long and lateral piece 15–17 μm long. D2–D3 sequences showed moderate similarity (97.6%) to Portuguese populations identified as *X. abrantinum* (AY601625) and *Xiphinema* sp. SAS-2014 (KJ634067) [64, 65], differing by 17 bp and two indels, and 96.1% similar to several *X. diversicaudatum* sequences. ITS rRNA and COI sequences were markedly different (85.2–87.5% similarity or lower; 123 bp and 37 indels, 53 bp and 0 indels, respectively) from those of other *Xiphinema* species, including *X. diversicaudatum* (HG969304), *X. bakeri* (MK073001), *X. japonicum* (KY131244), *X. cadavalense* (KX244932) and *X. macrodora* (KY816632). ITS or COI sequences are not available for *X. abrantinum* or *Xiphinema* sp. SAS-2014. The combined molecular evidence therefore strongly suggests that *Xiphinema* sp. SO20 may represent a new species, but it is not formally described here due to the limited material available (Table S1).

Two Portuguese populations of *X. adenohystherum* from holly oak and yellow broom at Ferreira do Alentejo (Table S1, Fig. S1) match the original description and Spanish records, with only minor variation in the c ratio and female tail length [6, 10]. D2–D3 and ITS rRNA sequences showed high similarity to Spanish populations (99.5–100% and 98–100%, respectively) [6, 66], differing by 0–4 bp, 12–23 bp and 0, 2–14 indels, respectively. COI sequences showed 91.5–99.2% similarity with Spanish sequences, differing by 3–31 bp and one indel [67].

Similarly, the two Portuguese populations of *X. nuragicum* from cultivated olive and holly oak at Bragança (Table S1, Fig. S2) correspond closely with the original description and Spanish material [6, 10]. D2–D3, ITS rRNA, and COI sequences showed very high similarity to Spanish populations (99.9–100%, 99.8–100% and 99.7–100%, respectively), differing by 0–1 bp, 0–3 bp and 0–3 bp, and by 0–1, 0–2, 0–2 indels, respectively [6, 66, 67].

Two Moroccan populations of *X. italiae* from wild olive and Indian fig opuntia at Meknès (Table S1, Fig. S4) agree with the original description and with Spanish and Montenegrin records [60, 68, 69]. D2–D3 and ITS rRNA sequences showed high similarity to Slovak, Spanish, and Cretan populations (98.6–99.7%, 97.2–98.0%, respectively), differing by 2–10 bp and 1–4 bp, and by 21–28 and 19–26 indels, respectively [7, 62, 66, 68, 70].

Only juvenile stages (J2–J3) were recovered of *X. ingens* from Lesbos, Greece. Their morphometrics match published data [71]: J2: L = 1866 μm, odontostyle = 89 μm, replacement odontostyle = 112 μm; J3: L = 2643 μm, odontostyle = 114 μm, replacement odontostyle = 135 μm. D2–D3 sequences showed high similarity (97.8–99.7%) to Russian and Iranian populations, differing by only 2–16 bp and with no indels [72].

The Lesbos population of *X. zagrosense* from Turkish pine (*Pinus brutia* Ten.) at Plomari (Table S1, Fig. S3) corresponds well with the original Iranian description [61]. However, large (6–7 μm long) and abundant crystalloid bodies were observed in the uterus (Fig. S3), features misinterpreted as long spines in the original description. D2–D3 sequences showed high similarity (99.1–99.5%) to Iranian and Syrian populations [61, 73], differing by 5–6 bp and no indels. ITS rRNA sequences are reported here for the first time and differ markedly from all other *Xiphinema* ITS sequences in NCBI, showing 87.6–90.5% similarity to *X. cretense* Tzortzakakis et al. 2014 [62], *X. israeliae*, and *X. herakliense* Tzortzakakis et al. 2015 [74], with differences of 99–134 bp and 30–43 indels [62, 74].

### Morphology, morphometrics and molecular characterisation of *Xiphinema americanum* group species

Within this group, 23 populations were studied belonging to one new species and five previously described *Xiphinema* species were identified as for the *X.* non-*americanum*-group (Table 1): *X. cryptoduriense* sp. nov., *X. incertum* Lamberti et al. 1983 [27], *X. luci* Lamberti & Bleve-Zacheo, 1979 [75], *X. pachtaicum*, *X. plesiopachtaicum* Archidona-Yuste et al. 2016b [8], and one unidentified species (*X. americanum* group SO20). For the already known and described species, no additional morphological analyses, other than confirmation of diagnostic traits, were undertaken as was the case for the *X.* non-*americanum*-group species (i.e. the Spanish populations of *X. incertum*, *X. pachtaicum*, or *X. plesiopachtaicum*), all of which have already been well characterised, both morphologically and molecularly [6, 8].

**Fig. 19:**
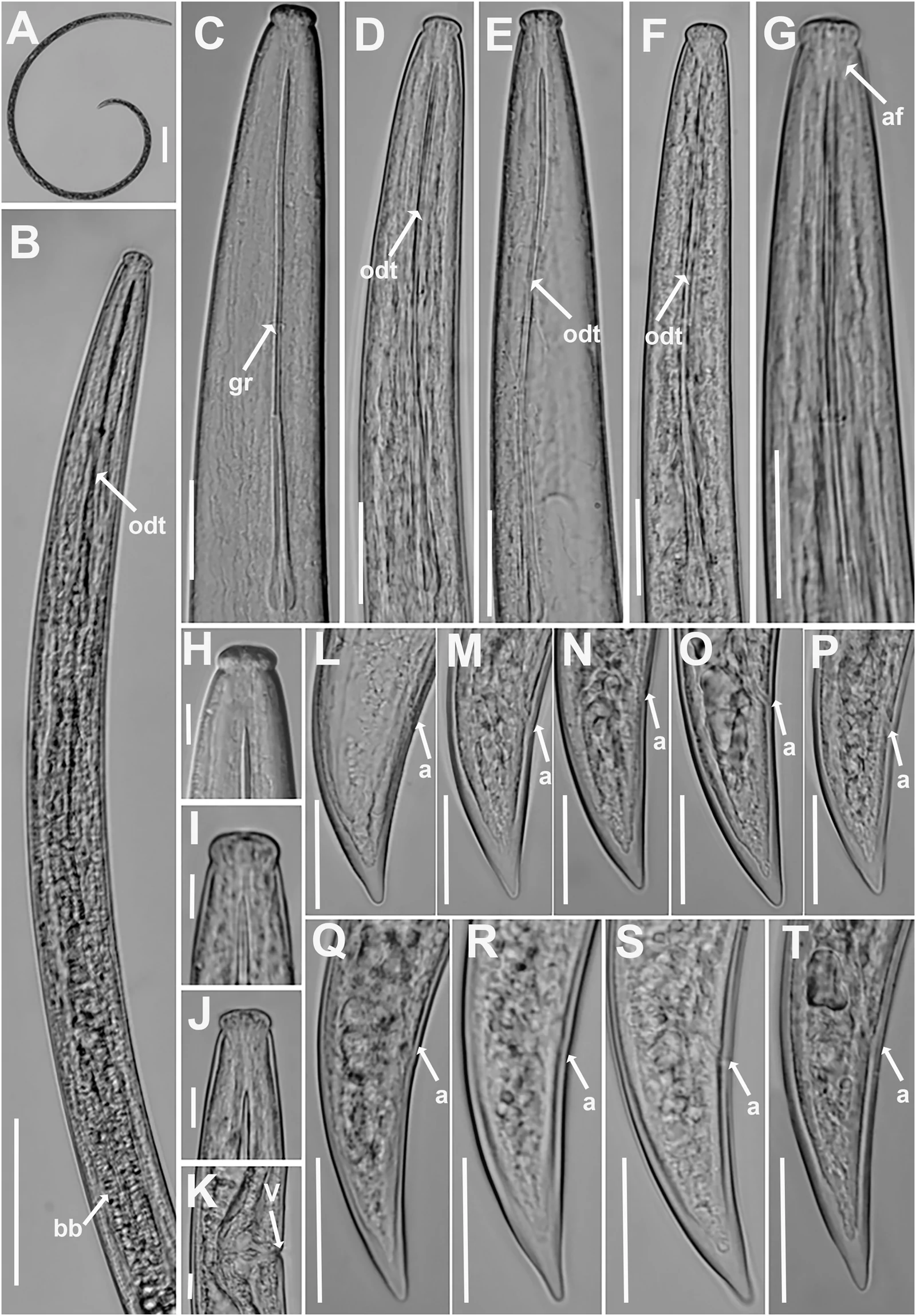
Light micrographs of *Xiphinema cryptoduriense* sp. nov. **A**, entire female; **B**, female neck region; **C**–**G**, female anterior region; **H**–**J**, female lip region; **K**, vulval region; **L**–**T**, female tail region. Abbreviations: a = anus; af = amphidial fovea; bb = basal bulb; gr = guiding ring; V = vulva. Scale bars: A = 100 μm; B = 50 μm; C-G, L-T = 20 μm; H-K = 5 μm

***Xiphinema cryptoduriense*** **sp. nov.** (Figs. 18, 19; Table 6)

**Table 6:** Morphometrics of *Xiphinema cryptoduriense* sp. nov. holotype and paratypes from cultivated olive (*Olea europaea* subspp. *europaea*) at Mirandela, Braganza, Portugal, and morphometrics of *Xiphinema duriense* from other populations reported accross the Mediterranean Basin. All measurements are in µm and in the form: mean ± s.D. (range), except where original literature sources provide data in alternative formats

	*X. cryptoduriense* **sp. nov.**		Xiphinema duriense						
Host-plant	Olive		Grapevine	Weeds	Weeds	Grapevine	Grapevine	Grapevine	Olive
Locality, province, country	Mirandela, Braganza, Portugal		Quinta do Cabo, Oporto, Portugal	Vila Nova de Foz Coa, Guerda, Portugal	Montemor, Coimbra, Portugal	Torre de Moncorvo, Braganza, Portugal	Cidadehle, Guarda, Portugal	Almonte, Huelva, Spain	Gibraleon, Huelva, Spain
Reference	SO18	SO18	Lamberti et al. 1993 [50]	Lamberti et al. 1994 [51]	Lamberti et al. 1994 [51]	Lamberti et al. 1994 [51]	Lamberti et al. 1994 [51]	Gutierrez-Gutierrez et al. 2012 [28]	Archidona-Yuste et al. 2016b [8]
Character/ratio^1^	Holotype	Paratype Females	Paratype Females	Females	Females	Females	Females	Females	Females
n		21	10	10	10	5	5	10	3
L	1662	1659 ± 121	1800 ± 100	1600	1800	1900	1800	1436 ± 122	1309 ± 82
		(1399–1840)	(1600–2000)	(1500–1800)	(1600–1900)	(1800–2000)	(1700–1850)	(1288–1611)	(1239–1400)
a	63.9	66.2 ± 4.6	74 ± 3.9	73.5	72	80	79	61.8 ± 4.4	51.4 ± 3.2
		(56.2–73.6)	(68–81)	(70–81)	(68–75)	(76–85)	(75–81)	(53.7–67.6)	(48.6–54.9)
b	6.2	6.1 ± 0.4	6.0 ± 0.4	5.9	7.0	7.2	6.8	7.2 ± 0.6	5.5 ± 0.3
		(5.5–6.6)	(5.4–6.7)	(5.2–6.5)	(6.1–8.7)	(6.7–7.5)	(6.4–7.1)	(6.5–9.5)	(5.2–5.7)
c	48.2	49.3 ± 3.8	58 ± 5.0	52.5	65	57	53	47.2 ± 4.8	45.4 ± 5.8
		(42.5–57.5)	(53–67)	(47–59)	(58–70)	(53–61)	(51–55)	(39.9–56.5)	(39.3–50.0)
c’	2.3	2.2 ± 0.2	2.1 ± 0.1	2.35	1.8	2.4	2.4	2.2 ± 0.2	1.9 ± 0.1
		(1.8–2.4)	(1.9–2.3)	(2.2–2.5)	(1.6–2.1)	(2.2–2.5)	(2.3–2.6)	(2.0–2.6)	(1.8–1.9)
V	61.1	62.0 ± 0.5	60 ± 1.9	61	64	62	63	65.1 ± 1.2	65.5 ± 1.3
		(61.1–63.1)	(56–63)	(59–65)	(63–67)	(60–63)	(61–65)	(63–67)	(64.5–67.0)
Odontostyle	67.0	68.4 ± 2.0	70 ± 1.4	68	67.5	66	66	71.1 ± 2.0	68.3 ± 1.6
		(66.0–72.0)	(68–72)	(62–71)	(59–72)	(65–68)	(64–68)	(70.0–74.0)	(66.5–69.5)
Odontophore	40.0	39.2 ± 1.1	37 ± 2.2	37	38.5	39	37	36.6 ± 0.9	36.8 ± 2.8
		(38.0–42.0)	(33–41)	(35–38)	(37–40)	(38–41)	(36–38)	(35.0–37.5)	(34.5–40.0)
Lip region diam.	9.0	8.3 ± 0.4	8.1 ± 0.2	8	8.5	8	8	8.0 ± 0.3	8.0 ± 0.3
		(8.0–9.0)	(7.6–8.2)	(8–8)	(8.0–9.0)	(8–8)	(8–8)	(7.5–8.5)	(7.5–8.0)
Oral aperture to guiding ring	58.0	55.6 ± 3.7	61 ± 2.7	57	57.5	53	53	56.3 ± 3.4	57.7 ± 0.6
		(49.0–64.0)	(56–64)	(53–62)	(53–61)	(50–56)	(50–54)	(53.5–60.0)	(57–58)
Tail length	34.5	33.7 ± 1.8	31 ± 2.4	31	28	33	34	30.6 ± 3.1	29 ± 2.2
		(31.0–37.0)	(28–35)	(29.0–33.5)	(24–31)	(29–34)	(33.5–34.0)	(26.5–36.5)	(27.5–31.5)
J	8.0	7.9 ± 0.6	7.3 ± 0.8	7.5	7.5	9	8.5	7.3 ± 1.3	7.0 ± 1.0
		(7.0–9.0)	(5.9–8.2)	(6.5–8.0)	(6.5–9.0)	(8–10)	(8–9)	(6.5–8.5)	(6.0–8.0)

^1^Abbreviations as defined: a, body length/maximum body width; b, body length/pharyngeal length; c, body length/tail length; c’, tail length/body width at anus; V (distance from anterior end to vulva/body length) ×100; d = anterior to guiding ring/body diam. at lip region; d’ = body diam. at guiding ring/body diam. at lip region; J = hyaline tail region length

#### ZooBank

**urn:** lsid:zoobank.org:act: 85D1E370-3907-45F1-A046-6CE4ADFE8BA1

#### Holotype

Female extracted from a soil sample collected from the rhizosphere of cultivated olive (*Olea europaea* ssp. *europaea* L.) at Mirandela, Bragança, Portugal (41.53099 N latitude, −7.047058 W longitude) by J.E. Palomares-Rius and P. Castillo, mounted in pure glycerine and deposited in the Nematode collection of the Laboratorio de Fitonematología, Institute for Sustainable Agriculture, CSIC, Spain (slide number SO18_1).

#### Paratypes

Females collected from the rhizosphere of cultivated olive (*Olea europaea* ssp. *europaea* L.) at Mirandela, Bragança, Portugal (41.53099 N latitude, −7.047058 W longitude) by J.E. Palomares-Rius and P. Castillo, mounted in pure glycerine and deposited in the Nematode collection of the Laboratorio de Fitonematología, Institute for Sustainable Agriculture, CSIC, Spain (slide numbers SO18_3- SO18_12); and two female paratypes at the USDA Nematode Collection, Beltsville, MD, USA (collection number T-8307p).

#### Etymology

The species epithet refers a noun derived from Greek *kryptos* (“hidden”) and the existing species *duriense*. It refers to the cryptic nature of the new species, which is morphologically similar to *X. duriense* but genetically distinct.

#### Description

*Female.* Body medium-sized, tapering gradually towards the posterior end; habitus forming an open spiral when killed by gentle heat. Cuticle smooth 1.0–1.5 µm thick along most of the body, becoming progressively thickened towards the tail, where it reaches 7.0–9.0 µm. Lip region broadly rounded and set off from the body contour by a distinct constriction; amphidial fovea funnel-shaped, with a slit-like aperture 5.5–6.5 µm wide, located anterior to the constriction. Odontostyle moderately long, 1.7 (1.6–1.9) times the odontophore length, with well-developed flanges, 4.5–5.5 µm wide. Guiding ring double, guiding sheath 5.5–7.5 µm long. Pharynx composed of an anterior slender part, 190–291 µm long, followed by a well-demarcated cylindrical basal bulb, 63.5 (60–66) µm long and 13.0 (12.0–15.0) µm wide, occupying 23 (21–25)% of the total pharyngeal length. Glandularium 57 (55–59) µm long. Intestine simple; rectum 1.0–1.3 anal body diameters long. Reproductive system didelphic-amphidelphic, with both genital branches about equally developed. Ovaries reflexed; endosymbiotic bacteria not observed. Uteri short (*c*. 125 µm long) without any differentiation. Vulva clearly postequatorial, transverse slit-like, vagina perpendicular to the body axis, 11.0 (9.0–12.0) μm long. Sperm not observed in the uterus. Tail short, conoid, dorsally convex-conoid with a rounded terminus (Figs. 18, 19), bearing two caudal pores on each side.

*Male.* Not found.

*Juveniles.* Not found.

#### Diagnosis and relationships

*X. cryptoduriense* sp. nov. is a parthenogenetic species belonging to the *pachtaicum*-subgroup of the *X. americanum* group [50, 51]. It is primarily characterised by a medium body size (1399–1840 µm); lip region broadly rounded and set off from the body contour by a distinct constriction; a moderate long odontostyle and odontophore (66–72 and 38–42 µm, respectively); vulva located at 61–63% of body length; female tail short, conoid, dorsally convex-conoid with a rounded terminus. It is further distinguished by its unique sequences of the D2–D3 rRNA, ITS1 rRNA, and COI genes. The alpha-numeric identification codes of the new species according to the polytomous key of Lamberti et al. [76] are (exceptions in parentheses): A1(2)-B4–C4–D1–E2(1)-G2–H2–I3(2).

Based on morphometric data and the polytomous key Lamberti et al. [76], *X*. *cryptoduriense* sp. nov. is almost indistinguishable from the paratype and other reported populations of *X. duriense* from Portugal and Spain (Table 6), differing by lower a ratio (56.2–73.6 vs 68–81), and a lower c ratio (42.5–57.5 vs 53–67) [8, 28, 77, 78]. *Xiphinema cryptoduriense* sp. nov. is also close to *X. opisthohysterum* Siddiqi, 1961 [31], from which can be distinguished by a higher a ratio (56.2–73.6 vs 59–63), more posterior vulva (61–63 vs 57), slightly longer odontostyle (66–72 µm vs 64–68 µm) [7, 31, 75].

#### Ribosomal and mitochondrial DNA characterisation

The accessions from *X. cryptoduriense* sp. nov. showed high similarity values with *X. duriense*, in both ribosomal and partial mitochondrial COI markers. The similarity values found were: 97% for the D2–D3 region (differing by 20 nucleotides and 11 indels) and 92% for ITS1 region and partial COI, varying by 70 and 21 nucleotides and by 37 nucleotides and 0 indels, respectively. No GenBank accession available was found for the partial 18S rRNA of *X. duriense*. Finally, no intraspecific variability was detected for any of the markers analyzed in this study.

### Morphology, morphometrics and molecular characterisation of known *Xiphinema americanum*-group species

Four populations of *X. incertum* were detected (Table 1): two from Spain, which have already been well characterised both molecularly and morphometrically, and two from cultivated olive and black pine at Ait Taleb and Demnate (Azilal province), Morocco. These Moroccan records constitute the first reports of this species for the country. The Moroccan material agrees with the original and subsequent descriptions (Table S2, Fig. S5), showing only minor variation in the c’ ratio [27–29]. D2–D3 sequences (PZ458878–PZ458879) were almost identical (99.9–100%) to those from Spain and Bosnia-Herzegovina, and ITS sequences (PZ487679–PZ487680) were likewise highly similar (96.8%) to the single Spanish ITS sequence available in NCBI (JQ990044) [28, 29].

Two populations of *X. luci* were found in cultivated olive at Ait Ichou, Azilal and Ouarjdan, Fkih Ben Salah, Morocco, representing the first records of this species for the country. These populations match the diagnostic characters of the original and subsequent Spanish descriptions (Table S2, Fig. S6) [8, 75]. D2–D3 and COI sequences (PZ458880–PZ458882, PZ464604–PZ464606) showed very high similarity to sequences from Spain and Mexico (99.7–100%, 97.9–98.6%, respectively) [8, 79, 80].

Thirteen populations of *X. pachtaicum* were identified across all surveyed countries (Greece, Morocco, Portugal, Spain), in both cultivated olive and adjacent patches of natural vegetation (Table 1). Diagnostic morphometrics and molecular markers confirmed their identity, and no further analyses were required, as this species is widely distributed and well studied in the Mediterranean Basin [8, 28, 62, 68].

Two populations of *X. plesiopachtaicum* were detected in cultivated olive at Antequera, Málaga, Spain, and Mirandela, Bragança, Portugal. Although the species has been recorded at several Spanish localities (unpublished), the Mirandela population represents the first confirmed record for Portugal. This material agrees with the original Spanish description (Table S2, Fig. S7) [8]. D2–D3 and ITS sequence from the Portuguese population (PZ487681) were highly similar to Spanish sequences available in NCBI (98.9–99.3%, 97.1%, respectively) [8].

## Discussion

This study advances current understanding of dagger nematode diversity in Mediterranean olive agroecosystems and adjacent natural vegetation by integrating morphology, morphometrics and multilocus molecular data to delimit four new species, three within the *Xiphinema* non-*americanum* group and one within the *X. americanum* group. Previous surveys in Portugal, Spain, Greece and North Africa [7, 8, 10, 16, 62, 81] documented a rich *Xiphinema* fauna but often relied on morphology or single-locus markers, leading to ambiguous identifications and unresolved species boundaries [7, 10, 16]. Our results show that *Xiphinema* diversity in olive systems and adjacent natural vegetation has been underestimated, particularly within cryptic lineages of the *X.* non-*americanum* and *X. americanum*-groups. The recognition of *X. alentejanum* sp. nov. and *X. cryptoduriense* sp. nov. resolves long-standing uncertainties within the *X. baetica-X. andalusiense-X. macrodora* and *X. duriense-X. opisthohysterum* complexes, and suggests that these complexes may have diversified through repeated episodes of allopatric isolation in geographically structured landscapes [7, 8, 24]. The distribution of *X. alentejanum* sp. nov. and *X. cryptoduriense* sp. nov. along the western margin of the Iberian Peninsula reflects patterns observed in other nematode groups and soil invertebrates, where Atlantic-Mediterranean transition zones act as hotspots of endemism and cryptic speciation [7, 8, 82, 83]. These patterns are consistent with the role of the western Mediterranean as a long-term refugial area and centre of diversification for low-dispersal soil fauna [84, 85].

The biogeographical patterns identified in this study offer new insights into the diversification and distribution of *Xiphinema* species across the Mediterranean Basin, a region widely recognised as a global biodiversity hotspot shaped by a complex geological and climatic history, including Miocene tectonic restructuring, Pleistocene glacial-interglacial oscillations and long-term habitat fragmentation [84, 86, 87]. These processes have repeatedly promoted lineage isolation, persistence in refugia and subsequent secondary contact, generating the high levels of endemism and cryptic diversity characteristic of Mediterranean terrestrial biota [85]. Our findings indicate that dagger nematodes associated with olive agroecosystems and adjacent natural vegetation follow similar biogeographical trajectories with diversification patterns consistent with those observed in other soil-dwelling organisms across the region [88, 89].

Earlier studies highlighted morphological overlap and high intraspecific variability in these groups [7, 8, 50, 51], but lacked multilocus evidence. Here, ribosomal (D2–D3, ITS1) and mitochondrial (COI) markers, together with morphometric analyses and molecular species-delimitation statistics, provide robust support for the distinctiveness of the species examined [3, 8]. Our results reinforce the view that the partitioning of morphological variation into components attributable to shape and size is crucial for species delimitation in cryptic species complexes [90, 91], a conclusion that extends the complex diversity characteristic of soil animals, including plant-parasitic nematodes [38]. Remarkably, our analyses indicated that although overall morphological variation was largely explained by size-related variables, species discrimination within cryptic complexes depended primarily on shape-related characters (Fig. 2). This pattern aligns with established knowledge that body size varies relatively little among cryptic *Xiphinema* species, whereas female tail shape is a key diagnostic trait for distinguishing species within both the *X.* non-*americanum* group and *X. americanum* group [3, 6, 8, 66]. Notably, this trend is also evident in the morphology of juvenile developmental stages in cryptic species in *Xiphinema*, particularly the tail shape of the first juvenile stage [66]. Our findings confirm this pattern: cryptic species within the *X. baetica* species complex (i.e. *X. alentejanum* sp. nov. and *X. baetica*) differ not only in their overall body shape (i.e. *a* ratio), but more prominently in the tail morphology of the first juvenile stage (see species description). This represents an important avenue for future research into the eco-evolutionary development of morphological characters in cryptic complexes of soil nematodes, and particularly within the genus *Xiphinema* [90, 91].

The recovery of the new species from both cultivated olive and adjacent natural habitats reinforces earlier findings that Mediterranean cultivated and wild vegetation acts as a reservoir of *Xiphinema* diversity [3, 7]. This ecological continuity indicates that olive agroecosystems reflect broader regional biodiversity patterns rather than hosting isolated nematode communities, consistent with observations from southern Spain and Morocco where species such as *X. pachtaicum*, *X. italiae* and *X. diversicaudatum* occur in both wild and cultivated hosts [8, 92]. Likewise, the detection of *X. andalusomarocense* sp. nov. and *X. africana* sp. nov. in both cultivated olive and adjacent natural habitats in Spain and Morocco, further underscores the ecological continuity between olive agroecosystems and surrounding vegetation. These findings also reinforce the hypothesis that soil biodiversity of plant-parasitic nematodes is strongly linked with vegetation structure and composition [93]. Moreover, they support the view that the western Mediterranean, particularly the Iberian Peninsula and north-western Maghreb, constitutes a centre of diversification for *Xiphinema*, as previous studies have documented high species richness in these regions [3, 7, 8, 50, 51].

Our phylogenetic analyses support the presence of two well-defined species complexes within the *X.* non-*americanum* and *X. americanum*-groups, consistent with earlier hypotheses based on morphology and limited molecular datasets [6, 50, 51, 64]. By integrating multilocus phylogenies with species-delimitation statistics (PID, Rosenberg’s Pₐᵦ), this study provides the strongest evidence to date for the evolutionary independence of these species, overcoming the constraints of previous work that relied on single markers or incomplete sampling. Within the *X. americanum*-group, the first clade of the 28S rRNA phylogeny exhibits short branch lengths, suggesting either recent diversification or slower molecular evolution. The exclusion of ITS1 from our analyses further highlights the phylogenetic problems of this marker in the group: ITS1 sequences show extreme variability and poor alignment quality, as previously reported [6, 64]. The lack of homology between our ITS1 sequences and those in GenBank underscores the need for improved reference datasets and standardised sequencing protocols for this complex group.

Overall, our findings demonstrate that the Mediterranean Basin harbours greater *Xiphinema* diversity than previously recognised. The discovery of four new species and the clarification of two species complexes highlight the region’s role as a reservoir of hidden nematode diversity and emphasise the need for continued integrative research to fully understand the evolutionary and biogeographical dynamics of Mediterranean soil fauna [82]. These results refine the systematics of *Xiphinema*, expand current knowledge of nematode biodiversity in Mediterranean olive landscapes, and provide a robust foundation for future ecological, biogeographical and plant-health research.

## Conclusion

This study presents a comprehensive taxonomic framework for dagger nematodes inhabiting Mediterranean olive agroecosystems, leading to the identification of three new species within the *Xiphinema* non-*americanum*-group and one new species within the *X. americanum*-group. The integration of morphological, morphometric, and multilocus molecular evidence provides robust support for the distinctiveness of these taxa and reveals two well-defined species complexes among the populations examined. Ribosomal and mitochondrial markers consistently resolved species boundaries, while phylogenetic analyses clarified relationships among closely related lineages and uncovered previously unrecognised diversity. Collectively, these findings advance current understanding of *Xiphinema* biodiversity in the western Mediterranean Basin and highlight the value of integrative approaches for detecting cryptic species in morphologically conservative nematode groups. The recognition of these new species and complexes offers a refined framework for future ecological, biogeographical, and plant-health research involving dagger nematodes.

## Electronic supplementary material

Below is the link to the electronic supplementary material.

Supplementary Material 1

Supplementary Material 2: Fig. S1. Light micrographs of Xiphinema adenohystherum Lamberti et al. 1992, from Portugal. A, female lip region; B, detail of odontophore; C-D, detail of uterus showing numerous spines; E-G, female tail regions. Scale bars: A-G = 20 μm

Supplementary Material 3: Fig. S2. Light micrographs of Xiphinema nuragicum Lamberti et al. 1992 from Portugal. A-C, female lip region; D, detail of uterus and pseudo-Z-organ; E, F, female tail regions. Scale bars: A-F = 20 μm

Supplementary Material 4: Fig. S3. Light micrographs of Xiphinema zagrosense Ghaemi et al. 2012, from Greece. A, neck region. B, C, female lip regions. D, detail of excretory gland cell. E, F, detail of uterus with numerous large crystalloid bodies; G, H, female tail regions. Scale bars: A = 100 μm; B-H = 20 μm

Supplementary Material 5: Fig. S4. Light micrographs of Xiphinema italiae Meyl, 1953, from Morocco. A-D, female lip regions; E-G, female tail regions. Scale bars: A = 100 μm; B-D = 10 μm; F-G = 20 μm

Supplementary Material 6: Fig. S5. Light micrographs of Xiphinema incertum Lamberti et al. 1983, from Morocco. A, B, entire female; C, female neck region; D-F, female lip region. G, detail of odontophore. H, vulval region. I-K, female tail regions. Scale bars A = 500 μm; B = 100 μm; C = 50 μm; D = 10 μm E-K = 20 μm

Supplementary Material 7: Fig. S6. Light micrographs of Xiphinema luci Lamberti & Bleve-Zacheo, 1979 from Morocco. A, female lip region; B-C, female tail. Scale bars = 10 μm

Supplementary Material 8: Fig. S7. Light micrographs of Xiphinema plesiopachtaicum Archidona-Yuste et al. 20165b from Portugal. A, B, female lip region; C, vulval region; D, E, female tail. Scale bars A, C-E= 20 μm; B = 10 μm

## Data Availability

All data generated or analysed during this study are included in this published article. Sequences are deposited in GenBank, NCBI. This article has been registered at ZooBank (http://zoobank.org/urn:lsid:zoobank.org:pub: BC04CEA0-3347-4D07-9355-893826EFEEC3).
