# Supplementary material for: Resolving species boundaries in Mediterranean Xiphinema (Nematoda: Longidoridae) through multivariate morphometrics and multilocus profiling: four new species and two cryptic complexes
Source: Zoological Lett. 2026 Sep 22;12:19. doi: 10.1186/s40851-026-00269-4 (PMC13653414; doi:10.1186/s40851-026-00269-4)
Supplement: Supplementary file 1 [file 40851_2026_269_MOESM1_ESM.docx]

**Table S1.** Morphometrics of known *Xiphinema* non-*americanum*-group species from Mediterranean olive groves. All measurements are in µm, except for body length in mm and in the form: mean ± s.d. (range).

| Species | *X. adenohystherum* | |  | *X. italiae* | |  | *X. nuragicum* | |  | *X. zagrosense* |
| --- | --- | --- | --- | --- | --- | --- | --- | --- | --- | --- |
| Sample code | SO22 | SO21 |  | ZN42 | ZN43 |  | SO20 | SO20 |  | AN2 |
| Host-plant | holly oak | yellow broom |  | Indian fig opuntia | wild olive |  | cultivated olive | holly oak |  |  |
| Locality, country | Ferreira do Alentejo, Portugal | Ferreira do Alentejo, Portugal |  | Meknes, Morocco | Meknes, Morocco |  | Braganza, Braganza, Portugal | Braganza, Braganza, Portugal |  | Lesbos, Greece |
| Character/ratio^1^ | Females | Females |  | Females | Females |  | Females | Females |  | Females |
| n | 4 | 3 |  | 4 | 3 |  | 4 | 4 |  | 3 |
| L | 5.255 ± 0.287 | 4.817 ± 0.122 |  | 2.933 ± 0.083 | 2.964 ± 0.047 |  | 3.918 ± 0.252 | 3.965 ± 0.076 |  | 4.214 ± 0.203 |
|  | (4.978-5.657) | (4.677-4.900) |  | (2.829-3.020) | (2.933-3018) |  | (3.629-4.163) | (3.877-4.039) |  | (4.008-4.414) |
| a | 79.3 ± 4.3 | 69.8 ± 1.0 |  | 82.9 ± 8.5 | 82.7 ± 10.9 |  | 54.8 ± 5.7 | 55.9 ± 2.3 |  | 61.9 ± 1.2 |
|  | (76.7-85.7) | (68.8-80.7) |  | (70.2-88.2) | (70.2-89.2) |  | (49.0-60.2) | (53.5-58.5) |  | (60.7-63.1) |
| b | 8.5 ± 0.4 | 9.0 ± 0.2 |  | 8.5 ± 0.6 | 8.6 ± 0.4 |  | 8.0 ± 0.3 | 7.8 ± 0.5 |  | 7.3 ± 0.3 |
|  | (8.1-8.9) | (8.8-9.1) |  | (8.1-9.4) | (8.2-8.9) |  | (7.6-8.3) | (7.3-8.4) |  | (7.0-7.6) |
| c | 110.8 ± 5.6 | 110.6 ± 7.9 |  | 37.8 ± 0.5 | 38.7 ± 0.9 |  | 94.9 ± 7.2 | 96.9 ± 4.4 |  | 102.0 ± 5.1 |
|  | (105.6-118.5) | (101.7-116.7) |  | (37.2-38.2) | (37.6-39.2) |  | (85.2-102.4) | (91.3-102.0) |  | (97.8-107.7) |
| c' | 1.0 ± 0.1 | 1.0 ± 0.05 |  | 2.6 ± 0.1 | 2.6 ± 0.1 |  | 0.7 ± 0.1 | 0.7 ± 0.05 |  | 0.8 ± 0.01 |
|  | (0.9-1.1) | (1.0-1.0) |  | (2.5-2.7) | (2.5-2.8) |  | (0.7-0.8) | (0.7-0.8) |  | (0.8-0.9) |
| d | 8.3 ± 0.3 | 8.2 ± 0.6 |  | 8.3 ± 0.4 | 8.3 ± 0.47 |  | 8.9 ± 0.5 | 8.8 ± 0.5 |  | 9.1 ± 0.4 |
|  | (7.9-8.6) | (7.5-8.6) |  | (7.7-8.6) | (7.8-8.7) |  | (8.4-9.5) | (8.4-9.5) |  | (8.8-9.4) |
| d' | 2.7 ± 0.03 | 2.6 ± 0.1 |  | 2.5 ± 0.2 | 2.5 ± 0.3 |  | 3.4 ± 0.1 | 3.3 ± 0.1 |  | 2.9 ± 0.1 |
|  | (2.6-2.7) | (2.6-2.7) |  | (2.3-2.7) | (2.2-2.8) |  | (3.2-3.5) | (3.2-3.4) |  | (2.8-3.1) |
| V | 49.0 ± 1.0 | 47.9 ± 1.2 |  | 45.6 ± 1.1 | 44.4 ± 1.6 |  | 48.9 ± 1.5 | 48.4 ± 3.7 |  | 48.2.6 ± 0.4 |
|  | (47.5-49.6) | (46.5-48.7) |  | (44.5-47.1) | (42.7-45.8) |  | (47.4-50.9) | (43.8-52.7) |  | (47.8-48.5) |
| G_1_ | 9.5 ± 0.8 | 9.4 ± 0.8 |  | 11.7 ± 1.4 | 11.3 ± 0.9 |  | 11.8 ± 1.5 | 10.4 ± 0.8 |  | 10.4 ± 1.2 |
|  | (8.8-10.5) | (8.6-10.0) |  | (10.0-13.1) | (10.4-12.2) |  | (10.3-13.8) | (10.3-13.8) |  | (9.4-11.7) |
| G_2_ | 9.4 ± 1.0 | 9.4 ± 0.8 |  | 11.5 ± 1.1 | 11.2 ± 0.9 |  | 11.6 ± 1.3 | 10.3 ± 0.8 |  | 10.6 ± 1.6 |
|  | (8.7-10.8) | (8.4-10.0) |  | (10.1-12.4) | (10.3-12.0) |  | (10.2-13.4) | (9.3-11.1) |  | (9.3-12.5) |
| Odontostyle | 146.3 ± 3.3 | 145.7 ± 4.7 |  | 96.3 ± 2.2 | 95.3 ± 2.5 |  | 153.5 ± 2.6 | 151.5 ± 1.3 |  | 155.0 ± 2.6 |
|  | (142.0-150.0) | (142.0-151.0) |  | (94.0-99.0) | (93.0-98.0) |  | (150.0-156.0) | (150.0-153.0) |  | (152.0-157.0) |
| Odontophore | 88.3 ± 4.6 | 86.3 ± 3.8 |  | 56.3 ± 2.6 | 57.3 ± 2.1 |  | 89.0 ± 2.2 | 88.0 ± 2.2 |  | 93.3 ± 0.6 |
|  | (82.0-93.0) | (82.0-89.0) |  | (54.0-60.0) | (55.0-59.0) |  | (87.0-92.0) | (86.0-91.0) |  | (93.0-94.0) |
| Total stylet | 234.5 ± 6.6 | 232.0 ± 6.6 |  | 152.5 ± 4.7 | 152.7 ± 3.8 |  | 242.5 ± 4.7 | 239.5 ± 3.1 |  | 248.3 ± 3.1 |
|  | (228.0-243.0) | (226.0-239.0) |  | (148.0-159.0) | (150.0-157.0) |  | (237.0-248.0) | (237.0-244.0) |  | (245.0-251.0) |
| Lip region diam. | 16.3 ± 0.5 | 16.2 ± 0.3 |  | 10.1 ± 0.3 | 10.2 ± 0.3 |  | 15.3 ± 1.0 | 15.5 ± 1.3 |  | 15.5 ± 0.5 |
|  | (16.0-17.0) | (16.0-16.5) |  | (10.0-10.5) | (10.0-10.5) |  | (14.0-16.0) | (15.0-16.0) |  | (15.0-16.0) |
| Oral aperture to guiding ring | 134.8 ± 1.7 | 133.0 ± 5.0 |  | 83.5 ± 2.1 | 84.7 ± 2.5 |  | 136.0 ± 7.7 | 136.0 ± 6.7 |  | 141.0 ± 1.0 |
|  | (133.0-137.0) | (128.0-138.0) |  | (81.0-86.0) | (82.0-87.0) |  | (126.0-143.0) | (127.0-142.0) |  | (140.0-142.0) |
| Max. body diam. | 66.3 ± 1.7 | 69.0 ± 1.0 |  | 35.8 ± 4.9 | 36.3 ± 5.8 |  | 72.0 ± 7.8 | 71.0 ± 3.2 |  | 68.0 ± 2.0 |
|  | (64.0-68.0) | (68.0-70.0) |  | (33.0-43.0) | (33.0-43.0) |  | (64.0-82.0) | (68.0-75.0) |  | (66.0-70.0) |
| Tail length | 47.5 ± 3.1 | 43.7 ± 2.1 |  | 77.5 ± 1.3 | 76.7 ± 1.5 |  | 41.4 ± 2.9 | 41.0 ± 2.2 |  | 41.3 ± 0.6 |
|  | (44.0-51.0) | (42.0-46.0) |  | (76.0-79.0) | (75.0-78.0) |  | (37.0-43.0) | (38.0-43.0) |  | (41.0-42.0) |
| J | 9.6 ± 0.5 | 9.7 ± 0.6 |  | 11.5 ± 1.3 | 11.0 ± 1.0 |  | 11.6 ± 1.3 | 11.5 ± 1.0 |  | 8.8 ± 1.3 |
|  | (9.0-10.0) | (9.0-10.0) |  | (10.0-13.0) | (10.0-12.0) |  | (10.0-13.0) | (10.0-12.0) |  | (7.5-10.0) |

^1^ Abbreviations as defined: a, body length/maximum body width; b, body length/pharyngeal length; c, body length/tail length; c', tail length/body width at anus; V (distance from anterior end to vulva/body length) x 100; d = anterior to guiding ring/body diam. at lip region; d’ = body diam. at guiding ring/body diam. at lip region; J = hyaline tail region length.

**Table S2.** Morphometrics of *Xiphinema americanum*-group species from olive (*Olea europaea* subspp*. europaea*) groves in Morocco and Portugal. All measurements are in µm and in the form: mean ± s.d. (range), except where original literature sources provide data in alternative formats.

| Species | *Xiphinema incertum* | |  | *Xiphinema luci* | |  | *Xiphinema plesiopachtaicum* |
| --- | --- | --- | --- | --- | --- | --- | --- |
| Locality, province, country | Ait Taleb, Azilal, Morocco | Demnate, Azilal, Morocco |  | Ait Ichou, Azilal, Morocco | Ouarjdan, Fkih Ben Salah, Morocco |  | Mirandela, Braganza, Portugal |
| Sample code | SO36 | ZN13 |  | SO37 | SO39 |  | SO18 |
| Character/ratio^1^ | Females | Females |  | Females | Females |  | Females |
| n | 4 | 3 |  | 3 | 3 |  | 5 |
| L (µm) | 1847 ± 38 | 1875 ± 42 |  | 1756 ± 44 | 1829 ± 172 |  | 1964 ± 157 |
|  | (1811-1895) | (1841-1922) |  | (1716-1803) | (1658-2001) |  | (1794-2172) |
| a | 49.4 ± 3.7 | 50.8 ± 2.5 |  | 48.8 ± 1.2 | 50.3 ± 2.7 |  | 64.6 ± 3.2 |
|  | (44.6-52.5) | (48.3-53.4) |  | (47.7-50.0) | (47.4-52.7) |  | (60.8-67.9) |
| b | 9.4 ± 1.2 | 10.4 ± 0.9 |  | 8.4 ± 0.2 | 7.9 ± 0.3 |  | 6.5 ± 0.5 |
|  | (7.9-10.9) | (9.3-11.0) |  | (8.2-8.6) | (7.6-8.2) |  | (6.2-7.3) |
| c | 63.5 ± 2.1 | 66.2 ± 2.6 |  | 58.2 ± 4.7 | 62.3 ± 2.0 |  | 78.7 ± 8.0 |
|  | (61.8-66.5) | (63.5-68.6) |  | (54.6-63.6) | (60.9-64.5) |  | (69.0-88.7) |
| c' | 1.1 ± 0.2 | 1.2 ± 0.1 |  | 1.2 ± 0.1 | 1.1 ± 0.02 |  | 1.4 ± 0.1 |
|  | (0.9-1.3) | (1.1-1.3) |  | (1.1-1.3) | (1.1-1.2) |  | (1.3-1.5) |
| V | 52.6 ± 1.1 | 52.7 ± 1.5 |  | 51.2 ± 0.3 | 51.3 ± 0.6 |  | 56.5 ± 0.6 |
|  | (51.5-54.0) | (51.0-54.0) |  | (51.0-51.5) | (51.0-52.0) |  | (56.0-57.5) |
| Odontostyle | 91.1 ± 3.1 | 94.2 ± 0.8 |  | 91.0 ± 3.5 | 93.0 ± 2.0 |  | 81.5 ± 1.8 |
|  | (88.0-94.0) | (93.5.0-95.0) |  | (89.0-95.0) | (91.0-95.0) |  | (78.5-83.5) |
| Odontophore | 50.0 ± 3.8 | 50.5 ± 3.0 |  | 49.7 ± 4.6 | 50.7 ± 3.1 |  | 48.1 ± 3.3 |
|  | (46.0-53.5) | (47.5-53.5) |  | (47.0-55.0) | (48.0-54.0) |  | (43.0-51.0) |
| Lip region diam. | 9.3 ± 0.5 | 9.2 ± 0.6 |  | 10.7 ± 0.3 | 10.7 ± 0.3 |  | 9.7 ± 0.8 |
|  | (8.5-9.5) | (8.5-9.5) |  | (10.5-11.0) | (10.5-11.0) |  | (8.5-10.5) |
| Oral aperture to guiding ring | 75.6 ± 3.8 | 76.5 ± 5.8 |  | 72.7 ± 4.7 | 73.3 ± 5.0 |  | 68.9 ± 4.5 |
|  | (70.0-82.0) | (70.0-81.0) |  | (69.0-78.0) | (68.0-78.0) |  | (63.0-75.0) |
| Tail length | 29.1 ± 1.1 | 28.3 ± 0.6 |  | 30.3 ± 3.1 | 29.3 ± 2.1 |  | 25.0 ± 0.9 |
|  | (28.0-30.5) | (28.0-29.0) |  | (27.0-33.0) | (27.0-31.0) |  | (24.0-26.0) |
| J | 7.2 ± 1.2 | 6.2 ± 0.6 |  | 7.5 ± 0.5 | 7.7 ± 0.3 |  | 6.9 ± 1.1 |
|  | (6.5-8.5) | (5.5-6.5) |  | (7.0-8.0) | (7.5-8.0) |  | (6.0-8.5) |

^1^ Abbreviations as defined: a, body length/maximum body width; b, body length/pharyngeal length; c, body length/tail length; c', tail length/body width at anus; V (distance from anterior end to vulva/body length) x 100; d = anterior to guiding ring/body diam. at lip region; d’ = body diam. at guiding ring/body diam. at lip region; J = hyaline tail region length.
